# A sub-10-nm, folic acid-conjugated gold nanoparticle as self-therapeutic treatment of tubulointerstitial fibrosis

**DOI:** 10.1073/pnas.2305662120

**Published:** 2023-10-09

**Authors:** Cecilia Ka Wing Chan, Cheuk Chun Szeto, Leo Kit Cheung Lee, Yu Xiao, Bohan Yin, Xiaofan Ding, Thomas Wai Yip Lee, James Yun Wong Lau, Chung Hang Jonathan Choi

**Affiliations:** ^a^Department of Surgery, The Chinese University of Hong Kong, Shatin, New Territories, Hong Kong; ^b^Department of Medicine and Therapeutics, The Chinese University of Hong Kong, Shatin, New Territories, Hong Kong; ^c^Department of Biomedical Engineering, The Chinese University of Hong Kong, Shatin, New Territories, Hong Kong; ^d^School of Pharmacy, The Chinese University of Hong Kong, Shatin, New Territories, Hong Kong

**Keywords:** nanomedicine, gold nanoparticle, chronic kidney disease, fibrosis, renal tubules

## Abstract

Nanomedicines for treating chronic kidney disease (CKD) are on the horizon, yet their delivery to renal tubules where tubulointerstitial fibrosis occurs remains inefficient. We report a folic acid-conjugated gold nanoparticle that can transport into renal tubules and treat tubulointerstitial fibrosis in mice with unilateral ureteral obstruction. The 3-nm gold core allows for the dissection of bio–nano interactions in the fibrotic kidney, ensures the overall nanoparticle (~7 nm) to be small enough for glomerular filtration, and naturally inhibits the p38α mitogen-activated protein kinase in the absence of chemical or biological drugs. The folic acids support binding to selected tubule cells with overexpression of folate receptors and promote retention in the fibrotic kidney. Upon intravenous injection, this nanoparticle can selectively accumulate in the fibrotic kidney over the nonfibrotic contralateral kidney at ~3.6% of the injected dose. Delivery to the fibrotic kidney depends on nanoparticle size and disease stage. Notably, a single injection of this self-therapeutic nanoparticle reduces tissue degeneration, inhibits genes related to the extracellular matrix, and treats fibrosis more effectively than standard Captopril therapy. Our data underscore the importance of constructing CKD nanomedicines based on renal pathophysiology.

## Significance

Chronic kidney disease (CKD) affects ~10% of adults worldwide. Prolonged renal fibrosis leads to CKD and ultimately kidney failure that requires dialysis or transplantation. Current drugs can retard disease progression, but there are no specific treatments for tubulointerstitial fibrosis, a common manifestation of end-stage CKD. Nanomedicines hold immense potential for treating CKD, but their delivery to renal tubules is challenging, and many past examples were preventive. Leveraging the overexpression of folate receptors on selected tubule cells in the fibrotic kidney, this sub-10-nm folic acid–conjugated gold nanoparticle can selectively accumulate in the fibrotic kidney, enter tubules, inhibit CKD-related kinases, and treat tubulointerstitial fibrosis after disease establishment. Consideration of pathophysiology and adoption of self-therapeutic nanoparticles will jointly inspire effective treatments.

Renal fibrosis represents a failed wound-healing process in the kidney after sustained injury. Left untreated, it can lead to chronic kidney disease (CKD) and ultimately kidney failure (1). Renal fibrosis is characterized by excessive accumulation of extracellular matrix (ECM), infiltration of inflammatory cells, and a loss of kidney tissue. Developing treatments for renal fibrosis is an unmet clinical need as there is no specific treatment. Immunosuppressive (2) and blood pressure control drugs (3) can only retard the progression of renal fibrosis, but their side effects include hypotension and hyperkalemia.

Nanoparticles (NPs) are promising drug carriers to the kidneys because their delivery is tailorable by adjusting NP physicochemical properties (4, 5). However, past reports on using NPs for alleviating renal fibrosis were relatively uncommon (with merely ~20 reports to date; *SI Appendix*, Fig. S1 and Table S1), partly due to their inefficient delivery to renal tubules (6), the major site of tubulointerstitial fibrosis (7). Of all CKD etiologies, tubulointerstitial fibrosis is a common histological manifestation of late-stage CKD and a strong predictor of disease progression (8). Past researchers employed large NPs (≥100 nm in diameter) loaded with siRNA (9) and small molecules (10) for passive delivery or folic acid (FA)-containing NPs to bind to folate receptors (FRs) on the apical membrane of tubular cells to achieve retention in the kidney (11), but these NPs need to disassemble unpredictably in the bloodstream to cross the glomerular filtration barrier [GFB; with a typical cutoff size of <10 nm (12)] before reaching the renal tubules. Conversely, ultrasmall NPs (≤5 nm) can efficiently penetrate the GFB upon intravenous (i.v.) injection in healthy animals (13) and models with renal fibrosis (14), but they were used only as in vivo contrast agents intended for fast urine clearance instead of as therapeutics where retention in the kidney is critical (15). Summing up, an NP large enough to contain antifibrotic drugs and tubule-targeting ligands while small enough to penetrate the GFB for accessing renal tubules remains elusive.

**Fig. 1.**
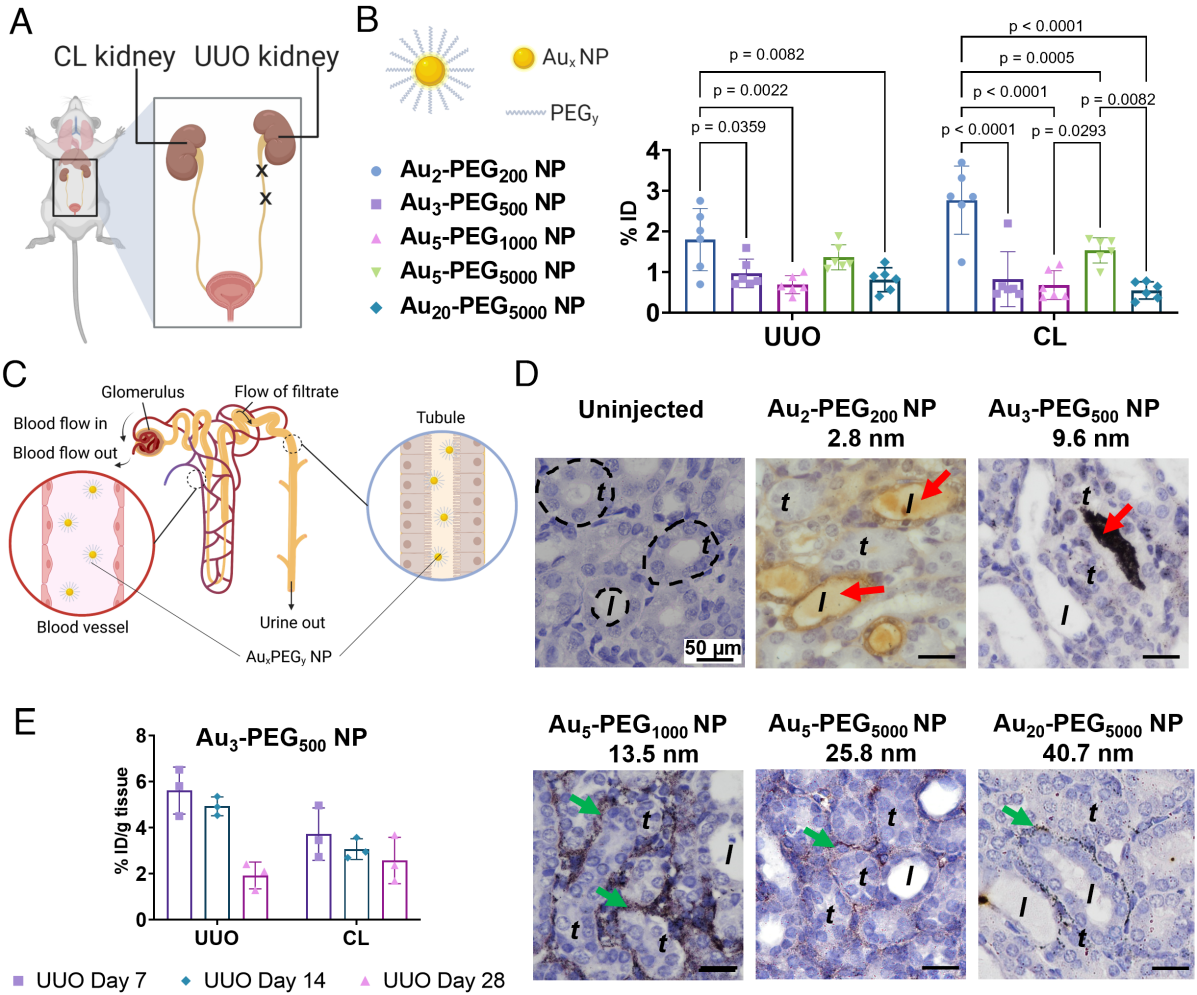
Passive delivery of Au_x_-PEG_y_ NPs to the fibrotic kidney. (*A*) UUO in mice is obtained by ligating the left ureter twice with sutures. (*B*) Distribution of Au_x_-PEG_y_ NPs in the UUO kidney and contralateral (CL) kidney 24 h postinjection into mice on day 7 post-UUO surgery. %ID = percentage of injected dose. Data are from n = 5 to 6, across one experiment. Statistical significance was evaluated using One-Way ANOVA with Tukey’s post hoc test for multiple comparison. All *P* values ≤ 0.05 are displayed on the graph. All bars and error bars represent mean ± SD. (*C*) Schematic illustration of kidney anatomy. Each nephron contains two functional subunits, namely glomerulus and the tubular system. The left *Inset* shows the flow of systemically injected NPs in the blood vessels that surround kidney tubules (or peritubular capillaries) and the right *Inset* shows the flow of NPs in the lumen of a renal tubule. (*D*) Silver enhancement staining of UUO kidney tissues showed that smaller Au_2_-PEG_200_ and Au_3_-PEG_500_ NPs could be found in the tubule lumen (red arrow), but larger Au_5_-PEG_1000_, Au_5_-PEG_5000_, and Au_20_-PEG_5000_ NPs localized in peritubular capillaries (green arrow). Yellow or black dots: Au_2_ NPs; black dots: AuNPs of larger sizes. The negative control histological image of the fibrotic kidney from uninjected mice shows the lack of detectable silver signals. Representative images from three kidney sections from each mouse, n = 3 mice/group. Representative locations of the renal tubule and tubule lumen were annotated as “t” and “l”, respectively. (*E*) Distribution of Au_3_-PEG_500_ NPs to the UUO and CL kidneys 24 h postinjection as a function of disease stage. Data are presented in terms of %ID/ g of kidney tissue. Data are from n = 3, across one experiment.

We report ~7-nm FA-conjugated gold NPs for bypassing the delivery hurdle to renal tubules and treating tubulointerstitial fibrosis. The 3-nm gold core serves three functions, namely i) supporting the dissection of the bio–nano interactions of NPs with the fibrotic kidney, ii) keeping the overall NP <10 nm to balance both GFB passage and kidney retention, and iii) surprisingly, acting as a self-therapeutic agent for inhibiting p38α mitogen-activated protein kinase (MAPK) without using chemical or biological drugs. Activation of p38α plays a vital role in renal fibrosis and acts downstream of transforming growth factor-β, a mediator of ECM deposition (16), therefore inhibition of p38α represents a suitable strategy for the pharmacological intervention of renal fibrosis. The conjugated FA molecules empower binding to FRs on renal tubules. We identify the localized upregulation of FRs on some tubules of the fibrotic kidney in CKD patients and in mice with unilateral ureteral obstruction (UUO) (Fig. 1*A*), an established animal model for tubulointerstitial fibrosis (17). Upon i.v. injection into UUO mice, this FA-conjugated gold NP accumulates preferentially in the fibrotic kidney (3.6% of injected dose; %ID) over the nonfibrotic contralateral (CL) kidney (0.6% ID). After disease establishment, a single injection of the NP reduces tissue degeneration, down-regulates genes linked to collagen-related ECM, and treats fibrosis more effectively than Captopril, a standard blood pressure control drug for alleviating CKD (18).

## Results and Discussion

### Effects of Disease Stage and NP Size on Passive Delivery to the Fibrotic Kidney.

Although UUO is a primary model of interstitial fibrosis, past evidence suggests that glomerular permeability is also affected (19, 20), potentially affecting the glomerular passage of NPs. Tubulointerstitial fibrosis irrespective to the initial cause affects glomerular hemodynamics and permeability, presumably via tubuloglomerular feedback. Therefore, it is imperative that the optimal NP size and disease stage be determined for delivery to the tubules of the fibrotic kidney. We prepared polyethylene glycol (PEG)-coated gold NPs (Au_x_-PEG_y_ NPs) by reacting gold cores of different diameters (x in nm; *SI Appendix*, Fig. S2) with thiolated PEG strands of various molecular weights (y in Da). For NP characterization, we negatively stained the NPs for transmission electron microscopy (TEM) imaging and observed that their overall physical diameters (gold core plus outer PEG shell; *SI Appendix*, Figs. S3 and S4) are similar to their hydrodynamic sizes as measured by dynamic light scattering. The only exception was Au_2_-PEG_200_ NP, its PEG shell did not have enough contrast to be imaged. (Typically, the TEM imaging contrast of sub-10-nm NPs was lower than that of the larger NPs probably because of its smaller Au core sizes and shorter PEG chain length.) This result indicates a dense coverage of PEG strands on the gold NP, consistent with literature precedent (21). We verified that the PEG coverage density is ≥2 PEG strands per nm^2^ of gold surface using three methods, namely direct displacement of thiolated PEG strands from Au_x_-PEG_y_ NP via dithiothreitol-induced aggregation of gold NPs (*SI Appendix*, Table S2) (22), indirect measurement of excess thiolated PEG strands unattached to the gold core during synthesis of Au_x_-PEG_y_ NP (*SI Appendix*, Table S3*A*) (23), and direct measurement of the organic content of Au_x_-PEG_y_ NPs using thermogravimetric analysis (TGA; *SI Appendix*, Table S3*B* and *SI Appendix*, Fig. S5) (24). All NPs were colloidally stable in 50% fetal bovine serum (FBS) for 24 h (*SI Appendix*, Figs. S6 and S7 and Table S4) and were not cytotoxic to primary renal tubule cells isolated from healthy Balb/c mice (*SI Appendix*, Fig. S8).

**Fig. 2.**
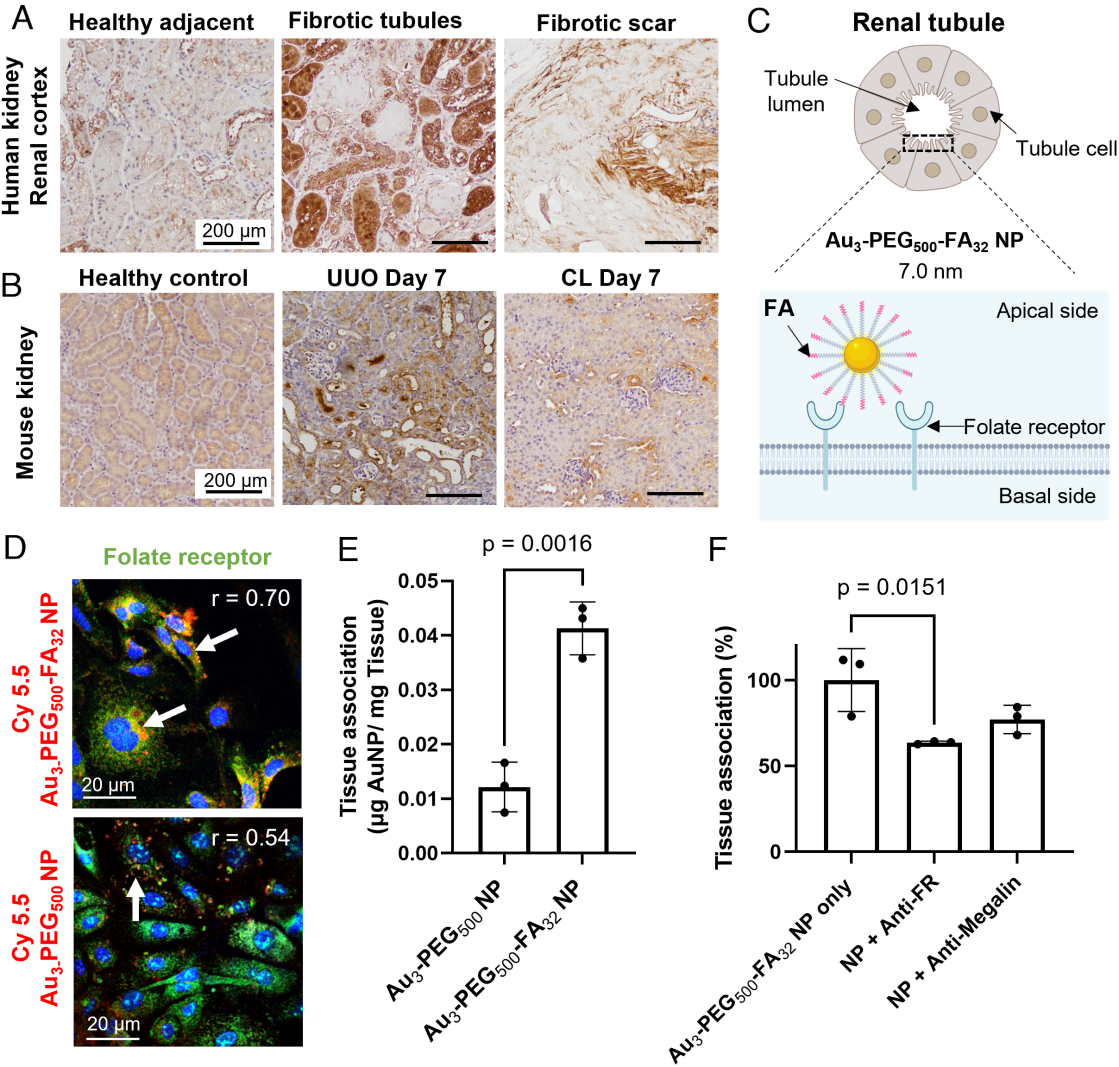
In vitro and ex vivo binding of Au_3_-PEG_500_-FA_32_ NPs to folate receptor (FR) on renal tubules after 2 h postincubation of cells or tissue blocks with NPs. (*A*) IHC staining of kidney biopsies from patients with renal fibrosis showed that some fibrotic tubules have higher expression of FR (brown) than adjacent healthy tissues. Representative images from two kidney sections from each tissue group (healthy adjacent vs. fibrotic tubules vs. fibrotic scar). (*B*) In mice, IHC staining showed that the elevated expression of FR (brown) in selected tubule cells in the UUO kidney is distinct from the diffuse, homogenous FR expression in healthy and CL kidneys. Representative images from three kidney sections from each mouse, n = 3 mice/group. (*C*) Schematic of Au_3_-PEG_500_-FA_32_ NP binding to FRs on the lumen side of a tubule cell. (*D*) Representative confocal images show stronger in vitro association of Cy5.5-labeled Au_3_-PEG_500_-FA_32_ NP (red; with white arrows) to FR (green) on healthy mouse primary renal tubule cells than Au_3_-PEG_500_ NP. r: Pearson’s colocalization coefficient. (*E*) Ex vivo association of Au_3_-PEG_500_ and Au_3_-PEG_500_-FA_32_ NPs to freshly diced UUO kidney blocks. Statistical significance was evaluated using the Mann–Whitney *U* test. Error bar denotes 1 SD, with n = 3 across one experiment. (*F*) Ex vivo competitive binding assay. Diced UUO kidney blocks were incubated with Au_3_-PEG_500_-FA_32_ NP with or without antibody against FR or megalin. Statistical significance was evaluated using one-way ANOVA with Tukey’s post hoc test for multiple comparison. All *P* values ≤ 0.05 are displayed on the graphs. All bars and error bars represent mean ± SD.

**Fig. 3.**
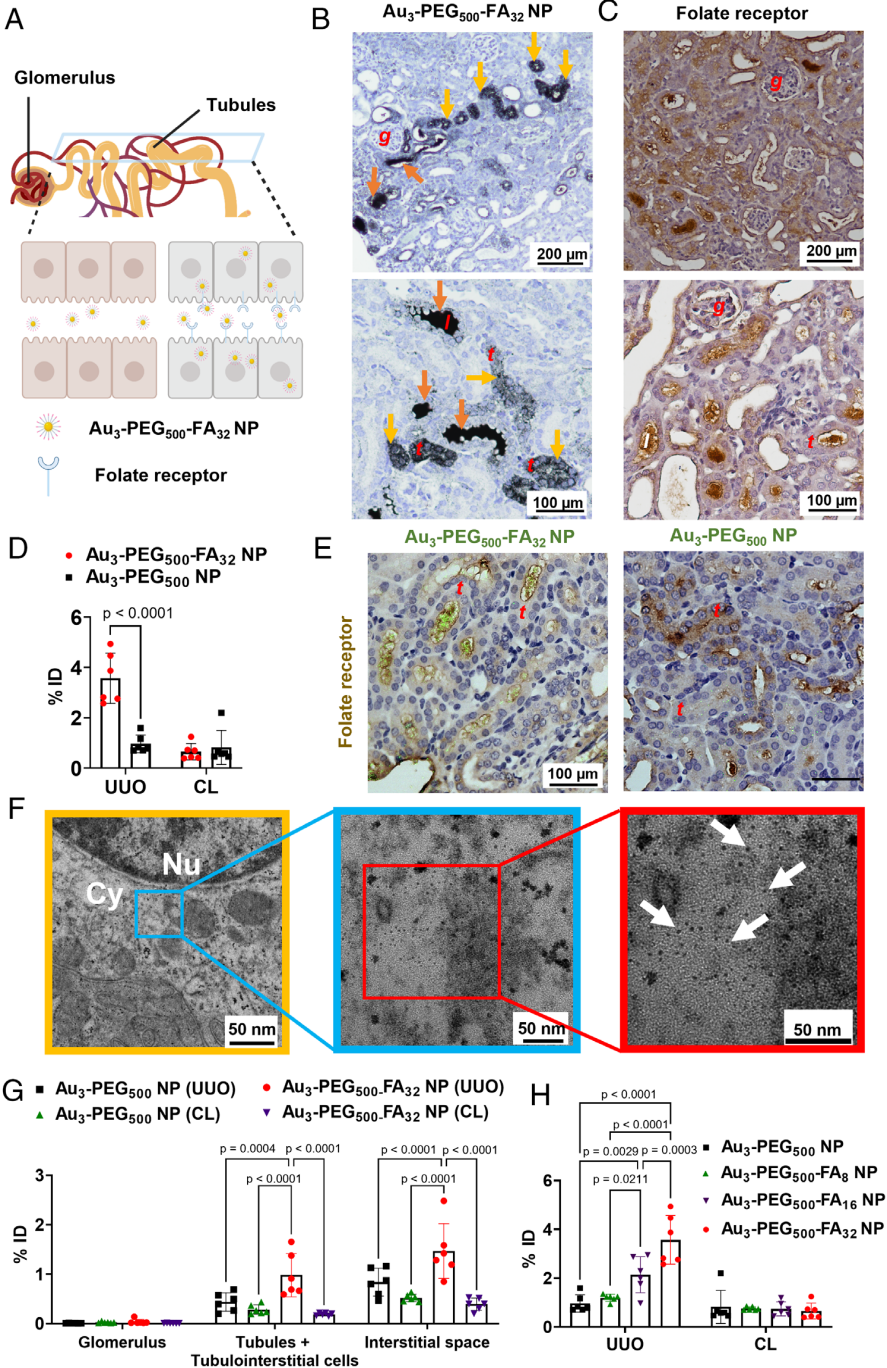
In vivo binding of Au_3_-PEG_500_-FA_32_ NPs to FR on tubules for enhanced delivery to the fibrotic kidney 24 h postinjection into UUO mice (day 7 post-UUO surgery). (*A*) The schematic drawing portrays the flow of NPs in urine (filtered from glomerulus) along the renal tubule and the expression of some FRs on the apical side of renal tubules. (*B*) Light micrographs of silver-enhanced UUO kidney sections show localization of Au_3_-PEG_500_-FA_32_ NPs inside the lumen of tubules (orange arrow) and inside tubule cells (yellow arrow) for a selected fraction of tubules. (*C*) IHC staining shows that the inhomogeneous pattern of FR expression (brown) only found on selected tubule cells in the UUO kidney, resembling localization of Au_3_-PEG_500_-FA_32_ NPs in selected tubules (Fig. 3*A*). Representative images from n = 3 kidney sections from three mice/group. (*D*) ICP-MS results showed higher accumulation of Au_3_-PEG_500_-FA_32_ NPs in UUO kidneys than Au_3_-PEG_500_ NPs. %ID = percent injected dose. Data are from n = 6, across two experiments. (*E*) IHC staining of FR (brown) and confocal reflectance microscopy of UUO kidney sections revealed colocalization of Au reflectance signals (green) and FR on selected renal tubules for Au_3_-PEG_500_-FA_32_ NP, but not Au_3_-PEG_500_ NP. For (*B*), (*C*), and (*E*), locations of the glomerulus, renal tubule, and tubule lumen are annotated as “*g*”, “*t*”, and “*l*”; representative images from three kidney sections from each mouse, n = 3 mice/group. (*F*) Representative TEM images show the presence of Au_3_-PEG_500_-FA_32_ NPs (arrow) inside a tubule cell of the UUO kidney; higher magnification TEM images (*Right*) show the NPs as individual entities in the tubule. The middle picture shows the enlargement of the blue boxed area of the left (yellow frame), and the right picture shows the enlargement of the red boxed area of the middle (blue frame). Nu = nucleus, Cy = cytosol. (*G*) ICP-MS analysis of compartmentalized UUO and CL kidneys showed preferential localization of Au_3_-PEG_500_-FA_32_ NPs in the tubules and interstitial space of the UUO kidney. Data are from n = 6, across two experiments. (*H*) Accumulation of Au_3_-PEG_500_-FA_z_ NPs increased with FA loading (z) in the UUO kidney, not CL kidney. Data are from n = 3, across one experiment. For (*D*), (*G*), and (*H*), statistical significance was evaluated using two-way ANOVA with Tukey’s post hoc test for multiple comparison. All *P* values ≤ 0.05 are displayed on the graphs. All bars and error bars represent mean ± SD.

**Fig. 4.**
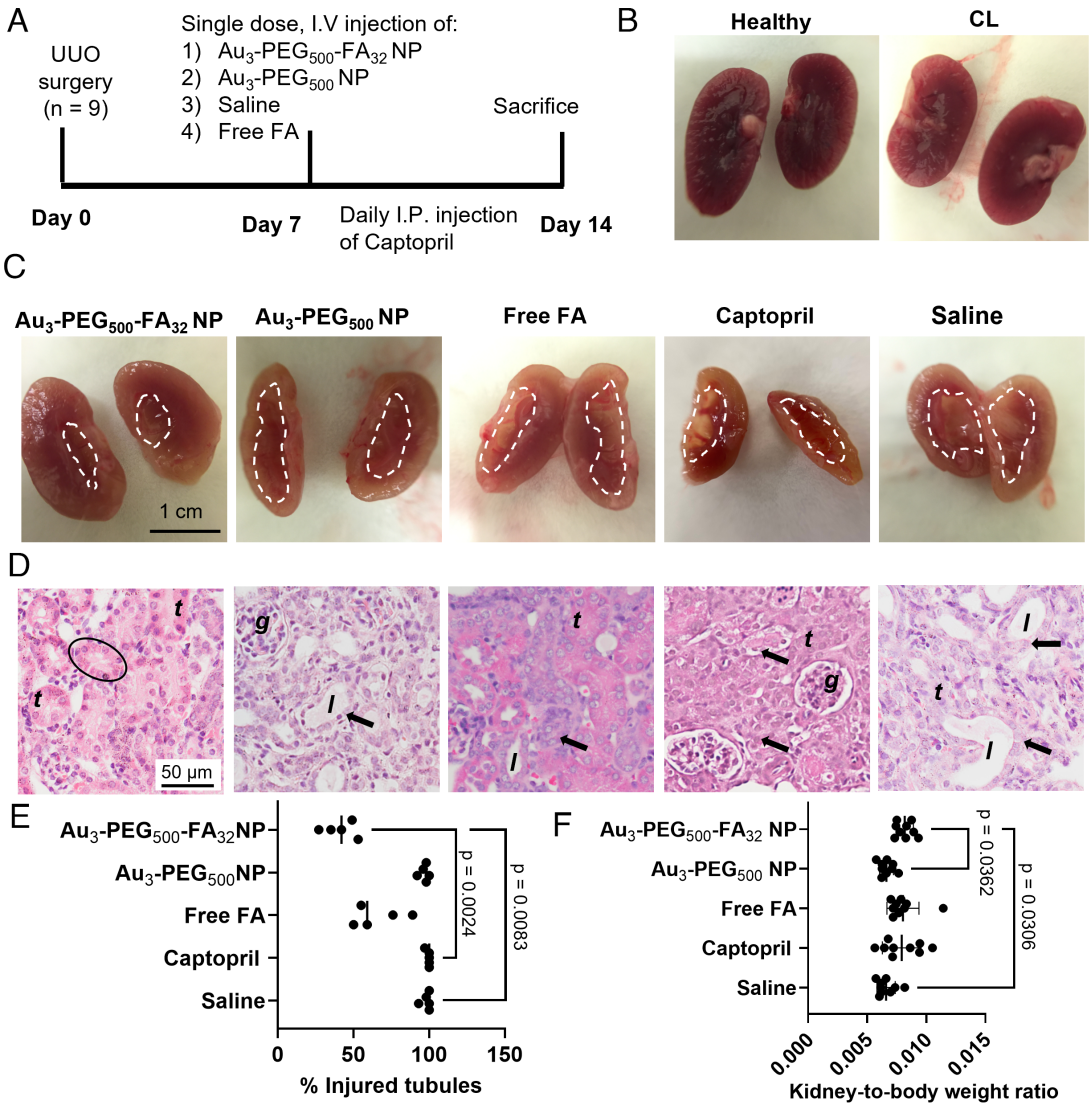
Au_3_-PEG_500_-FA_32_ NPs reduced kidney degeneration. (*A*) UUO mice were treated with a single i.v. dose of Au_3_-PEG_500_-FA_32_ NP, Au_3_-PEG_500_ NP, saline, or free FA on day 7 post-UUO surgery, or daily intraperitoneal injections of Captopril from day 7 to day 14. All animals were killed on day 14. (*B*) Gross appearance of a kidney from naive mice and a CL kidney from UUO mice after treatment with Au_3_-PEG_500_-FA_32_ NP. (*C*) UUO kidneys of mice treated with Au_3_-PEG_500_-FA_32_ NPs showed the least tissue degeneration. The area inside the dotted region indicates loss of tissue. (*D*) Histological images of UUO kidneys reveal that mice treated with Au_3_-PEG_500_-FA_32_ NPs had the least tubule injury (arrow). Circle: intact tubule. Representative images from three kidney sections from each mouse, n = 9 mice/group. Locations of the glomerulus, renal tubules, and tubule lumen are annotated as “*g*”, “*t*”, and “*l*”. Mice treated with Au_3_-PEG_500_-FA_32_ NP had significantly (*E*) lower % of injured tubules and (*F*) higher UUO kidney-to-body weight ratio than those treated with saline. For (*E*), statistical significance was evaluated using the nonparametric Kruskal–Wallis H test that does not require the assumption of normality. Data are from n = 5, across one experiment. For (*F*), statistical significance was evaluated using one-way ANOVA with Tukey’s post hoc test for multiple comparison. Error bars represent mean ± SD. Data are from n = 9, across two experiments. All *P* values ≤ 0.05 are displayed on the graph.

**Fig. 5.**
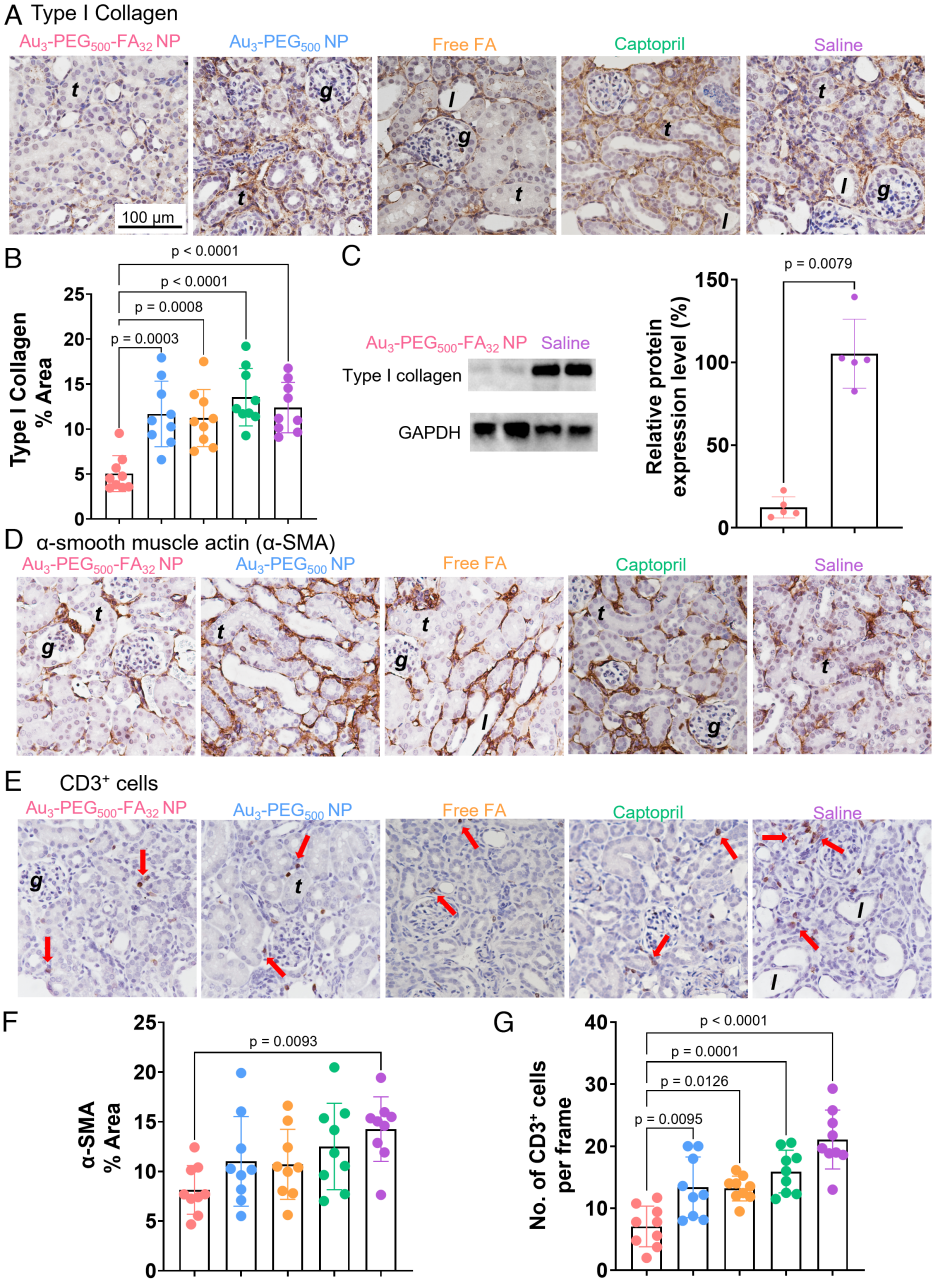
Au_3_-PEG_500_-FA_32_ NP reduced fibrosis in the UUO kidney. UUO mice were treated with either a single i.v. dose of Au_3_-PEG_500_-FA_32_, Au_3_-PEG_500_ NPs, saline, or free FA on day 7 post-UUO surgery, or daily intraperitoneal injections of Captopril from day 7 to day 14. All animals were killed on day 14. (*A*) IHC images show the expression of type I collagen (brown), the major component of ECM. (*B*) Quantitative analysis of type I collagen in kidney sections based on the IHC data in (*A*). (*C*) Western blot analysis revealed significant inhibition of type I collagen upon treatment with Au_3_-PEG_500_-FA_32_ NP. “100%” indicates the mean value of the type I collagen content from saline-treated mice. Data are from n = 5, across one experiment. Statistical significance was evaluated using the Mann–Whitney *U* test. IHC images show the expression of (*D*) α-SMA (brown) and (*E*) CD3^+^ cells (brown dot and red arrow). Quantitative analysis of (*F*) α-SMA (brown) and (*G*) CD3^+^ cells in the kidney sections based on the IHC data in (*D*) and (*E*). Mice treated with Au_3_-PEG_500_-FA_32_ NP had significantly lower (*B*) type I collagen area, (*F*) α-SMA area, and (*G*) CD3^+^ cells than those treated with saline. For (*A*), (*D*), and (*E*), representative images from three kidney sections from each mouse, n = 9 mice/group. Locations of the glomerulus, renal tubule, and tubule lumen are annotated as “*g*”, “*t*”, and “*l*”. For (*B*), (*F*), and (*G*), statistical significance was evaluated using one-way ANOVA with Tukey’s post hoc test for multiple comparison. All *P* values ≤ 0.05 are displayed on the graphs. All bars and error bars represent mean ± SD.

**Fig. 6.**
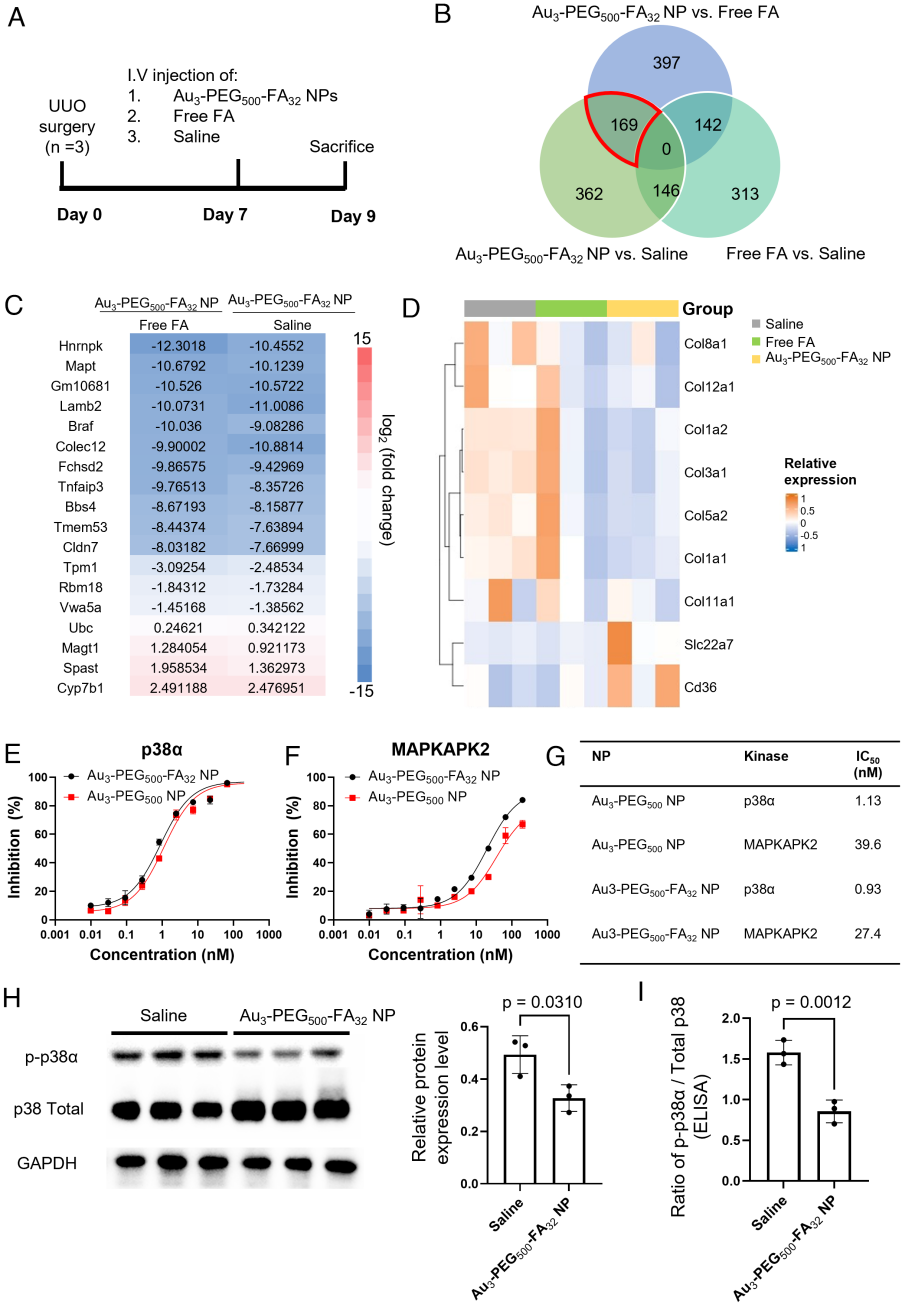
Au_3_-PEG_500_-FA_32_ NP inhibited ECM-related genes and p-p38α in the UUO kidney. (*A*) UUO mice were treated with a single i.v. dose of Au_3_-PEG_500_-FA_32_ NP, saline, or free FA on day 7 post-UUO surgery and then killed on day 9. (*B*) Venn diagram of differentially expressed genes (DEGs) identified in pairwise comparisons between the three treatment groups (Q < 0.05). Numbers in the overlapping region between two or more circles indicate the number of DEGs shared between the pairwise comparisons. Note that “Group X vs. Group Y” shows statistically significant changes in RNA expression that were found in Group X with reference to Group Y as baseline. There are 169 common DEGs in the “Au_3_-PEG_500_-FA_32_ NP vs. saline” and “Au_3_-PEG_500_-FA_32_ NP vs. free FA” pairwise comparisons (red border in Venn diagram). (*C*) Of the 169 common DEGs, 18 of them have FPKM values > 2 for the UUO kidneys analyzed by RNA-seq. (*D*) The heatmap shows that Au_3_-PEG_500_-FA_32_ NP treatment down-regulated genes related to collagen and up-regulated genes related to vesicle transport when compared to free FA and saline treatments. (*E* and *F*) Percent inhibition of p38α and MAPKAPK2 by Au_3_-PEG_500_-FA_32_ (black) and Au_3_-PEG_500_ NP (red) using the Z-Lyte assay. (*G*) IC_50_ values of Au_3_-PEG_500_-FA_32_ and Au_3_-PEG_500_-FA_32_ NPs. (*H*) Western blot analysis revealed significant inhibition of p-p38α upon treatment with Au_3_-PEG_500_-FA_32_ NP when compared to saline. Data are from n = 3, across one experiment. (*I*) ELISA analysis of the UUO kidney revealed significant inhibition of p-p38α upon treatment with Au_3_-PEG_500_-FA_32_ NP when compared to saline. Data are from n = 3, across one experiment. In (*H*) and (*I*), statistical significance was evaluated using the Mann–Whitney *U* test. All *P* values ≤ 0.05 are displayed on the graphs. All bars and error bars represent mean ± SD.

Initially, we explored the effect of NP size on delivery to the kidney by injecting Au_x_-PEG_y_ NPs into UUO mice on day 7 post-UUO surgery and killed the animals 24 h postinjection. For all NP sizes, our chosen dose was a constant mass of 100 µg Au (or 10^12^ to 10^14^ NPs depending on NP size) per mouse, high enough to overcome any variation in organ retention due to NP dose (25). Seven days is the minimal time required for renal fibrosis to be established (26). By quantifying the gold contents in the kidneys by inductively coupled plasma–mass spectrometry (ICP-MS), we detected at the organ level that the smallest Au_2_-PEG_200_ NP accumulated most abundantly in both fibrotic UUO (1.8 %ID) and nonfibrotic contralateral (CL) (2.8 %ID) kidneys of the five NP sizes studied (Fig. 1*B* and *SI Appendix*, Fig. S9). The accumulation of Au_3_-PEG_500_, Au_5_-PEG_1000_, Au_5_-PEG_5000_, and Au_20_-PEG_5000_ NPs in the UUO kidneys was similar (~1 %ID). At the tissue level, silver enhancement staining of the UUO kidney sections reveals that the smaller Au_2_-PEG_200_ and Au_3_-PEG_500_ NPs (<10 nm) could be found in the tubule lumen, but the larger Au_5_-PEG_1000_, Au_5_-PEG_5000_, and Au_20_-PEG_5000_ NPs (>10 nm) were only localized in peritubular capillaries (Fig. 1 *C* and *D*). Kidney tissues of uninjected mice had no silver stain. These data suggest that Au_2_-PEG_200_ and Au_3_-PEG_500_ NPs have viable sizes to reach renal tubules. While Au_2_-PEG_200_ NP more abundantly accumulates in the UUO kidney and are renally cleared more efficiently than Au_3_-PEG_500_ NP (*SI Appendix*, Fig. S10), we excluded Au_2_-PEG_200_ NP from further investigations because it does not solubilize FA in water (*SI Appendix*, Fig. S11). FA is poorly soluble in water under neutral conditions (27), and PEG_200_ is too short to solubilize FA (441 Da). Also, while the larger Au_5_-PEG_5000_ NP accumulated slightly more in the UUO kidney than Au_3_-PEG_500_ NP, it did not reach the renal tubules. Therefore, we concluded that Au_3_-PEG_500_ NP has the optimal size for loading FA and delivery to renal tubules. Subsequently, we only kept Au_3_-PEG_500_ NP (and its FA-conjugated version) to address their binding to FRs and delivery to tubules (Figs. 2 and 3), inhibition of renal fibrosis (Figs. 4 and 5), and inhibition of fibrosis-related genes and kinases (Fig. 6).

Next, we evaluated the effect of disease stage on delivery of Au_x_-PEG_y_ NPs to the UUO kidney on days 7, 14, or 28 post-UUO surgery. On day 7 postsurgery, dilatation of collecting ducts was evident, and the tubular epithelial cells were flattened and lost their cuboidal shape (*SI Appendix*, Fig. S12). On days 14 and 28 post-UUO surgery, less intact tubule segments and more deposition of ECM in the tubulointerstitial area were observed (*SI Appendix*, Fig. S13). As fibrosis progressed, the UUO kidney became lighter due to loss of tissue mass, tubular injury, and cell death; conversely, the CL nonfibrotic kidney becomes heavier in response to functional renal compensatory changes caused by UUO surgery (*SI Appendix*, Fig. S14). Our data showed that a later disease stage caused more severe fibrosis that disfavors NP delivery. Even when we normalized the amount of gold to %ID/g to eliminate any bias due to differences in tissue mass as a function of disease stage, we still observed much less accumulation of NPs in the UUO kidney of mice injected on day 28 than mice injected on day 7 for most NP sizes tested (Fig. 1*E*). The %ID in the UUO kidney on day 28 was nonzero, indicating accumulation of some NPs. We reasoned that fluid flow inside the tubule lumen does not vanish to zero upon UUO (19), consistent with the reported renal clearance of gold NPs in UUO mice (14). Also, we detected a similar negative correlation between disease stage and NP content in the liver (*SI Appendix*, Fig. S15), but there was no significant effect of disease stage on blood pharmacokinetics (*SI Appendix*, Fig. S16). More studies are required to decipher the effect of UUO surgery on bio–nano interactions outside of the kidney. From these data, we selected day 7 post-UUO surgery as the disease stage for our later studies to ensure adequate retention of NPs in the fibrotic kidney.

### In Vitro and Ex Vivo Binding of Au_3_-PEG_500_-FA_32_ NP to Renal Tubules.

Immunohistochemistry (IHC) analysis of kidney biopsies obtained from patients with varying degrees of renal fibrosis revealed that some fibrotic tubules had higher expression of FR than their adjacent healthy tissues (Fig. 2*A*). In the fibrotic kidney of UUO mice, we likewise detected that only a selected fraction of renal tubules overexpresses FR (Fig. 2*B*); such an expression pattern is distinct from the diffuse, homogenous expression of FR in the healthy and CL kidneys.

To prepare Au_3_-PEG_500_-FA_32_ NP, we added bifunctional PEG_500_ strands with a thiol group on one end and a FA molecule on the other end (HS-PEG_500_-FA) to an aqueous suspension of unmodified Au_3_ NPs (Fig. 2*C*). When compared to Au_3_-PEG_500_ NP, Au_3_-PEG_500_-FA_32_ NP is slightly smaller in terms of hydrodynamic size (7.0 vs. 9.4 nm). Based on the method of PEG strand displacement, we detected fewer PEG strands in Au_3_-PEG_500_-FA_32_ NP than Au_3_-PEG_500_ NP (81 vs. 32 strands/NP), noting that PEG_500_-FA is bulkier than methoxy-PEG_500_. We corroborated this trend using TGA (*SI Appendix,* Table S3) and TEM imaging with negative staining (*SI Appendix,* Fig. S3). Au_3_-PEG_500_-FA_32_ NP remains stable in 50% FBS at 37 °C (*SI Appendix,* Fig. S6) and is not cytotoxic to mouse primary renal tubule cells (*SI Appendix*, Fig. S8). Importantly, Au_3_-PEG_500_-FA_32_ NP shares a similar profile of proteins bound as Au_3_-PEG_500_ NPs upon incubation in blood from UUO mice. Polyacrylamide gel analysis and bicinchoninic acid assay revealed similar amounts of blood proteins bound to Au_3_-PEG_500_ and Au_3_-PEG_500_-FA_32_ NP (~0.4 μg of proteins per cm^2^ of NP surface; *SI Appendix,* Fig. S17 *A* and *B*); this protein density is of the same order of magnitude to that derived from the adsorption of antibodies to 4-nm gold NPs (28). Liquid chromatography–tandem mass spectrometry (LC-MS/MS) analysis showed that the most abundant serum proteins bound to both NP types, which account for ~60% of total proteins bound, were albumin, apolipoprotein-AI, transferrin, and immunoglobulin (*SI Appendix*, Fig. S17*C*). These data suggest that any difference in interactions of both NP types in the kidney is unlikely due to a difference in serum proteins bound. Next, ICP-MS measurements revealed 3.5-fold higher association of Au_3_-PEG_500_-FA_32_ NP with primary renal tubule cells than Au_3_-PEG_500_ NP in vitro (*SI Appendix*, Fig. S18). Confocal images of primary tubule cells treated with Cyanine (Cy) 5.5-labeled NPs verified more abundant cellular entry of Au_3_-PEG_500_-FA_32_ NP and stronger association with FRs than Au_3_-PEG_500_ NP, as evidenced by a higher Pearson’s correlation coefficient of the former (Fig. 2*D*). Further, Au_3_-PEG_500_-FA_32_ NP bound to UUO kidney tissues more abundantly than Au_3_-PEG_500_ NP ex vivo. When UUO kidney tissues were freshly diced to expose the tubule lumen and incubated with identical concentrations of NPs, ICP-MS measurements revealed 3.5-fold higher association of Au_3_-PEG_500_-FA_32_ NP with the tissues than Au_3_-PEG_500_ NP (Fig. 2*E*). As renal tubules occupy ~88% of the total kidney volume (12) and FR is mostly expressed on proximal tubules, these data suggest the binding of Au_3_-PEG_500_-FA_32_ NP to renal tubules.

It is challenging to verify whether Au_3_-PEG_500_-FA_32_ NP specifically binds to FRs on renal tubules by competitive binding assays in vivo, because intact immunoglobulin blocking antibodies are too large (>10 nm) to cross the GFB for accessing the renal tubules (29). Therefore, we sought to address its binding to FR on kidney tissues under serum-containing conditions ex vivo. Additionally, we included megalin, another receptor on proximal renal tubules for reabsorbing nutrients, to this ex vivo study because theoretical predictions made by the Search Tool for the Retrieval of Interacting Genes/Proteins (STRING) (30) showed that albumin, the top serum protein bound to Au_3_-PEG_500_-FA_32_ NP, is likely to interact with megalin (*SI Appendix*, Fig. S19). Pretreatment of freshly diced UUO kidney tissues with antibodies against FR or megalin both lowered the tissue association of Au_3_-PEG_500_-FA_32_ NPs, but the reduction achievable by blocking FR was greater (Fig. 2*F*). These data suggest that the bound serum proteins did not severely mask the binding of Au_3_-PEG_500_-FA_32_ NP to FRs on renal tubules.

### In Vivo Delivery of Au_3_-PEG_500_-FA_32_ NP to the Tubules of the Fibrotic Kidney.

To investigate whether Au_3_-PEG_500_-FA_32_ NP can be localized to renal tubules in vivo, we i.v. injected UUO mice with the NPs and examined their accumulation in the kidneys 24 h postinjection. Silver enhancement staining of UUO kidney sections shows that Au_3_-PEG_500_-FA_32_ NPs mainly accumulated in some tubule cells or were found inside the tubule lumen (Fig. 3 *A* and *B*), while Au_3_-PEG_500_ NPs were mainly found in the tubule lumen or interstitial space (*SI Appendix*, Figs. S20 and S21). These images suggest that the NPs were flowing through the tubules after glomerular filtration at animal killing. Importantly, the distribution pattern of silver-stained Au_3_-PEG_500_-FA_32_ NPs in selected renal tubules of the UUO kidney is consistent with the distribution pattern of FR overexpression on selected renal tubules in Fig. 3*C*. ICP-MS data showed that significantly more Au_3_-PEG_500_-FA_32_ NPs (3.6 %ID) accumulated in the UUO kidney than Au_3_-PEG_500_ NPs (1.0 %ID), while similar amounts of Au_3_-PEG_500_ NPs (0.8 %ID) and Au_3_-PEG_500_-FA_32_ NPs (0.7 %ID) were seen in the CL kidney (Fig. 3*D*). We also detected gold in the urine samples for both NP types, suggesting their renal clearance (*SI Appendix*, Fig. S10). To verify the selective delivery to the fibrotic kidney, we showed that under the same experimental conditions, only 1.0 %ID of Au_3_-PEG_500_-FA_32_ NPs accumulated in each kidney of healthy Balb/c mice (*SI Appendix*, Fig. S22). Next, we investigated whether the overexpression of FR by some tubules contributes to the increased uptake of Au_3_-PEG_500_-FA_32_ NPs by the tubules in the UUO kidneys. FRs in the UUO kidney section were stained by IHC and Au cores were imaged by confocal reflectance microscopy. Notably, the Au reflectance signal and FRs on the tubules were localized only for the Au_3_-PEG_500_-FA_32_ NP sample but not the Au_3_-PEG_500_ NP sample (Fig. 3*E*), demonstrating that Au_3_-PEG_500_-FA NPs bound to FRs on tubules. TEM imaging with energy dispersive X-ray spectroscopy confirmed the localization of Au_3_-PEG_500_-FA_32_ NPs inside tubule cells (Fig. 3*F* and *SI Appendix*, Fig. S23). A representative enlarged TEM image portrays individual Au_3_-PEG_500_-FA_32_ NPs in the tubule, suggesting their colloidal stability in vivo. If these NPs were to become aggregated after injection into the bloodstream, we reasoned that they would become too large to cross the GFB and enter the renal tubules. Further, to quantify the tissue-level distribution of the NPs, we separated the glomeruli, tubules, and tubulointerstitial space from the UUO and CL kidneys (*SI Appendix*, Figs. S24 and S25) and measured their respective Au contents using ICP-MS. While significantly more Au_3_-PEG_500_-FA_32_ NPs accumulated in the tubules and tubulointerstitial cells of the UUO kidney (1.0 %ID) than the CL kidney (0.2 %ID), the accumulation of Au_3_-PEG_500_ NPs in both UUO (0.4 %ID) and CL (0.3 %ID) kidneys were similar (Fig. 3*G*). Significantly more Au_3_-PEG_500_-FA_32_ NPs (1.5 %ID) than Au_3_-PEG_500_ NPs (0.8 %ID) were detected in the interstitial space of the UUO kidney, matching the enhanced uptake of Au_3_-PEG_500_-FA_32_ NPs by renal tubules and suggesting transcytosis of NPs from renal tubules to interstitial space. Neither Au_3_-PEG_500_ nor Au_3_-PEG_500_-FA_32_ NPs accumulated significantly in the glomeruli of UUO and CL kidneys, and accumulation in the CL kidneys was similar for both NP types (Fig. 3*G*). Last, we prepared Au_3_-PEG_500_-FA NPs with different FA loadings (z) by adding different ratios of methoxy-terminated thiolated PEG and FA-terminated thiolated PEG to citrate-capped Au_3_ NPs. These Au_3_-PEG_500_-FA_z_ NPs have similar hydrodynamic diameters of ~7 nm and are stable in 50% FBS. Notably, NP accumulation in the UUO kidney positively correlated with FA loading (Fig. 3*H*); the accumulation of Au_3_-PEG_500_, Au_3_-PEG_500_-FA_8_, Au_3_-PEG_500_-FA_16_, and Au_3_-PEG_500_-FA_32_ NPs in the UUO kidney were 1.0 %ID, 1.2 %ID, 2.1 %ID, and 3.6 %ID, respectively. No such correlation was seen with the CL kidney. Taken together, our data demonstrate that Au_3_-PEG_500_-FA_32_ NPs showed enhanced delivery to the tubules of the fibrotic kidney.

Because FA is negatively charged and negatively charged polymers can accumulate in the kidney (31), we explored the potential confounding effect of surface charge on delivery to the kidney. We injected Au_3_-PEG_500_-COOH NPs that contain a carboxylate group in the distal end of each PEG chain; this NP has a similar size to Au_3_-PEG_500_-FA_32_ NP but a more negative surface charge (−13.5 vs. −23.7 mV). The accumulation of Au_3_-PEG_500_-COOH NPs in the UUO kidney (1.9 %ID) was higher than that of Au_3_-PEG_500_ NPs (1.0 %ID) but lower than that of Au_3_-PEG_500_-FA_32_ NPs (3.6% ID), suggesting that negative surface charge alone does not primarily account for the superior delivery of Au_3_-PEG_500_-FA_32_ NPs to the UUO kidney (*SI Appendix*, Figs. S26 and S27). Finally, it is challenging to experimentally pinpoint the exact route of delivery of NPs to the UUO kidney. As Au_3_-PEG_500_-FA_32_ and Au_3_-PEG_500_ NPs are similarly sized to the size cutoff of soft biomolecules passing through the GFB [~10 nm; (32)] and the pore size of peritubular capillaries (33), they may reach the interstitial space either via transcytosis from renal tubules or via extravasation from peritubular capillaries. Still, we reasoned that Au_3_-PEG_500_-FA_32_ NP most likely crosses the GFB to enter renal tubules and later the interstitial space because it can bind to FRs on renal tubules and FR is mostly found on the apical side of proximal tubules (34).

### In Vivo Reduction of Kidney Degeneration and Renal Fibrosis by Au_3_-PEG_500_-FA_32_ NP.

For our efficacy studies, we i.v. injected a single dose of NP (2.5 mg-Au/kg-mouse) to UUO mice with established renal fibrosis (day 7 post-UUO surgery). Seven days later (day 14) when severe renal fibrosis is expected, the animals were killed. Controls included Au_3_-PEG_500_ NP without FA (2.5 mg-Au/kg-mouse), free FA without gold (0.12 mg-FA/kg-mouse; same FA dosage as Au_3_-PEG_500_-FA_32_ NP), saline, and Captopril [5 mg-drug/kg-mouse/day (35)] (Fig. 4*A*). Our FA dosage is ~2,000 times lower than the dosage that can induce kidney injury by forming FA crystals in the tissue (36), so we do not expect toxicity induced by FA in our treatment. To validate this claim, we histologically examined the major internal organs and performed hematology tests to show that Au_3_-PEG_500_-FA_32_ NP was nontoxic up to 7 d postinjection into UUO mice (*SI Appendix*, Figs. S28–S31). IHC data confirmed that Au_3_-PEG_500_-FA_32_ NP did not damage the CL kidney (*SI Appendix*, Fig. S32), and serum creatinine levels remained in the normal range (37) as UUO mice still have a functioning CL kidney (*SI Appendix*, Fig. S33). We verified that the endotoxin levels of Au_3_-PEG_500_ and Au_3_-PEG_500_-FA_32_ NP (0.01 and 0.003 EU/mL, respectively; *SI Appendix*, Table S5) were lower than the upper limit stipulated for preclinical research (1 EU/mL for injecting into a 20-g mouse over a 24-h time window) (38).

Gross comparison of kidneys from healthy mice and CL kidneys from UUO mice (Fig. 4*B*) with UUO kidneys showed that only Au_3_-PEG_500_-FA_32_ NP rescued the UUO kidney from tissue degeneration, but Au_3_-PEG_500_ NP, free FA, Captopril, or saline did not (Fig. 4*C*). Corroborating histological images revealed that only Au_3_-PEG_500_-FA_32_ NP significantly reduced the fraction of injured renal tubules (as evidenced by dilated tubules and loss of cytoplasm) when compared to saline (58%; Fig. 4 *D* and *E*). Tissue degeneration was quantified by measuring the UUO kidney weight of each mouse and normalizing it to its body weight (Fig. 4*F*). Mice treated with Au_3_-PEG_500_-FA_32_ NP had a significantly higher kidney-to-body weight ratio than those treated with saline or Au_3_-PEG_500_ NP, but we cannot exclude any potential contribution of edema to the increased kidney weight.

We evaluated whether Au_3_-PEG_500_-FA_32_ NP can treat renal fibrosis. The primary treatment outcome is the level of type I collagen, the main component of fibrous scar (1). IHC analysis showed that Au_3_-PEG_500_-FA_32_ NP significantly reduced the (5.0%) fractional area of type I collagen in the UUO kidney when compared with saline (13.1%), but Au_3_-PEG_500_ NP (11.7%), Captopril (13.5%), and free FA (11.2%) did not (Fig. 5 *A* and *B*). Here, Captopril did not treat renal fibrosis probably because, like Au_3_-PEG_500_-FA_32_ NP, we injected Captopril after the establishment of fibrosis; past evidence of the antifibrotic effect of Captopril was preventive and based on simultaneous Captopril administration and fibrosis development (39). western blot data confirmed that Au_3_-PEG_500_-FA_32_ NP attenuated the type I collagen content in the UUO kidney by ~10-fold when compared to saline (Fig. 5*C*). Next, we assessed the expression level of α-smooth muscle actin (α-SMA) in the UUO kidney. α-SMA^+^ cells, such as myofibroblasts, are the primary source of ECM in fibrotic kidneys (40). Like collagen, α-SMA lies downstream of the p38 MAPK signaling pathway (41). IHC showed that the area with positive α-SMA was significantly reduced only for Au_3_-PEG_500_-FA_32_ NP (8.8%) when compared with saline (14.3%) (Fig. 5 *D* and *F*). We then evaluated the population of CD3^+^ T cells in the UUO kidney because T cell infiltration is a pathological feature of renal fibrosis (42). Au_3_-PEG_500_-FA_32_ NP showed the most drastic reduction in the population of CD3^+^ T cells (7 cells/frame) when compared to saline (21 cells/frame), while the levels of reduction by Au_3_-PEG_500_ NP (15 cells/frame), Captopril (16 cells/frame), or free FA (13 cells/frame) were less notable (Fig. 5 *E* and *G*). These data support the antifibrotic efficacy of Au_3_-PEG_500_-FA_32_ NP.

We believe that the therapeutic efficacy of Au_3_-PEG_500_-FA_32_ NP primarily stems from the gold core for two reasons. To start with, past clinical studies reported a therapeutic effect of FA for CKD, but the data were inconsistent across different trials (43–45). Moreover, we compared the efficacy of Au_3_-PEG_500_ NP injected at a ~3.5-fold higher dose (~8.8 mg-Au/kg-mouse) to that of Au_3_-PEG_500_-FA_32_ NP injected at the original dose. This experiment serves to compensate for the less efficient delivery of Au_3_-PEG_500_ NP to the UUO kidney than Au_3_-PEG_500_-FA_32_ NP (1.0% vs. 3.6% ID; Fig. 3*D*) and to ensure a similar absolute mass of Au to be delivered for both NP types, as confirmed by ICP-MS (*SI Appendix*, Fig. S34). Notably, a 3.5-fold higher dose of Au_3_-PEG_500_ NP reduced tissue degeneration and fibrosis at a similar efficacy as Au_3_-PEG_500_-FA_32_ NP at the original dose (*SI Appendix*, Figs. S35 and S36), so we surmise that Au_3_-PEG_500_ NP injected at the original dose did not reach the therapeutic window. Thus, attachment of FAs enhanced the delivery and efficacy of the gold cores without requiring more gold to be injected.

### Mechanism for the Antifibrotic Efficacy of Au_3_-PEG_500_-FA_32_ NP.

We performed RNA sequencing (RNA-seq) analysis to elucidate the changes in RNA expression in the UUO kidney 2 d postinjection of Au_3_-PEG_500_-FA_32_ NP, free FA, or saline (Fig. 6*A*). This earlier time point than our efficacy study serves to avoid any degradation or translation of mRNA. RNA-seq analysis reveals <800 differentially expressed genes (DEGs) that were enriched in the pairwise comparison between Au_3_-PEG_500_-FA_32_ NP and saline (Au_3_-PEG_500_-FA_32_ NP vs. saline) and the pairwise comparison between Au_3_-PEG_500_-FA_32_ NP and free FA (Au_3_-PEG_500_-FA_32_ NP vs. free FA), suggesting that Au_3_-PEG_500_-FA_32_ NP treatment did not cause pronounced off-target gene regulation (Fig. 6*B*). Of the 169 overlapping genes found in both pairwise comparisons, only 18 out of them have values of fragments per kilobase of transcript per million mapped reads (FPKM) >2 in the UUO kidneys (Fig. 6*C*); see detailed explanation in **SI Appendix*, Materials and Methods*). The gene ontology (GO) terms associated with these 18 genes include collagen-containing ECM (Colec12 and Vwa5a), extracellular space (Lamb2 and Tpm1), and apical membrane (Cldn7). Notably, the type I collagen (Col1a1) gene was significantly down-regulated in the Au_3_-PEG_500_-FA_32_ NP group when compared to the saline and free FA groups (Fig. 6*D* and *SI Appendix,* Table S6). IHC staining of the UUO kidneys showed Au_3_-PEG_500_-FA_32_ NP group had a lower fractional area of type I collagen than the free FA or saline groups 2 d postinjection (*SI Appendix*, Figs. S37 and S38), validating our RNA-seq data. These data suggest that Au_3_-PEG_500_-FA_32_ NPs suppressed ECM-associated genes more effectively than free FA or saline and underscore the therapeutic role of the gold core. Further, both pairwise comparisons yielded enriched GO terms of “apicolateral plasma membrane” and “brush border membrane” (*SI Appendix*, Figs. S39–S41), and the genes involved were Slc22a7 (a transporter of organic anions) and CD36 (which can bind to negatively charged ligands) (Fig. 6*D*). This result reinforces our postulate that anionic Au_3_-PEG_500_-FA_32_ NP enters renal tubules from the apical side (tubule lumen) after crossing the GFB.

Last, we conducted a kinome screen to identify kinases whose activities were inhibited by Au_3_-PEG_500_-FA_32_ NP. Of the 280 kinases tested, an NP concentration of 200 nM inhibited 27 kinases by >80% (*SI Appendix*, Table S7); a few of them were relevant to CKD, including p38α, its downstream MAPK-activated protein kinase 2 (MAPKAPK2) (46), G-protein-coupled receptor kinase 4 [GRK4; which is up-regulated after kidney injury (47)] and ribosomal S6 kinase 2 [RSK2; which is linked to survival of immune cells in the fibrotic kidney (48)] (*SI Appendix*, Fig. S42). The half-maximal inhibitory concentrations (IC_50_’s) of Au_3_-PEG_500_-FA_32_ NP for p38α and MAPKAPK2 were 0.9 nM and 27.4 nM, respectively, indicating high potency of the NP (Fig. 6 *E*–*G*). The IC_50_’s of Au_3_-PEG_500_ NP for both kinases were similar to those of Au_3_-PEG_500_-FA_32_ NP, implying that inhibited p38α activity stems from the gold core. As the linkage between p38α and CKD is the most reported of all CKD-relevant kinases identified, we further proved that Au_3_-PEG_500_-FA_32_ NP treatment inhibited phosphorylated p38α (p-p38α) in the UUO kidney using western blot and enzyme-linked immunosorbent assay (ELISA) (Fig. 6 *H* and *I*). This result validates the kinome profiling data and highlights the self-therapeutic mechanism of action of the 3-nm gold NP.

## Discussion

We have presented a design [Au_3_-PEG_500_-FA_32_ NP (~7 nm)] to transport i.v. injected NPs into renal tubules, where tubulointerstitial fibrosis takes place. An NP size <10 nm supports delivery to renal tubules and FA conjugation supports binding to FRs on tubules. Past studies reported the use of larger ≥20-nm gold NPs for alleviating diabetic nephropathy, another type of CKD (49, 50) that stems from fibrosis in the glomerulus, but they are unsuitable for treating tubulointerstitial fibrosis because they are too large to reach the tubules. In contrast to previous studies on the organ-level distribution of sub-10-nm NPs to kidneys (~6 µg-gold/g-tissue) (51), this work yields insights into their tissue- and cellular-level transport to renal tubules, an emerging trend in kidney nanomedicine (52). The overexpression of FRs was only found on selected tubules in the fibrotic kidney, but it suffices to boost the retention of Au_3_-PEG_500_-FA_32_ NPs in the fibrotic kidney up to 3.6% ID (or 28.8 µg-gold/g-tissue), a pronounced improvement in delivery. FRs are expressed at very low levels in most tissues, except for placenta for embryonic development, kidney for folate resorption, and tumor to meet the folate demand of rapidly dividing cells (53). While we believe that FA acts primarily as a targeting ligand for FRs to facilitate endocytosis by renal tubules (54), it is unclear whether FA binding to fibrotic renal tubules contributes to antifibrotic efficacy. The exact signaling pathways of FRs are not fully understood, but there are some potential candidates (55). Binding of FA to FRs can activate the extracellular signal-regulated kinase (ERK) (56) and Janus kinase (JAK)-signal transducer and activator of transcription protein (STAT) pathways (57) that are related to the growth, differentiation, and survival of cancer cells. Overall, this work not only improves our understanding in kidney–NP interactions but also showcases the rational design of NPs based on disease pathophysiology (58). Still, the applicability of our findings to other types of CKD remains to be addressed. The UUO model entails the retardation of fluid flow inside blocked tubules (59) and overexpression of FRs on selected renal tubules, but both attractive features for cellular entry may not apply to other forms of CKD.

This work highlights the treatment potential of self-therapeutic gold nanomedicines for tubulointerstitial fibrosis. It lends credence to an emerging notion that gold NPs are self-therapeutic for a broad spectrum of diseases, such as cancer (60) and skin inflammation (61). Our treatment involved only one injection 7 d post-UUO surgery when fibrosis is established, in contrast to many existing preventive strategies that entailed multiple injections of drugs (62) or drug-loaded NPs (*SI Appendix*, Table S1*A*) before fibrosis was established. Chitosan NPs complexed with siRNA against an inflammatory enzyme required injection 3 d prior to UUO surgery (9). Albumin NPs loaded with small molecules that block p38 signaling entailed injection 2 d post-UUO surgery (63). This point is of clinical importance; as early development of CKD is asymptomatic, patients are mostly diagnosed at a later disease stage. How 3-nm gold NPs mechanistically inhibit p38α and other CKD-related kinases will require further elucidation.

On clinical translation, the simple design and facile synthesis of Au_3_-PEG_500_-FA_32_ NP will be conducive to mass production. As PEG is a U.S. Food and Drug Administration (FDA)-cleared polymer used for drug delivery and FA is a common vitamin-based health supplement, the only potential regulatory hurdle will lie on gold NP. Concerns over gold-based therapies exist because gold, a heavy metal, may accumulate in tissues in the long run and past treatments that contained gold salts caused side effects (64). Currently, gold NPs that facilitate the cellular delivery of biomolecules are under clinical tests. A phase 0 trial on patients with glioblastoma showed no severe toxicity upon i.v. infusion of RNA-conjugated gold NPs at microdose levels (65), a reassuring result for gold NP-based therapies. Yet, because this RNA-conjugated gold NP remained in patient tumors that recurred up to 174 days posttrial enrollment, chrysiasis and other adverse events linked to long-term accumulation (66) and biotransformation will need to be evaluated in humans. Here, there are two advantages of a 3-nm gold core. The Au_3_-PEG_500_-FA_32_ NP can be cleared via renal and hepatobiliary pathways (*SI Appendix*, Fig. S10), and intracellular degradation of 3-nm gold NPs will be faster and require the generation of less reactive oxygen species than larger gold NPs (67).

## Methods

For detailed Materials and Methods, please see *SI Appendix*.

### Animals.

All procedures followed the guidelines stipulated by the Animal Experimentation Ethics Committee at The Chinese University of Hong Kong (CUHK). Male Balb/c mice between 8 and 12 wk of age were used and randomly divided into various treatment groups. All mice were housed in a temperature- and humidity-controlled environment with a 12-h light/dark cycle. For all distribution and efficacy studies, NPs or free FA were formulated in 100 µL of 5% dextrose (D5W; Sigma) for a single intravenous (i.v.) injection using a 29-gauge insulin syringe (Terumo). For all biodistribution and efficacy studies, the sample size (n) indicates biological replicates.

### UUO.

UUO surgery was performed as previously described (21). Mice were anesthetized by an intraperitoneal injection of ketamine (100 mg/kg; Alfasan International B.V.) and xylazine (10 mg/kg; Alfasan International). The peritoneum was cut along the midline, and the left ureter was isolated and ligated twice using a 5-0 suture (NingBo Cheng-He Microsurgical Instruments). The bowel was laid back and the peritoneum was closed with suture. The mice were placed under a heating lamp to maintain body temperature until they recovered from anesthesia. For analgesia, three injections of buprenorphine (Temgesic, 0.05 mg/kg) were given to the mice subcutaneously every 12 h postsurgery. To verify the development of fibrosis, mice were killed by cervical dislocation with anesthesia, and kidneys were weighted and processed for histological analysis.

### Sample Size Calculation for Efficacy Studies.

We used Dunnett’s test to deduce the required size of each treatment group (*N*) (68). Dunnett’s test is a multiple comparison procedure that compares the efficacy of each treatment group with the same control group. Here, we tested “H0: All treatment groups are equivalent to the control group” against “H1: There exists one group that is superior to the control group.” We compared the treatment groups and the control group in a way that i) the chance of committing type 1 error is <5% and that ii) our comparison is of power 80%. Dunnett’s formalism states that *p* = √*N*δ/σ, where *p* is the correlation coefficient that depends on *N*. There are four treatment groups and a control group (i.e., saline), so *p* is 4.46 (56). If the superior treatment group gives an outcome (*δ*) of 1.5 SD (*σ*) better than the control group, the required *N* is (4.46/1.5)^2^ = 8.84 ≈ 9.

### Efficacy.

A single dose of Au_3_-PEG_500_ NP, Au_3_-PEG_500_-FA NP, free FA (formulated in 0.1 mL of D5W), or saline was injected i.v. into UUO mice with established renal fibrosis (7 d post-UUO surgery) via the tail vein. FA was dissolved in sterile BioPerformance Certified dimethyl sulfoxide (DMSO; Sigma) and formulated in D5W prior to injection. The final concentration of DMSO was <1%. Au_3_-PEG_500_-FA NPs formulated in D5W was injected i.v. at a dose of 0.12 mg-FA/kg-mouse (or equivalently 2.5 mg-Au/kg-mouse). Au_3_-PEG_500_ NPs were injected i.v. as a vehicle only control at a dosage of 2.5 mg-Au/kg-mouse. Saline was injected i.v. as a negative control. Captopril (Tokyo Chemical Industry) was injected intraperitoneally (i.p) at a dose of 5 mg/kg daily beginning on day 7 post-UUO surgery for 7 d (30). Mice in all groups were killed on day 14 post-UUO surgery (7 d posttreatment), with n = 9 per group. All treated mice were anesthetized and killed by cervical dislocation 7 d posttreatment. For blood collection, mice were anesthetized, and blood was collected via cardiac puncture into plain tubes for serum collection. The kidneys were dissected, and the ureters were removed before weighing. The remaining kidney tissue was fixed in 10% buffered formalin for histological analysis.

### Data Processing and Analysis.

The Prism (GraphPad Software), Excel software, and SPSS software were used for data analysis and graph construction. Comparison of equivalence between multiple treatment groups and the untreated control group was computed by Dunnett’s test and one-way ANOVA using the SPSS software. All results are biological replicates (unless specified). For ANOVA analysis, Tukey’s post hoc test (with 95% confidence level) was used for multiple comparisons when the result was significant (*P* < 0.05). Normality of sampling distribution of means was validated by the Kolmogorov–Smirnov test. There are no outliers outside 3 SD. Homogeneity of variance was validated by Levene’s test.

## Supplementary Material

Appendix 01 (PDF)

## Data Availability

All study data are included in the article and/or *SI Appendix*.
