# Supplementary material for: A sub-10-nm, folic acid-conjugated gold nanoparticle as self-therapeutic treatment of tubulointerstitial fibrosis
Source: Proc Natl Acad Sci U S A. 2023 Oct 9;120(42):e2305662120. doi: 10.1073/pnas.2305662120 (PMC10589645; doi:10.1073/pnas.2305662120)
Supplement: Supplementary file 1 — Appendix 01 (PDF) [file pnas.2305662120.sapp.pdf]

Supplementary Information for

A sub-10 nm, folic acid-conjugated gold nanoparticle as self-therapeutic treatment of tubulointerstitial fibrosis

Cecilia Ka Wing Chan†, Cheuk Chun Szeto‡, Leo Kit Cheung Lee§, Yu Xiao §, Bohan Yin§, Xiaofan Ding†, Thomas Wai Yip Lee₸, James Yun Wong Lau†, Chung Hang Jonathan Choi§**

†Department of Surgery, §Department of Biomedical Engineering, ‡Department of Medicine & Therapeutics, ₸School of Pharmacy, The Chinese University of Hong Kong, Shatin, New Territories, Hong Kong.

*James Yun Wong Lau, *Chung Hang Jonathan Choi
Email: laujyw@surgery.cuhk.edu.hk, jchchoi@cuhk.edu.hk

This PDF file includes:

Supplementary text (SI Materials and Methods and SI Extended Results and Discussion)
Fig. S1 to S42
Tables S1 to S7
SI References

SI Materials and Methods

Preparation of Au_x-PEG_y NPs

Au_2-PEG_{200} NPs

Polyethylene glycol (PEG)-coated AuNPs of 2 nm in core diameter were synthesized as described previously (1). Tetrachloroauric acid ($HAuCl_4$; 0.02 mmol) and methoxy- and thiol-terminated PEG ($mPEG_{200}$-SH; 0.06 mmol, BiochemPEG) were dissolved in 7 mL of methanol/acetic acid 6:1 (v/v). Sodium borohydride (0.4 mmol, Sigma) in 2 mL of Nanopure water was added dropwise with rapid stirring. After 2 h of continuous stirring, a dark brown solution was formed, and the solvent was removed under vacuum at 40 °C using a rotary evaporator (R215, Buchi). The residues were dissolved in 5 mL of water. Then, the mixture was transferred to a 5–8 kDa molecular weight cut off membrane and dialyzed against Nanopure water for a minimum of three times over 72 h at room temperature. H

Au_3-PEG_{500}, Au_5-PEG_{1000}, and Au_5-PEG_{5000} NPs

3 nm AuNPs were synthesized as described previously (2). 150 mL of freshly prepared sodium citrate (2.2 mM) containing 0.1 mL of tannic acid (2.5 mM, Sigma) and 1 mL of potassium carbonate (K_2CO_3, 150 mM) was heated in a three-necked round bottom flask under vigorous stirring. When the temperature reached 70 °C, 1 mL of $HAuCl_4$ (25 mM) was injected and the reaction mixture was stirred for 5 min. The resultant 3 nm AuNP solution was stored at 4 °C. To synthesize 5 nm AuNPs, the product solution of 3 nm AuNPs was diluted by removing 55 mL of Au seeds and adding 55 mL of sodium citrate (2.2 mM). When the temperature of the solution reached 70 °C, two injections of 0.5 mL of $HAuCl_4$ (25 mM) were added at a time interval of 10 min. After 10 min, the reaction was completed, and the resultant 5 nm AuNP solution was stored at 4 °C.

Unmodified Au_x NPs (x = 3 or 5 nm) were functionalized with thiolated PEG ($mPEG_{500}$-SH, BiochemPEG; $mPEG_{1000}$-SH and $mPEG_{5000}$-SH, JenKem Technology) at a concentration of 10 PEG molecules per nm^2 of NP surface under stirring for 2 h. To remove the free PEG strands, the NPs were washed three times by centrifugation at 6000 rpm for 6 min, using centrifugal filters with a membrane size cutoff of 50 kDa (Merck Millipore).

Au_{20}-PEG_{5000} NPs

The synthesis protocol follows a published seed-mediated growth method (3). For the AuNP seed, 150 mL of freshly prepared sodium citrate (2.2 mM, Alfa Aesar) was heated in a three-necked round-bottomed flask under vigorous stirring. Upon boiling, 1 mL of $HAuCl_4$ (25 mM, Sigma) was injected, resulting in seed NPs of ~8 nm in size. Immediately afterward, the reaction was cooled until the temperature of the NP solution reached 90 °C. Then, 1 mL of $HAuCl_4$ solution (25 mM) was injected. After 30 min, this process of cooling down and injection of $HAuCl_4$ solution (25 mM) was repeated twice. After that, 55 mL of the mixture was removed, and the remaining mixture was diluted by adding 53 mL of Nanopure water and 2 mL of sodium citrate (60 mM). The size of the AuNPs was monitored by using UV-vis spectroscopy (Agilent Cary 5000). The cycle of 1) dilution, 2) injection of sodium citrate, and 3) addition of three doses of $HAuCl_4$ solution was repeated until the UV-vis spectra of the resultant NPs exhibit a localised surface plasmon resonance (LSPR) peak at 523 nm for ~20 nm AuNPs.

Unmodified Au_{20} NPs were functionalized with $mPEG_{5000}$-SH at a concentration of 10 PEG molecules per nm^2 of NP surface under stirring for 2 h. Excess PEG was washed three times by centrifugation at 15,000 rpm for 30 min.

Preparation of Au_x-PEG_y-FA_z NPs

Au_2-PEG_{200}-FA NPs

To prepare folic acid (FA)-terminated, polyethylene glycol (PEG)-coated AuNPs of 2 nm in core diameter (Au_2-PEG_{200}-FA NPs), we followed the procedures for Au_2-PEG_{200} NPs, except for substituting $mPEG_{200}$-SH with FA-PEG_{200}-SH (BiochemPEG). PEG-coated AuNPs of 2 nm in size were synthesized as reported (1). Tetrachloroauric acid ($HAuCl_4$; 0.02 mmol) and FA-PEG_{200}-SH (0.06 mmol) were dissolved in 7 mL of methanol/acetic acid 6:1 (v/v). Sodium borohydride ($NaBH_4$; 0.4 mmol, Sigma) in 2 mL of Nanopure water was added dropwise with rapid stirring. After 2 h of stirring, a dark brown solution was formed, and the solvent was removed under vacuum at 40 °C using a rotary evaporator (R215, Buchi). The residues were dissolved in 5 mL of water. Then, the mixture was transferred to a 5–

8 kDa molecular weight cutoff membrane (Spectrum Laboratories) and dialyzed against Nanopure water for at least three times over 3 d at room temperature (RT). As the purified Au_2-PEG_{200}-FA NPs were insoluble in water (Supplementary Fig. S8), further characterization was not performed.

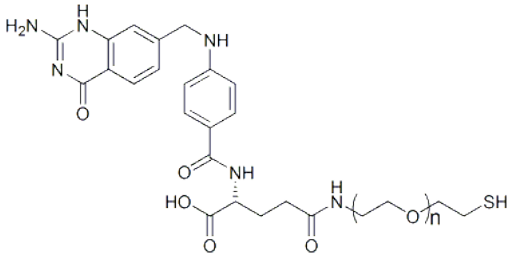

Chemical structure of FA-PEG-SH. (https://www.biochempeg.com/product/FA-PEG-SH.html)

Au_3-PEG_{500}-FA_z NPs

Unmodified Au_3 NPs were functionalized with different mixing ratios of FA-PEG_{500}-SH (Biochempeg) to mPEG_{500}-SH. Total PEG loading was kept constant at a concentration of 10 PEG molecules per nm^2 of NP surface. The mixing ratios of mPEG_{500}-SH and FA-PEG_{500}-SH are 3:1 for Au_3-PEG_{500}-FA_8 NPs (25% of the maximal amount of FA on the NP), 1:1 for Au_3-PEG_{500}-FA_{16} NPs (50% of the maximal amount of FA on the NP), and 0:1 for Au_3-PEG_{500}-FA_{32} NPs (100% of the maximal amount of FA on the NP). The exact loading of these of Au_3-PEG_{500}-FA_z NPs was verified by using the fluorophore-based method.

Physicochemical characterization of NPs

The concentration of Au_{20} NPs was determined by UV−vis-NIR spectrophotometry (Agilent Cary 5000) based on the Beer−Lambert's law. The molar extinction coefficient of 20 nm is 5.41 × 10^8 $M^{-1}cm^{-1}$ (4). The final concentration of AuNPs that are smaller than 5 nm (Au_2 NPs, Au_3 NPs, and Au_5 NPs) was determined by inductive coupled-plasma mass spectrometry (ICP-MS) (Agilent 7900) with reference to a standard curve of known gold concentration (Au 197 isotope) in parts per billion (ppb). To obtain an equivalent mass of gold in the NP solution for injection into animals and to quantify the gold contents in tissues and cells, we firstly converted the ICP-MS raw data from ppb to µg/L using the equation 1 ppb = 1 µg/L, followed by multiplying the volume of dilute 2% nitric acid that contains the digested tissues.

To characterize the morphology and measure the physical diameter of the entire PEG-coated gold NP (Au core + PEG), the NPs were negatively stained for TEM imaging. In brief, 10 µL of aqueous NP solution was dropped onto a plasma-treated (Harrick Plasma), formvar/carbon-coated copper grid (200 mesh; Beijing Zhongjingkeyi Technology) and left for 30 min. Then, the NP droplet was drawn off from the edge of the grid with filter paper. Next, EM Stainer solution (Nisshin-EM (5); catalog no.: 336) was diluted with Nanopure water by 4 times, and 10 µL of the diluted solution was added to each TEM grid for another 8 min. (EM Stainer is an electronic stain alternative to uranyl acetate.) After removing the EM Stainer solution, the grid was allowed to dry at RT for at least 4 h before visualization under TEM at a voltage of 100 kV (Hitachi H7700), at a magnification of 30,000–50,000. At higher magnifications, the PEG shell does not have enough contrast to be imaged, especially for Au_x-PEG_y NPs with shorter PEG strands (~500 Da). At lower magnifications, the entire Au_x-PEG_y NP appears too small to be imaged. Then, the length measuring function of the imaging software installed on-site at the Hitachi (H7700) TEM instrument was used to obtain an initial measurement of physical size. After this confirmatory step, more TEM images of a given NP sample were taken and analyzed using the Fiji image processing program as part of the ImageJ software. The physical diameters of the gold core and the PEG-coated gold NP were measured by manually drawing a straight line through the center of the NP from one end of the stained PEG shell circumference to another. Measurements were taken only where staining boundaries were clearly defined. At least 500 NPs for each NP type were counted.

The hydrodynamic diameters and zeta potential of NPs were measured by the DelsaMax PRO dynamic light scattering (DLS) analyzer (Beckman Coulter). Reported DLS data represent the values from three independent measurements. The DLS data quality was interpreted by analyzing the autocorrelation function and its fit, where the sum of square (SOS) threshold was set to be under 100. (Only measurements with an SOS value <100 were counted). As independent samples were used for different assays or experiments, a slight batch-to-batch variation was observed. To test colloidal stability, the NP solutions were mixed with equal volumes of 1) phosphate-buffered saline (PBS) (0.01 M), 2) 10% fetal

bovine serum (FBS) in PBS, 3) 50% FBS in PBS, and incubated at 37°C. After 24 h of incubation, the AuNP solutions were analyzed using UV-vis spectrometry and DLS. Reported DLS values represent mean ± SD from three independent measurements.

Quantification of PEG loading on the gold NP

Method I: Direct displacement of thiolated PEG strands by dithiothreitol (DTT)

For Au_x-PEG_y NPs:

The loading of PEG strands on the Au core was quantified using a fluorophore-based method (6), starting with the preparation of FAM-conjugated Au_x-PEG_y NPs. For all sizes, we chose a small mole ratio of 1:49 for FAM-PEG-SH to unlabeled mPEG-SH to achieve adequate fluorescence for detection by fluorimetry while excluding the scenario in which excess FAM-PEG may (i) cause interparticle aggregation due to hydrophobic interaction and (ii) interfere with loading of mPEG strands on the AuNP surface.

Preparation of FAM-PEG_y-SH

10 mg of H_2N-PEG_y-SH (y = 200, 500, 1000, or 5000 Da; Biochempeg) was dissolved in 900 μL of conjugation buffer (0.1 M sodium bicarbonate solution, pH 8.3–8.5). Then, two molar excess of N-hydroxysuccinimide ester of fluorescein (FAM-NHS; Lumiprobe) was dissolved in 100 μL of DMSO (J&K Scientific). The two solutions were combined, and the resulting 1 mL of reaction mixture was vortexed at RT for 4 h. The FAM-PEG_y-SH conjugate was purified using dialysis against Nanopure water in a 1000 Da dialysis bag (Spectrum Laboratories) for 3 d. Finally, the FAM-PEG_y-SH solution was freeze dried and stored at -20 °C.

Preparation of FAM-conjugated Au_2-PEG_{200} NPs

A mixture of FAM-PEG_{200}-SH and unlabeled mPEG_{200}-SH (Biochempeg) at a mole ratio of 1:49 was used for the synthesis. $HAuCl_4$ (0.02 mmol), mPEG_{200}-SH (0.0588 mmol), and FAM-PEG_{200}-SH (0.0012 mmol) were dissolved in 7 mL of a 6:1 mixture of methanol/acetic acid (v/v). Next, 2 mL of 0.2 M $NaBH_4$ (0.4 mmol in Nanopure water) was added dropwise to the reaction mixture with rapid stirring. After 2 h, a dark brown solution was formed, and the solvent was removed under vacuum at 40 °C using a rotary evaporator (R215, Buchi). After dissolving the residues in 5 mL of Nanopure water, the mixture was transferred to a 5–8 kDa molecular weight cut off membrane and dialyzed against Nanopure water for a minimum of three times over 72 h at RT. The concentration of NPs was then determined by ICP-MS.

Preparation of FAM-conjugated Au_3-PEG_{500}, Au_5-PEG_{1000}, Au_5-PEG_{5000}, and Au_{20}-PEG_{5000} NPs

A mixture of FAM-PEG_y-SH and unlabeled mPEG_y-SH at a mole ratio of 1: 49 was reacted with unmodified Au_x NPs (x = 3, 5, and 20 nm) at a total concentration of 10 PEG molecules per nm^2 of NP surface under stirring for 2 h. To remove the free PEG strands, the FAM-conjugated Au_3-PEG_{500}, Au_5-PEG_{1000}, Au_5-PEG_{5000} NPs were washed five times by centrifugation at 6000 rpm for 6 min, using centrifugal filters with a membrane size cutoff of 50 kDa (Merck Millipore). The Au_{20}-PEG_{5000} NPs were washed by centrifugation at 15,000 rpm for 30 min for five times. The concentration of NPs was then determined by ICP-MS.

Displacement of PEG strands from the AuNP surface

0.1 mL of DTT (Sigma) solution (1.0 M in 0.18 M phosphate buffer, pH = 8) was added to 0.1 mL of FAM-conjugated Au_x-PEG_y NPs (~5 μg of Au for Au_{20} NPs, ~10 μg of Au for Au_5 NPs and ~40 μg of Au for Au_2 and Au_3 NPs) to displace the conjugated PEG strands from the AuNP surface. The mixture was swirled overnight to allow for complete displacement of the PEG strands and aggregation of the AuNPs. The mixture was centrifuged at 15,000 rpm for 30 min.

Note: The relative DTT and NP concentrations added is important because we anticipated that the loading of PEG strands differs for each gold core size. A 20-nm gold core can accommodate more PEG strands than a 3-nm gold core. When reacting Au_x-PEG_y NPs with DTT for releasing the PEG strands and later calculating the amount of released strands to calculate the original PEG loading on the gold core, we tuned the amounts of Au_x-PEG_y NPs added to a constant DTT concentration of 1 M to ensure that all PEG strands were displaced from the gold cores upon overnight incubation, as evidenced by the gravitational sedimentation of NP aggregates. If displacement becomes complete, the solution will change color from pale red to colorless.

Calculation of PEG loading on the AuNP surface

150 µL of the supernatant solution was pipetted into a 96-well microplate to measure the FAM fluorescence in a Multiskan GO UV-absorbance microplate reader (Thermo Fisher Scientific). The excitation wavelength and emission wavelength of FAM were 495 nm and 520 nm, respectively. The final concentration of AuNPs was determined by inductive coupled-plasma mass spectrometry (ICP-MS; Agilent 7900) with reference to a standard curve of known gold concentration in parts per billion (ppb). The concentration of FAM-PEG-SH was calculated with reference to a standard curve. For all sizes of Au_x-PEG_y NPs, the concentration of mPEG strands was obtained by multiplying the concentration of FAM-PEG-SH strands in the microplate by 50 because the original ratio of FAM-PEG_y-SH to $mPEG_y$-SH ratio added to the unmodified AuNPs was 1: 49. The mPEG-SH loading per AuNP was calculated by dividing the concentration of mPEG-SH by the concentration of AuNPs.

For Au_3-PEG_{500}-FA_z NP:

Preparation of FAM-conjugated Au_3-PEG_{500}-FA_z NPs

FAM-conjugated Au_3-PEG_{500}-FA_{32} NP: We followed the same synthesis procedures as described for preparing Au_3-PEG_{500} NPs above, except for substituting $mPEG_{500}$-SH with FA-PEG_{500}-SH (BiochemPEG). That is, a mixture of FAM-PEG_{500}-SH and unlabeled FA-PEG_{500}-SH at a mole ratio of 1: 49 was reacted with unmodified Au_3 NPs, keeping a total concentration of 10 PEG molecules per nm^2 of NP surface.

FAM-conjugated Au_3-PEG_{500}-FA_{16} NP: A mixture of FAM-PEG_{500}-SH, unlabeled FA-PEG_{500}-SH, and unlabeled $mPEG_{500}$-SH at a mole ratio of 1: 49: 50 was reacted with unmodified Au_3 NPs, keeping a total concentration of 10 PEG molecules per nm^2 of NP surface.

FAM-conjugated Au_3-PEG_{500}-FA_8 NP: A mixture of FAM-PEG_{500}-SH, unlabeled FA-PEG_{500}-SH, and unlabeled $mPEG_{500}$-SH at a mole ratio of 1: 9: 30 was reacted with unmodified Au_3 NPs, keeping a total concentration of 10 PEG molecules per nm^2 of NP surface. We chose a slightly higher FAM-PEG_{500}-SH: FA-PEG_{500}-SH of 1:9 (not 1:49) to ensure sufficient fluorescence signals for detecting the reduced loading of FA-PEG_{500}-SH on the NP.

For FAM-conjugated Au_3-PEG_{500}-FA_z NPs of all FA loadings, the resultant NPs were washed by centrifugation at 6000 rpm for 6 min, using centrifugal filters with a membrane size cutoff of 50 kDa (Merck Millipore) for five times to completely remove the unconjugated PEG strands.

Displacement of PEG strands from the AuNP surface

We followed the same procedures as described for displacing PEG strands from Au_3-PEG_{500} NPs above. That is, 0.1 mL of DTT solution (1.0 M in 0.18 M phosphate buffer, pH = 8) was added to 0.1 mL of FAM-conjugated Au_3-PEG_{500}-FA_z NPs (~40 µg of Au) to displace the conjugated PEG strands from the AuNP surface overnight.

Calculation of FA-PEG loading on the AuNP surface

We followed the same procedures as above for centrifugation and measuring the fluorescence of the supernatant. The final concentration of AuNPs was determined by ICP-MS with reference to a standard curve of known gold concentration in ppb. The concentration of FAM-PEG-SH strands was calculated with reference to a standard curve. For Au_3-PEG_{500}-FA_{16} and Au_3-PEG_{500}-FA_{32} NPs, the concentration of FA-PEG strands on the NP surface was obtained by multiplying the concentration of FAM-PEG-SH strands in the microplate by 50 because the original ratios of FAM-PEG-SH to FA-PEG-SH ratio added to the unmodified Au_3 NPs of were 1: 49. For Au_3-PEG_{500}-FA_8 NPs, the concentration of FA-PEG strands on the NP surface was obtained by multiplying the concentration of FAM-PEG-SH strands in the microplate by 10 because the original ratio of FAM-PEG-SH to FA-PEG-SH ratio added to the unmodified Au_3 NPs of was 1: 9. The FA-PEG-SH loading per AuNP was calculated by dividing the concentration of FA-PEG-SH by the concentration of AuNPs.

Method II: Indirect measurement of excess thiolated PEG strands unattached to the gold core during synthesis of Au_x-PEG_y NP

Unmodified Au_x NPs were functionalized with $mPEG_y$-SH at a concentration of 10 PEG molecules per nm^2 of NP surface under stirring for 2 h. For Au_2-PEG_{200} NP only, an extra step of rotary evaporation (Buchi) was used to remove the organic solvents (methanol and acetic acid) from the reaction. The as-

synthesized Au_x-PEG_y NPs were collected without washing to retain the unbound $mPEG_y$-SH strands. 1 mL of the aqueous as-synthesized Au_x-PEG_y NP solution (containing ~50 µg of gold) was pelleted by ultracentrifugation at 220,000 ×g at 4° C for 30 min (Beckman Coulter, TLA-120 Rotor), and the free PEG strands in the supernatant were collected for analysis. A thiol fluorescent detection kit (Invitrogen; catalog no.: EIARSHF) was used to measure the concentration of excess unbound PEG-SH strands. Subtraction of the amount of unbound PEG-SH strands from the known total amount of PEG-SH strands initially added to the unmodified gold cores will infer the loading of PEG on the gold core. The $mPEG_y$-SH loading per NP was calculated by dividing the concentration of $mPEG_y$-SH attached to the gold core by the concentration of gold NPs (as determined by ICP-MS).

Note: Method II is not suitable for measuring the PEG loading on Au_3-PEG_{500}-FA_{32} NP. Existing thiol fluorescent quantification kits in the market as well as the Ellman's reagent often used for thiol quantification (based on absorbance) all have an absorption peak wavelength near the second absorption peak wavelength of FA (370–400 nm), leading to undesirable interference of the fluorescence or absorbance readout. Below, we will employ thermogravimetric analysis (TGA) as an additional method to verify the PEG loading.

Method III: Thermogravimetric analysis (TGA)

A concentrated solution of Au_x-PEG_y NP or Au_3-PEG_{500}-FA_{32} NP in Nanopure water was dried in an open alumina crucible at 80 °C for at least 2 h. This drying process would continue until the dried NP material on the crucible was at least 1 mg in weight. The sample was analyzed by PerkinElmer TGA6, with a heating rate of 10 °C/min from RT to 600 °C and nitrogen gas purging at a flow rate of 20 mL/min. The organic component of the NP (PEG) was found from the percent mass loss over the temperature range of 100–500°C, leaving behind gold (inorganic component) in the crucible after heating. Loading of PEG or PEG-FA strands on the gold core (σ_{TGA}) was calculated using the following equation (7):

$$\sigma_{TGA} = \frac{\frac{wt\%_{shell}}{wt\%_{core}} \rho_{core} \frac{4}{3} \pi r_{core}^3 N_A}{MW 4\pi r_{core}^2}$$

σ_{TGA}: Loading of PEG strands on gold NP. Unit: Number of strands per surface area.

$wt\%_{shell}$: Relative mass of PEG determined from TGA data.

$wt\%_{core}$: Relative mass of gold determined from TGA data.

ρ_{core}: Density of gold = 19.6 g/cm^3. r_{core}: Radius of gold core.

N_A: Avogadro constant. MW: Molecular weight of PEG strand.

Characterization of blood serum proteins onto NPs

Collection and purification of serum proteins bound

Typically, 100 µL of 500 nM Au_3-PEG_{500}-FA_{32} NPs or Au_3-PEG_{500} NPs were mixed with 100 µL of fresh blood serum collected from UUO mice (7 days post-UUO surgery) at 37 °C for 1 h to allow for protein adsorption. Next, following a previously published protocol (8), the protein-coated NPs were floated on top of a 60% and a 40% sucrose layer and centrifuged at 180,000 ×g for 1 h to separate the NP-protein complexes from free proteins. Following centrifugation, the top protein layer was removed, and the NP-protein complex was recovered from the bottom of the centrifuge tube. Sucrose was then removed from the NP-protein complexes by filtration through a 3 kDa amicon spin filter (Merck Millipore). After five washes with 0.5 × PBS, the sucrose gradient purification steps were repeated for a second wash. Next, 25 µL of resuspended NPs pellet was added to 10 µL of Laemmli sample buffer (4×; Bio-Rad) and 5 µL of 4.8 M DTT, followed by incubation at 70°C for 1 h with shaking to release the adsorbed proteins. The NPs were removed by centrifugation at 13,500 rpm at 4°C, and the 40 µL of supernatant containing the desorbed proteins was transferred to a new tube. Next, 950 µL of 10% (w/v) trichloroacetic acid (TCA, Sigma) in acetone was added and incubated at −80 °C overnight. Precipitates were centrifuged at 18,000 ×g at 4 °C for 15 min, and the supernatant was discarded. The pellet was dissolved in 500 µL of 0.03% (w/v) sodium deoxycholate (Sigma) in ultrapure distilled water (Invitrogen), precipitated by

adding 100 µL of 72% (w/v) TCA in acetone, and incubated on ice for 30 min. The precipitate was pelleted by centrifugation at 18,000 ×g at 4 °C for 15 min, and the supernatant was discarded. The pellet was washed in 1 mL of acetone at −20 °C for 30 min, left to dry, and dissolved in 50 µL of 50 mM ammonium bicarbonate (Thermo Fisher) in ultrapure distilled water.

Characterization of desorbed proteins (8, 9)

PAGE: Mercaptoethanol (0.5 µL) (Sigma) was added to 40 µL of the desorbed proteins in Laemmli sample buffer. The mixture was heated at 95 °C for 5 min with shaking. 6 µL of denatured proteins and 6 µL of Precision Plus Protein Dual Color Standards (Bio-Rad) were loaded onto a 4–20% Mini-PROTEAN TGX Precast Protein Gradient Gel (Bio-Rad) in tris-glycine SDS running buffer (Bio-Rad). After resolving the bands at 120 V for 90 min, the gel was fixed with 40% (v/v) ethanol and 10% (v/v) acetic acid in water for 2 h and stained with Flamingo Fluorescent Gel Stain (BioRad) for 3 h. The stained bands were visualized by the ChemiDoc Touch gel imaging system (Bio-Rad).

Protein concentration: 25 µL of purified desorbed proteins or different concentrations of serially diluted bovine serum albumin (BSA, Pierce) were transferred into a 96-well plate. After adding 200 µL of freshly prepared BCA Protein Assay working reagent (Pierce) to each well, the samples were incubated at 60°C for 30 min. Absorbance at 562 nm was measured by a microplate reader. Protein concentrations were calculated using BSA as standard of calibration.

Proteomics analysis of purified desorbed proteins

Preparation: 15 µL of 2–8 mg/mL purified desorbed proteins were mixed with 1 µL of 100 mM DTT and incubated at 56 °C for 1 h. 1 µL of 500 mM iodoacetamide (Sigma) was added and the solution was incubated for 60 min in the dark at RT to alkylate cysteines. 1 µg of proteomics-grade trypsin (Promega) was added to each sample, followed by overnight digestion at 37 °C. After adding 100 µL of extraction solution [5% trifluoroacetic acid (TFA)-50% acetonitrile (ACN)-45% ddH_2O] to each sample, the mixture was incubated in a 37°C-water bath for 1 h and sonicated for 5 min. The extract was transferred to a fresh microcentrifuge tube and the extraction step was repeated.

LC-MS/MS: All reagents used were chromatography grade (Thermo Fisher Scientific). The extracted peptides were lyophilized and resuspended in 10 µL of 0.1% formic acid for separation using an Orbitrap Eclipse Mass Spectrometer (Thermo Fisher Scientific), equipped with a 150 µm×15 cm in-house made column packed with Acclaim PepMap RPLC C18 (1.9 µm, 100 Å, Dr. Maisch GmbH). The organic gradient was driven by the Nanoflow UPLC system over 120 min using Buffer A (0.1% formic acid in water) and Buffer B (20% 0.1% formic acid in water and 80% acetonitrile) at a flow rate of 600 nL/min. The gradient was held from 4% to 8 % B for 3 min, from 8 % to 28% B for 86 min, from 28 % to 40% B for 20 min, from 40% to 95% B for 1 min, and from 95% to 95% B for 10 min. Eluted peptides were directly sprayed into the mass spectrometer. Ten MS/MS data-dependent scans were acquired simultaneously with one high-resolution (60000 at 400 m/z) full-scan mass spectrum to provide the amino acid sequence and mass-to-charge ratio for the selected peptide ions.

Data analysis: Raw MS files were analyzed and searched against the Uniprot-Mus musculus (Mouse) protein database based on the species of samples using MaxQuant (1.6.2.10) with the following parameters. Protein modifications were carbamidomethylation (C) (fixed), oxidation (M) (variable). Enzyme specificity was set as trypsin. Maximum missed cleavages were set as 2. Precursor ion mass tolerance was set as 20 ppm. MS/MS tolerance was set as 20 ppm. Only high confident identified peptides were chosen for protein identification analysis.

Search Tool for the Retrieval of Interacting Genes/Proteins (STRING) analysis

The 20 most abundant proteins adsorbed to the NP and the two kidney tubule receptors of interest, namely folate receptor (FOLR1) and megalin, were input into the STRING web server (accessed 31 January 2023)(10). We applied a STRING threshold of 0.400 to filter our list of predicted interactions, which corresponds to only a 40% chance of a predicted interaction being true. The complete list of predicted interactions with scores > 0.400 was exported.

Kinome profiling

Kinase panel screening and IC_{50}

Effect of Au_3-PEG_{500}-FA_{32} NPs on the activity of various kinases was assessed by SelectScreen Kinase Profiling Service (Thermo Fisher). After verifying the NP stock solution (20 µM; 100×) by ICP-MS, the

NP samples, prepared by dialyzing NPs in water against absolute DMSO, were sent for testing using the Z'LYTE biochemical assay. Briefly, Z'LYTE uses a fluorescence-based, coupled-enzyme format based on the differential sensitivity of phosphorylated and non-phosphorylated peptides to proteolytic cleavage. The NP sample was screened in 1% DMSO (final) in the well at a single concentration of 200 nM (1×). To calculate the IC_{50} value of a screened high-performing kinase (with at least 80% inhibitory activity), the NP sample was subject to 10-point, 3-fold serial dilutions from the starting NP concentration of 200 nM (1×) for constructing the dose-response curve. Assays were conducted using the ATP concentration as indicated in the corresponding tables, depending on the format of detection. For "Direct Format" that operates through phosphorylation and activation of a synthetic peptide substrate for a given kinase, [ATP] was set at its apparent K_m value ($K_{m,apparent}$), as previously determined by the Z'-LYTE assay. For "Cascade Format" that operates through phosphorylation and activation of the inactive downstream kinase of a given kinase, [ATP] was set at 100 µM.

Data analysis

Percent phosphorylation (% Pho) was determined with reference to the 0% phosphorylation (or 100% inhibition) control (which contains no ATP and therefore exhibits no kinase activity) and the 100% phosphorylation control (which contains the phosphorylated peptide as the same sequence as the peptide substrate). Control wells do not include any kinase inhibitors. Percent inhibition values were calculated by this equation: % Inhibition = $[1 - \% Pho_{NP} / \%Pho_{0\%\ inhibition\ ctrl}] \times 100$, whereby the 0% inhibition control contains the active kinase. IC_{50} values were fitted from the dose-response curves based on model number 205 of XLfit from IDBS.

In vitro **uptake of NPs by primary renal tubule cells**

Primary cell culture

Primary tubule cells were collected from Balb/c mice as reported (11, 12). Each kidney was digested in 2 mL of digestion buffer containing 2 mg/mL collagenase I (in Hank's Balanced Salt Solution (HBSS, Thermo Fisher) with gentle stirring at 37 °C for 30 min. The digested mixture was filtered through the 100-µm mesh (Corning) to collect the tubule fragments. Cells were cultured in complete culture medium [Roswell Park Memorial Institute (RPMI) 1640 (Thermo Fisher) supplemented with 10% FBS, 20 ng/mL epidermal growth factor (EGF; Sino Biological), 20 ng/mL basic fibroblast growth factor (bFGF; Sino Biological)] at 5% CO_2 and 37 °C.

Cytotoxicity of NPs

Cells were seeded in a 96-well plate at a density of 10^5 cells per well 24 h before the experiment. In each well, cells were incubated with 1.27 µg of AuNPs (or equivalently 100 nM Au_3-PEG_{500}-FA_{32} NPs) in complete culture medium for 24 h at 37 °C, the same gold mass to be used in cellular uptake experiments. After removing the NP-containing medium and rinsing the cells, the alamarBlue reagent (Invitrogen) was used to test the cell viability per the manufacturer's instructions by measuring the absorbance at 570 nm and 600 nm by a microplate reader. Reported data represent mean ± standard deviation (SD) from three independent experiments.

Quantification of cellular uptake by ICP-MS

Pre-seeded in 24-well plates until the cell population reached ~70% confluence, cells were incubated with 0.5 mL of 100 nM NPs (in complete culture medium) for 2 h. After that, the cells were rinsed with phosphate-buffered saline (PBS) twice and trypsinized (0.25% Trypsin-EDTA, Gibco) for cell counting by a hemocytometer. After centrifugation at 8,000 rpm for 5 min, the cell pellet was digested by 0.25 mL aqua regia (3:1 v/v ratio of 38% HCl and 68% HNO_3) overnight and diluted to 5 mL by the matrix solution (2% HCl, 2% HNO_3) for ICP-MS measurements (Agilent 7900) of the Au content associated with the cells. Data are shown as the mean ± SD from four biological replicates (i.e., 4 wells per treatment group).

Imaging of cellular uptake by confocal immunofluorescence

Seeded on glass slides in 24-well plates and grown to 60% confluence, cells were incubated with 500 µL of 100 nM Cy5.5-labelled NPs for 2 h. After rinsing with PBS for 3 times and fixation with 4% paraformaldehyde (Sigma), the cells were incubated with 3% BSA/0.1% Triton X-100 in PBS for 1 h and then stained with primary antibody in PBS at 4°C overnight. The primary antibodies include mouse monoclonal antibody against folate receptor (FOLR1; conjugated with Janelia Fluor 549; 5 µg/mL, FAB56461, Novus) and mouse monoclonal antibody against megalin (conjugated with Alexa Fluor 594;

2 μg/mL, sc-515772, Santa Cruz Biotechnology). The slides were stained by 1 μg/mL 4',6-diamidino-2-phenylindole (DAPI) for 10 min, washed and mounted with Antifade Mountant (P36980; Thermo Scientific) for visualization under a confocal laser scanning microscope (TCS SP8, Leica). The excitation wavelengths of DAPI, Janelia Fluor 549, Alexa Fluor 594 and Cy5.5 are 405 nm, 549 nm, 594 nm, and 651 nm, respectively. The emission wavelength ranges of DAPI, Janelia Fluor 549, Alexa Fluor 594, and Cy5.5 are 415–500 nm, 560–650 nm, 600–700 nm, and 670–790 nm, respectively.

Ex vivo binding of NPs to renal tubules

Competitive binding of NPs to the UUO kidney

Freshly harvested from UUO mice on Day 7 post-surgery, the UUO kidney was cut into halves along the frontal plane. Each half was cut into 3 tissue blocks, resulting in 6 kidney blocks per kidney. The tissue blocks were placed in a 48-well plate and pretreated with 0.3 mL of serum-containing RPMI 1640 medium with different contents, including (i) blank medium, (ii) medium with 250 nM antibody against folate receptor (ab67422, Abcam), (iii) medium with 250 nM antibody against megalin (ab76969, Abcam), and (iv) medium with 250 nM antibody against folate receptor and 250 nM antibody against megalin. After gentle shaking (200 rpm) at 37 °C for 30 min, 0.3 mL of 100 nM NPs were added to each well and incubated for another 2 h. The tissue blocks were washed by PBS 3 times and digested with 0.25 mL of aqua regia for determining the amount of gold NPs associated by ICP-MS.

Tissue-level distribution of NPs in the UUO kidney

Fresh UUO kidney blocks were incubated with 50 nM NPs with gentle shaking at 200 rpm for 2 h. They were washed by PBS 3 times and digested in aqua regia for ICP-MS measurement.

Tissue paraffin blocks

Tissues were fixed in 10% buffered formalin (3.7% w/v) for 48 h then stored in PBS (0.1M, pH 7.5) at 4°C until tissue dehydration. Fixed tissues were dehydrated in ethanol, cleared in xylene, and embedded in paraffin blocks. Paraffin-embedded tissue sections (4 μm) were cut and mounted on Superfrost Plus™ Adhesion microscope slides (Thermo Scientific).

Immunohistochemistry (IHC)

Mouse kidney tissue

IHC staining was performed on paraffin sections with antigen retrieval (13). Tissue sections were deparaffinized and rehydrated. After placing slides in citrate buffer (10 mM citric acid, pH = 6.0), the slides were heated in the microwave oven for 3 min under high power (~95–100 °C) and for 20 more min under low power. After cooling the slides in the heated solution for 30 min, they were rinsed in distilled water twice and in PBS for 5 min. The slides were blocked with 2.5% normal horse serum (Vector Laboratories) for 2 h and incubated with 60 µL of primary antibodies [1 μg/mL for type I collagen (1310-01; Southern BioTech), 0.048 μg/mL for α-SMA (ab150301; Abcam), 0.625 μg/mL for folate receptor (ab67422; Abcam), and 1.25 μg/mL for CD3 (ab16669; Abcam)] at 4 °C overnight. Slides were washed in PBS, treated with 3% H_2O_2 (Merck Millipore) for 30 min, rinsed, and incubated with 50 µL of secondary antibodies (ImmPRESS HRP Polymer Detection Kit, Vector Laboratories) for 30 min. The sections were developed sequentially using 3,3'-diaminobenzidine (DAB) enzyme substrate (ImmPACT™ DAB, Vector Laboratories) for 2 min. Slides were counterstained with Mayer's hematoxylin for 3 min, washed in distilled water, dried in 90% ethanol, and mounted with xylene-based mounting medium. Bright-field images were taken with a Nikon Eclipse Ni (DS-Ri2) microscope.

Human kidney tissue

Paraffin sections of human kidney biopsy specimens with a diagnosis of hypertensive nephrosclerosis were used. The patient samples originated from an archived database in which they were delinked from their clinical data and identifier, so we cannot confirm if our 2 chosen sections in each tissue group came from the same patient in Fig. 2A. We chose n=2 due to limited samples available in the database. IHC analysis of the expression of human folate receptor largely followed the procedures for analyzing the expression of mouse folate receptor, except for using 0.313 μg/mL of primary antibody for folate receptor (ab67422; Abcam).

In vivo distribution of NPs

Organ-level distribution of NPs

For NP of all sizes, equal amounts of Au (100 µg) were injected into each animal at the desirable timepoint and sacrificed for organ collection 24 h post-injection. Because Au_x-PEG_y NPs have different sizes, the equivalent number of NPs per injected dose was different for each NP size was 7.2×10^{14} NPs for 2-nm gold NP, 2.5×10^{14} NPs for 3-nm gold NP, 7.9×10^{13} NPs for 5-nm gold NP, and 1.2×10^{12} NPs for 20-nm gold NP. For the UUO model, the NPs were injected into mice 7 d, 14 d, or 28 d post-UUO surgery. Tissues were weighed and digested with 0.5 mL of aqua regia (3:1 v/v ratio of 38% HCl and 68% HNO_3) for 4 d at RT, unless otherwise stated. Whole livers were digested with 4 mL of aqua regia, and 0.3 mL of blood was digested with 1.2 mL of aqua regia for 4 d at RT. The digested samples were diluted to a 2% HCl, 2% HNO_3 solution with Nanopure water and then filtered with 0.1 µm acid resistant (cellulose nitrate and cellulose acetate) filter (HINOE) using a 10 mL syringe. Calibration standards of known gold concentration was prepared to convert counts of gold ion to known gold concentration. ICP-MS was used to measure the gold content in the sample.

Effect of disease stage on organ-level distribution

The different stages of tubulointerstitial fibrosis are characterized by the number of days after UUO surgery. We studied the UUO kidney on Days 7, 14, and 28 post-UUO surgery to indicate progressively severe fibrosis. Mice were injected with 100 µg of Au_x-PEG_y NPs via the tail vein and sacrificed 24 h after NP injection. Then, the internal organs (brain, lung, heart, liver, spleen, pancreas, small intestine, and kidneys) and blood were extracted for the detection of their bulk gold contents using ICP-MS. As the UUO kidney becomes progressively smaller with more severe fibrosis and apoptosis, the amount of gold in each organ was normalized to %ID/g to eliminate any bias due to the difference in tissue mass.

Intrarenal tissue-level distribution of NPs

For all types of NPs, equal amounts of Au (100 µg) were injected into each animal. The NP solutions were injected into UUO mice 7 d post-UUO surgery and sacrificed 24 h post-injection (or 8 d post-UUO surgery). Glomeruli isolation was performed as described previously with a slight modification (14). In brief, anesthetized mice were perfused with 8×10^7 Dynabeads (Thermo Fisher Scientific) diluted in 40 mL of PBS through the heart. Then, the kidneys were removed, minced, and digested in a digestion solution [1 mg/mL collagenase A (Roche), 100 U/mL deoxyribonuclease I (Roche) in Hanks' balanced salt solution (HBSS) (Thermo Fisher Scientific)] at 37 °C for 40 min with gentle agitation. The digested tissue was pressed through a 100 µm cell strainer (BD Falcon) using a flattened pestle, and the cell strainer was then thoroughly washed with 5 mL of ice-cold HBSS. The cell suspension was centrifuged at 200 × g for 5 min at 4 °C. The first supernatant was transferred to a new tube (labelled "interstitial space"), and the cell pellet was resuspended in 2 mL of HBSS. The Dynabeads-containing glomeruli were gathered by a magnetic particle concentrator (Dynal). The second supernatant (including the cells not attracted to the magnet) was carefully pipetted into a separate tube and stored on ice (labelled "tubules + tubulointerstitial cells"). All samples were freeze-dried and digested with aqua regia for ICP-MS measurements, as described in the previous section.

Cellular-level distribution of NPs by TEM imaging

Tissue blocks (~1 mm^3) were fixed with glutaraldehyde (2.5% in 0.1 M phosphate buffer, pH = 7.4) at RT for 2 h, and at 4°C for overnight. Then tissue blocks were washed stained with osmium tetroxide (1%) at 4 °C for 2 h. The tissue blocks were gradually dehydrated with increasing ethanol gradients and propylene oxide, embedded in Epon 812 resins (Electron Microscopy Sciences), and polymerized at 55 °C for 48 h. Ultrathin sections of ~70 nm thick were deposited on 200-mesh copper grids (EMS) and stained with 4% (w/v) uranyl acetate (EMS) in 50% methanol/water) and Reynolds lead citrate (Sigma) for visualization under TEM at 100 kV (Hitachi H7700). Elemental analysis was performed using a Field Emission Electron Microscope STEM (JEM-2100F, JEOL Ltd.) equipped with an EDX analysis system.

Silver enhancement staining

Paraffin-embedded tissue sections of 4 µm thick were deparaffinized in xylene (5 min × 3 times) and rehydrated through a series of ethanol (100%, 90%, 70%; 3 min × 2 times at each ethanol concentration), and deionized water (Milli Q) (5 min × 5 times). The rehydrated tissue sections were stained by the Silver Enhancement Kit for Light and Electron Microscopy (Ted Pella). The silver enhancement solutions, Solution A (silver salt) and Solution B (initiator), were mixed at a 1:1 ratio right before use. A drop of the mixture (~50 µL) was applied to the tissue section for 20 min under normal laboratory lighting. Next, the tissue sections were rinsed with Milli Q water (3 min × 3 times), followed by counterstaining with Mayer's Hematoxylin (blue-purple nuclear stain; Vector Laboratories) or methyl green (blue-green nuclear stain; Vector Laboratories) for 10 min. Bright-field images were acquired using the Nikon Eclipse Ni (DS-Ri2) microscope.

Confocal reflectance microscopy

Confocal reflectance images of tissue sections overlaid with true colour images were obtained by using a Leica SP8 confocal microscope. Confocal images were produced in the reflectance mode with 20× objectives under 488 nm excitation (15). True colour images were produced by overlaying the red, green, and blue (RGB) channels in the transmitted light imaging mode.

Hepatobiliary clearance or renal clearance of NPs

Mice were injected i.v. with Au_x-PEG_y or Au_3-PEG_{500}-FA_{32} NPs at a constant gold (Au) mass of 100 µg per 100 µL of injection. Then, the mice were housed in metabolic cages (Lab Products) for collecting their feces and urine at various time points post-injection. Urine and feces samples were digested with 1.2 mL and 2 mL of aqua regia respectively, for 4 d at RT for ICP-MS measurements.

Efficacy evaluation of NPs

Endotoxin level of NPs

Per our previous report (9), we used the Pierce LAL Chromogenic Endotoxin Quantification Kit (Thermo Fisher Scientific) per the manufacturer's instructions. 50 µL of each standard or supernatant of NPs were transferred to a 96-well plate that was prewarmed to 37 °C. 50 µL of the LAL reagent was added to each well, and the plate was incubated at 37 °C for 10 min. 100 µL of chromogenic substrate solution (prewarmed to 37 °C) was added to each well, and the plate was incubated at 37 °C for 6 more minutes. 100 µL of 25% acetic acid was added to each well. Absorbance at 405 nm was measured by a microplate reader. Endotoxin levels were calculated based on a calibrated standard curve.

Protein extraction from kidney tissue

For type I collagen, tissue lysates were prepared from the mouse kidney per a previous protocol (16) with slight modification. 50 µg of kidney cortices were homogenized (Biospec Products) in 0.6 mL of 0.5 M acetic acid containing Pierce Protease Inhibitors (Thermo Scientific). The tissue lysates were agitated for 48 h at 4°C and centrifuged at 16,000 × g for 20 min at 4°C.

Efficacy evaluation by IHC

The expression levels of type I collagen, α-SMA, and CD3-positive cells in tissue sections were measured by IHC. Images were taken at the cortex area of the kidney. Of the 9 mice in each treatment group, 3 tissue sections from different layers (at least 200 µm away from each other) were obtained from each kidney and stained. Each section was imaged in at least 6 different areas; so, at least 18 images per kidney were used for quantifying the percent positive area (for type I collagen and α-SMA) or number of CD3-positive cells. The averaged value of at least 18 images per kidney was displayed as one single data point on the stacked bar scatter plot.

Efficacy evaluation by western blot

20 µg of tissue lysate was electrophoresed through a 10% denaturing polyacrylamide gel (BioRad) and transferred to a polyvinylidene difluoride membrane (BioRad) at 100 V on ice for 2 h. After blocking in 5% BSA (Rockland) in Tris-buffered saline-Tween (TBST) buffer for 1 h, the blots were incubated with 2 µg/mL primary rabbit antibody against type 1 collagen (ab270993; Abcam), and 0.09 µg/mL primary rabbit antibody against GAPDH (CST-2118; Cell signaling) diluted in TBST containing 5% BSA

overnight at 4°C. Lastly, the blot was incubated with 1 µg/mL secondary goat antibody (conjugated with horse radish peroxidase) against rabbit (1706515; Bio-Rad) diluted in TBST containing 5% non-fat milk for 1 h. The membranes were treated with Clarity™ Western ECL Substrate (Bio-Rad) and the protein bands were visualized a ChemiDoc Touch Imaging System (Bio-Rad).

Toxicity test

Liver, heart, and spleen samples were fixed in 10% buffered formalin for 48 h and then stored in PBS at 4 °C. Paraffin-embedded tissue sections of 4 µm thick were stained with hematoxylin and eosin for examining tissue morphology by light microscopy. Blood, collected via an intracardiac puncture, was stored in a plain tube for biochemistry tests and in EDTA-coated tubes (Becton Dickinson) for hematology analysis. The blood samples were kept on ice and later sent to PathLab (Hong Kong) for analysis on the same day.

Mechanism for the efficacy of Au_3-PEG_{500}-FA_{32} NPs

Transcriptomic analysis

UUO mice were i.v. injected with Au_3-PEG_{500}-FA_{32} NPs (50 mg-Au/kg-mouse) or free FA (0.12 mg-FA/kg-mouse) on Day 7 post-UUO surgery, at the same dosage used for the efficacy studies. An untreated group of UUO mice was included as control. On Day 9 after UUO surgery (i.e., 48 h post-injection), the mice were sacrificed and the UUO kidneys were harvested. After cutting the UUO kidneys in half using a scalpel, one set of the halved kidneys was fixed in buffered formalin while the other set was snap frozen in liquid nitrogen and stored at -80 °C. The formalin-fixed samples were dehydrated as mentioned above and embedded in paraffin blocks to generate tissue sections for validating the RNA-seq data by IHC. The frozen samples were sent to Beijing Genomics Institute (BGI) for RNA extraction, RNA library construction, and bioinformatic analysis. A total of 9 samples were sequenced using the BGISEQ platform, with ~4.57 Gb generated per sample. The average mapping ratio with reference genome is 94.33%. Differential expressed genes (DEGs) detection, gene ontology analysis of DEGs, and other analysis based on gene expression were performed by BGI. Further analysis of the DEGs was performed by filtering the list of DEGs with a FPKM threshold of 2 for at least 6 out of the 9 UUO samples tested. GO terms, and DEGs with corrected p values (Q values) of 0.05 or less were considered significantly enriched. To ensure the gene expression changes are sufficiently large due to the treatment, we do not include those genes in which more than 3 samples contain FPKM values < 2 in our analysis. (If not, we will run into a situation in which most of the samples will contain near-zero FPKM values that will render our analysis less informative.) Moreover, we wish to capture the possibility in which Au_3-PEG_{500}-FA_{32} NP treatment can effectively inhibit a certain DEG to a very low level (with near-zero FPKM values) when compared to the untreated and free FA control groups. Therefore, we allow the selected DEGs to contain up to 3 near-zero FPKM values, or equivalently at least 6 samples with FPKM values > 2.

Detection of p38α

Frozen whole kidneys were grinded in liquid nitrogen with a mortar and pestle, and the crushed tissue was lysed in T-PER tissue protein extraction reagent (Thermo Fisher) containing Pierce Protease Inhibitors (Thermo Scientific) and phosphatase inhibitor cocktail (Bio-Platform) on ice for 30 min. Proteins in the supernatant were collected by centrifugation at 13,000 rpm for 10 min at 4 °C. The concentrations of protein across different cell samples were normalized using BCA protein assay, and the proteins were stored at -80 °C for western blot analysis.

Western blot

We follow the procedures above with minor changes. After blocking in 5% BSA (Rockland) in Tris-buffered saline-Tween (TBST) buffer for 1 h, the blot was incubated 0.046 µg/mL primary rabbit antibody against Phospho-p38 alpha (Thr180/Tyr182) antibody (MA5-15177, Invitrogen), 0.023 µg/mL primary rabbit antibody against p38 MAPK antibody (CST-9212, Cell Signaling), and 0.09 µg/mL primary rabbit antibody against GAPDH (CST-2118; Cell signaling) diluted in TBST containing 5% BSA overnight at 4°C. Lastly, the blot was incubated with 1 µg/mL secondary goat antibody (conjugated with horse radish peroxidase) against rabbit (1706515; Bio-Rad) diluted in TBST containing 5% non-fat milk for 1 h.

ELISA

Phosphorylation of p38α was measured using a p38 MAPK alpha (pT180/pY182) + total p38 MAPK alpha ELISA kit (AB126453-96T, Abcam). Per the manufacturer's instruction, 100 µL aliquots of proteins were used for measuring the ratio of phosphorylated p38α.

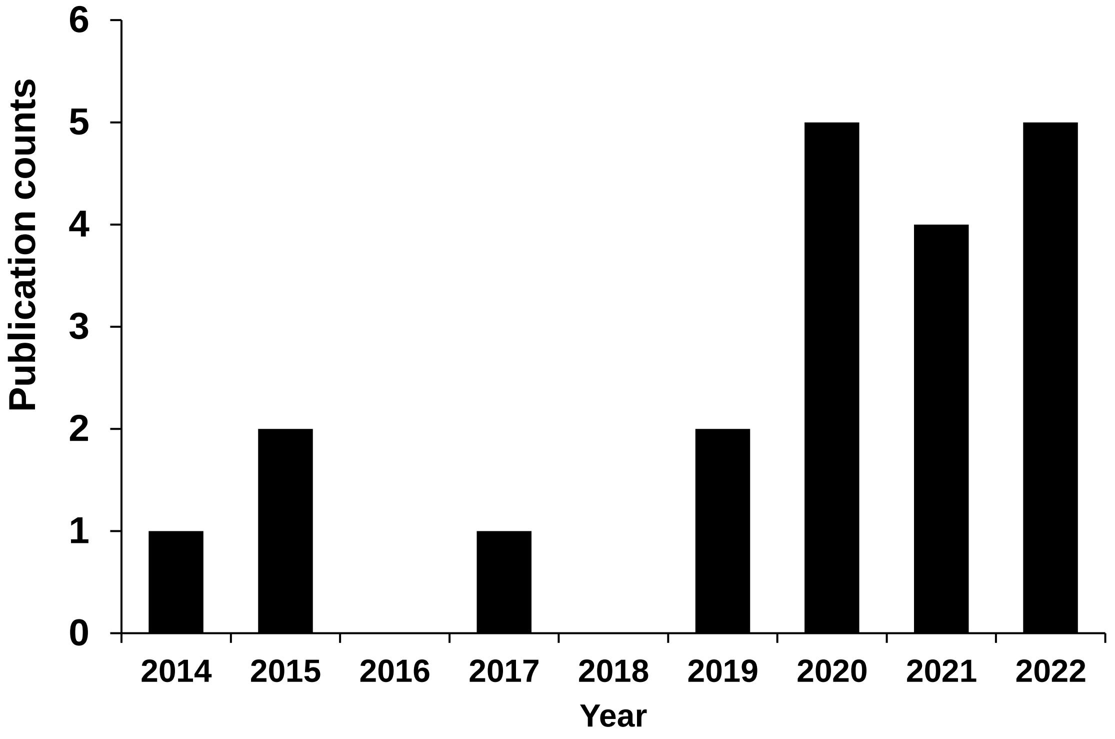

Fig. S1. Number of publications per year from 1956 to 2022 as revealed in the Web of Science and Google Scholar (accessed January 7, 2023) based on the defined search keywords of "renal fibrosis" and "nanoparticle". Note that Year 2014 marked the first year with non-zero publications related to both keywords. Duplicate publications from both engines were excluded.

Table S1. Summary of NP-based interventions for ameliorating renal fibrosis

(A) Tubulointerstitial fibrosis due to unilateral ureteral obstruction (UUO)

We consider an intervention preventive if the first administration takes place before Day 7 post-UUO surgery when tubulointerstitial fibrosis becomes established. Note: IV = intravenous; IP = intraperitoneal; PEG = poly(ethylene glycol); PLA = poly(lactic acid).

NP	Mode of intervention	Details of dosing	NP Size (nm)	Year
**Au_3-PEG_{500}-FA_{32} NP (This work)**	**Treatment**	**7 days post-UUO surgery. IV, 1 dose only, sacrifice on Day 14 post-UUO surgery.**	**<10 nm**	**N/A**
**JAK inhibitor (pyridine 6)-incorporated poly(lactic-co-glycolic acid) (PLGA) NP**	N.A.	Did not specify time of treatment, twice per week. IP, 2 mg/kg, sacrifice on Day 14 post-UUO surgery.	N.A.	2014 (17)

**Chitosan/siRNA-against cyclooxygenase type 2 NP**	Preventive	3 days prior to UUO surgery, every second day, IP, 0.5 mg/kg, sacrifice on day 3.	226	2015 (18)
**MicroRNA-146a-polyethylenimine NP**	Preventive	1 day prior to UUO surgery, every other day, IV, 5 nmol per mouse, sacrifice on Day 6 post-UUO surgery.	N.A.	2015 (19)
**Celastrol liposome**	Preventive	Day 5 post-UUO surgery, every day or every other day for a total of five times, IV, 1 mg/kg, sacrifice on Day 14 post-UUO surgery.	110	2020 (20)
**Co^{2+}-glutathione gold nano-assemblies (Au-D,L-lipoic acid-glutathione-Co)**	Preventive	7 days prior to UUO surgery, daily for 14 days, IP, 5 mg/kg, sacrifice on Day 14 post-UUO surgery.	6.7	2020 (21)
**Peroxisome proliferator-activated receptor gamma agonist (rosiglitazone)-loaded PLGA NP**	Preventive	Day 3 post-UUO surgery, daily for 7 days, IV, 8 µg per mouse, sacrifice on Day 10 post-UUO surgery.	600	2020 (22)
**Cerium oxide NP**	Preventive	Day 3 post-UUO surgery, every day, IP, low dose: 0.2 mg/kg, high dose: 2 mg/kg, sacrifice on day 14 post-UUO surgery.	~10	2020 (23)
**Farnesylthiosalicylic acid-loaded albumin NP**	Preventive	Day 2 post-UUO surgery; daily, IV, 20 mg/kg, sacrifice on Day 8 post-UUO surgery.	100	2021 (24)
**Gypenoside XLIZ PLGA NP**	Preventive	Day 1 post-UUO surgery, every other day, IV, low dose 2.5 mg/kg, high dose: 5 mg/kg, sacrifice on Day 7 post-UUO surgery.	128	2021 (25)
**Sorafenib-loaded PLGA NP**	Preventive	1 day post-UUO surgery, Days 1, 4, and 6, IV, 20 mg/kg, sacrifice on Day 8 post-UUO surgery.	~76	2022 (26)
**Ceria NP**	Preventive	1 day prior to UUO surgery, daily for 7 days, IV, 0.25 mg/kg, 0.5 mg/kg and 1 mg/kg, sacrifice on Day 7 post-UUO surgery.	12.7	2022 (27)
**Metformin-grafted chitosan NP**	Preventive	1 day post-UUO surgery, every day, IP, 117.5 mg/kg, sacrificed on Day 7 post-UUO surgery	150	2022 (28)

(B) Renal fibrosis due to nephropathy other than tubulointerstitial fibrosis

We consider an intervention “not determined” when the authors did not experimentally ascertain whether CKD was firmly established before the administration of NPs.

NP	Mode of intervention	Details of dosing	Animal model	NP size (nm)	Year
**Quercetin-loaded PEG-block-(poly(ethylenediamine l-glutamate)-graft-poly(ε-benzyloxycarbonyl-l-lysine) NP**	Not determined	When blood glucose content >16.7 mmol/L. Abdominal subcutaneous injection, 10 mg/kg, every day for 8 weeks.	Streptozotocin (STZ)-induced diabetic nephropathy	32	2017 (29)
**Pomegranate peel extract-stabilized gold NP**	Not determined	1 day after the onset of diabetes, blood glucose content >200 mg/dL; IP, 5, 15, 25 mg/kg every 2 days for 10 days.	STZ-induced diabetic nephropathy	20	2019 (30)
**Dexamethasone (DEX) – macrophage-derived microvesicle**	Treatment	Day 7 post adriamycin treatment, IV, 0.5 mg/kg, every 2 days for 2 weeks.	Adriamycin-induced nephropathy	140	2019 (31)
**Gold NP**	Not determined	After the onset of diabetes, blood glucose > 250 mg/dL; IP, 2.5 mg/kg every day for 7 weeks.	STZ-induced diabetic nephropathy	50	2020 (32)
**Enodin-incorporated monomethoxy-PEG-PLA-chitosan-2-mercaptobenzimidazole NP**	Treatment	8 weeks after surgery, low dose: 1.15 mg/kg, high lose: 4.6 mg/kg, colonic irrigation, every day for 8 weeks.	5/6 nephrectomy	100–200	2021 (33)
**Fe_3O_4 magnetic albumin NP**	Preventive	Same day as albumin administration, IV, 0.2 mg per injection, daily for 3 days.	Rab7-overexpressing transgenic, albumin induced mice	102	2021 (34)
**DEX/transforming growth factor beta 1 (TGF-β)-siRNA liposome-gold NP complex**	Not determined	3 days after 2 injections of venom. IV, 1.5 mg/kg DEX, 20 nmol/kg siRNA, every 2 days for 6 days.	Habu snake venom induced glomerulo-nephritis	123	2022 (35)
**DEX and captopril liposome-PLGA NP**	Not determined	After 2 injections of venom. IV, 1 mg/kg of DEX, and 2 mg/kg of captopril, every two days, for 1 week.	Habu snake venom induced glomerulo-nephritis	70–130	2022 (36)

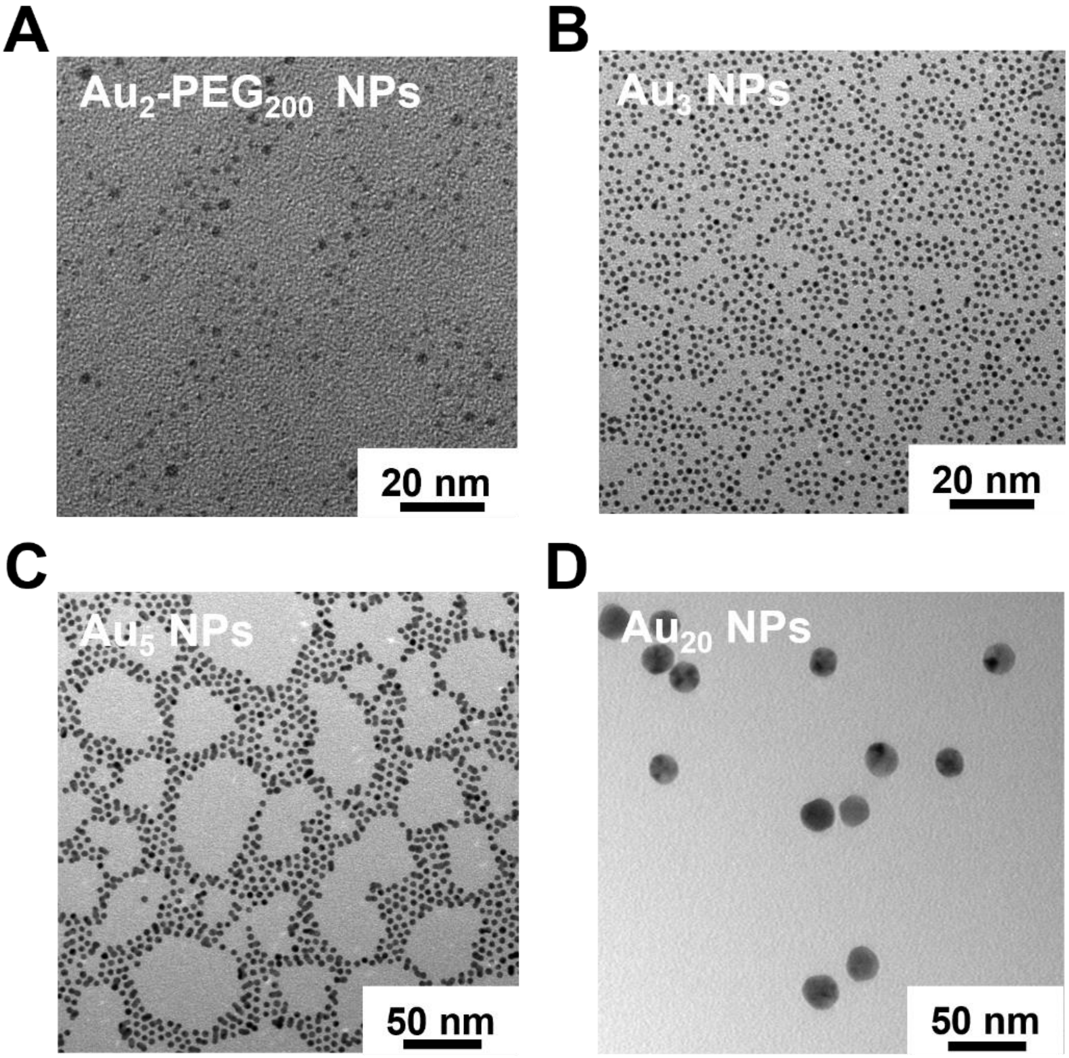

Fig. S2. Representative TEM images of Au_2-PEG_{200} NPs and unmodified Au_3, Au_5, and Au_{20} NPs. (A) Au_2-PEG_{200} NPs were synthesized by reducing $HAuCl_4$ in the presence of SH-PEG_{200}. Unmodified Au_x NPs of (B) ~3 nm and (C) ~5 nm in size were synthesized by reducing $HAuCl_4$ by sodium citrate and tannic acid. (D) Unmodified Au_{20} NPs were synthesized by reducing $HAuCl_4$ by sodium citrate. TEM images confirm the size and shape uniformity of Au_x NPs.

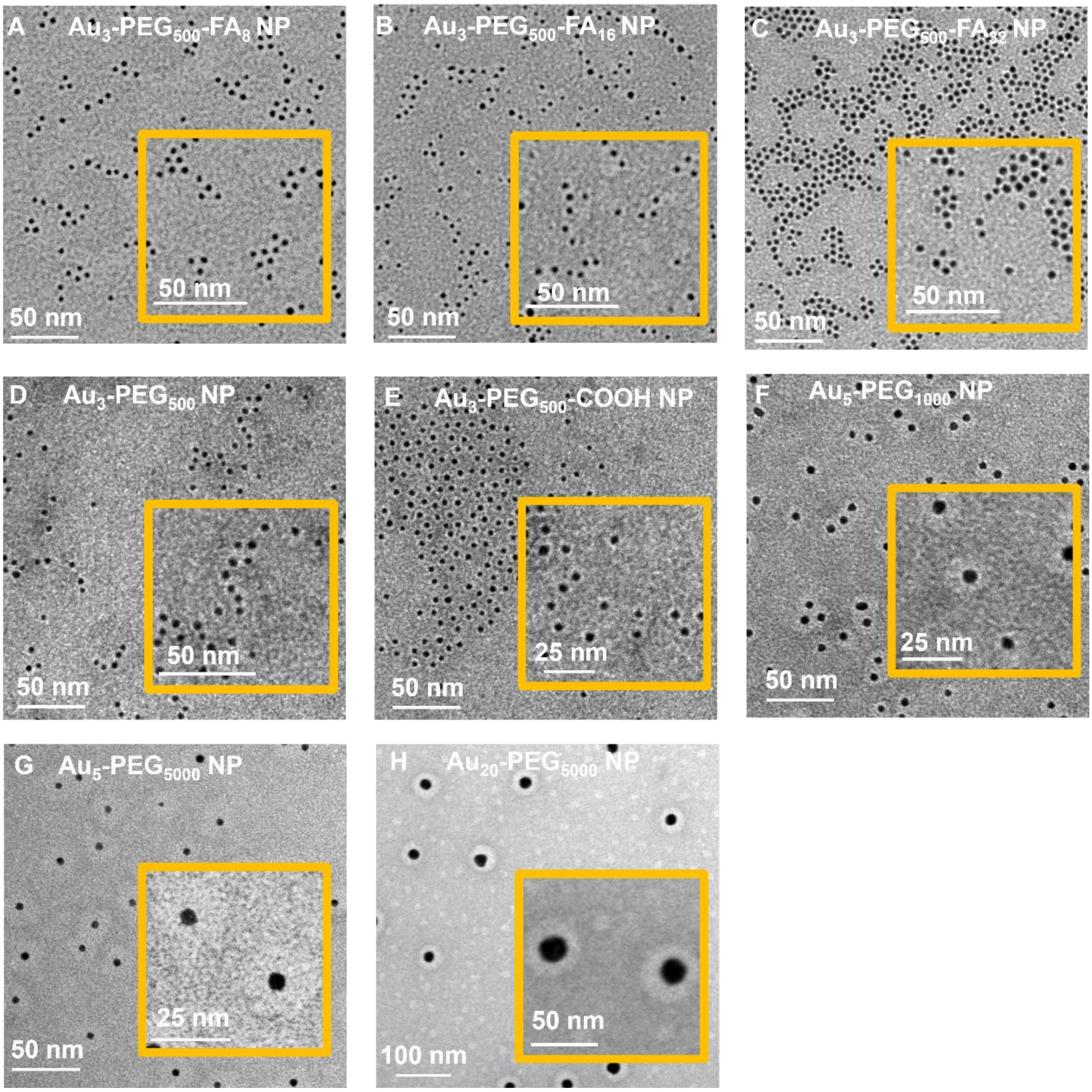

Fig. S3. Representative TEM images of (A) Au_3-PEG_{500}-FA_8, (B) Au_3-PEG_{500}-FA_{16}, (C) Au_3-PEG_{500}-FA_{32}, (D) Au_3-PEG_{500}, (E) Au_3-PEG_{500}-COOH, (F) Au_5-PEG_{1000}, (G) Au_5-PEG_{5000} and (H) Au_{20}-PEG_{5000} NPs with negative staining. Insets show the stained NPs at a higher magnification. Successful negative stain would produce contrast between the background and NPs. The PEG shell appears as a light halo around the gold core because of its lower electron scattering power relative to the gold core and the surrounding heavy-metal background stain. For Au_2-PEG_{200} NP, its PEG_{200} shell does not have enough contrast to be imaged.

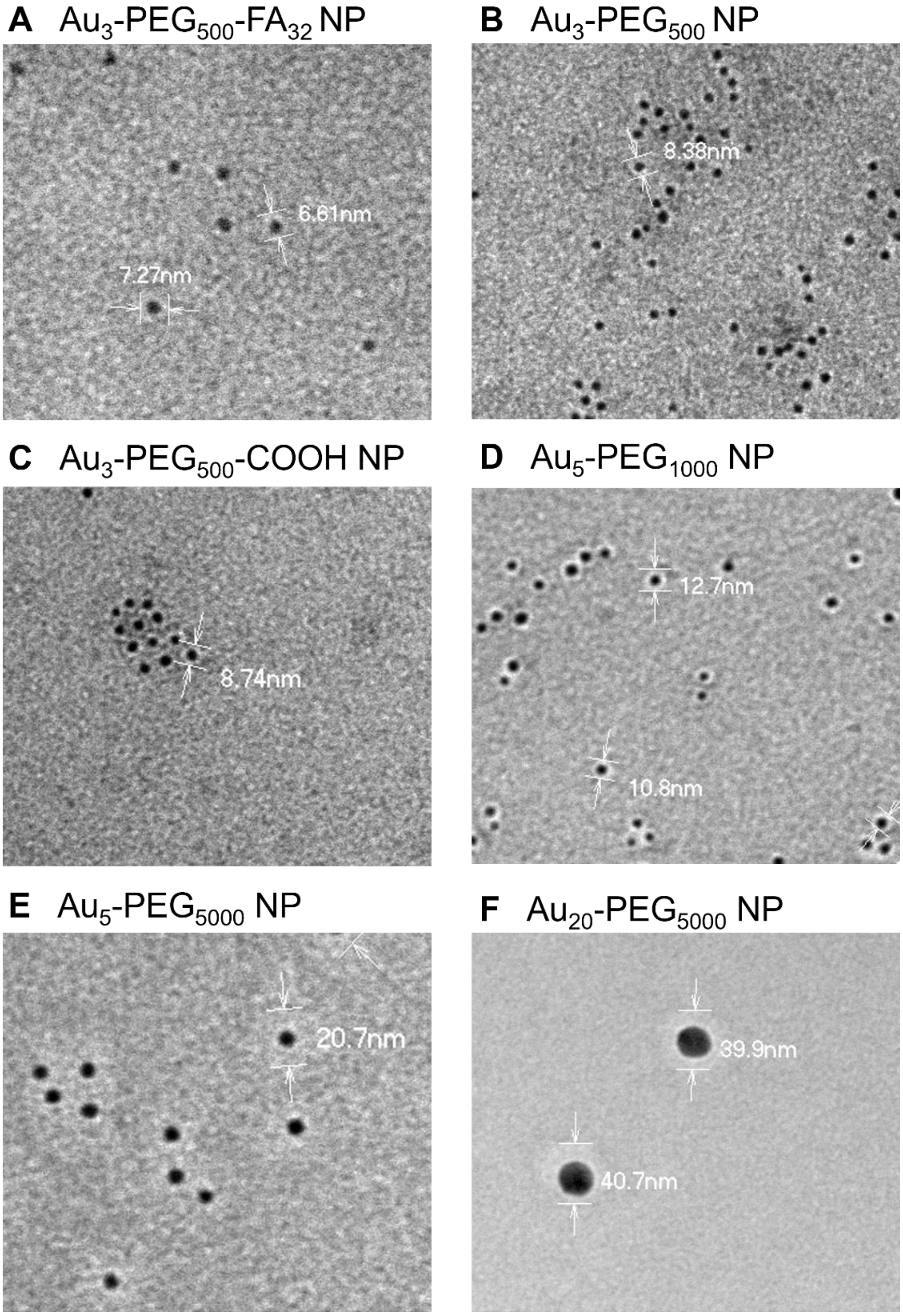

Fig. S4. Representative TEM images of (A) Au_3-PEG_{500}-$FA_{32,}$ (B) Au_3-PEG_{500}, (C) Au_3-PEG_{500}-COOH, (D) Au_5-PEG_{1000}, (F) Au_5-PEG_{5000} and (G) Au_{20}-PEG_{5000} NPs with negative staining, as measured using the length measuring function of the imaging software installed on-site at the Hitachi (H7700) TEM instrument. White arrows denote the boundary of the whole NP (Au core + PEG shell). The adjacent number indicates the respective physical diameters as directly obtained from the imaging software. The imaging contrast of (A) Au_3-PEG_{500}-$FA_{32,}$ (B) Au_3-PEG_{500}, (C) Au_3-PEG_{500}-COOH NPs was lower than those of the larger Au_x-PEG_y NPs probably because of their smaller Au core sizes and shorter PEG chain lengths. Still, the contrast of the NPs in (A)–(C) was sufficient to achieve defined measurements of physical sizes. For Au_2-PEG_{200} NP, its PEG_{200} shell does not have enough contrast to be imaged.

Characterization of Au_x-PEG_{500}-FA_z NPs

Table S2. Physicochemical properties of Au_x-PEG_y and Au_x-PEG_y-FA_z NPs. X = diameter of Au core (nm); y = molecular weight of PEG (Da); z = number of FA per NP. PDI = polydispersity index.

NP	Physical diameter of the Au core (nm) [a]	Physical diameter of Au core + PEG shell (nm) [a]	Hydrodynamic diameter in water at RT (nm) [b]	PDI	ζ-potential in 1 mM KCl at RT (mV)	No. of PEG strand/NP	No. of PEG strand/nm^2
Au_3 NP	3.4 ± 0.3	N.A.	3.9 ± 1.3	0.02 ±0.00	-55.7 ± 5.1	N.A.	N.A.
Au_5 NP	5.1 ± 0.5	N.A.	6.0 ± 0.2	0.03 ±0.01	-54.0 ± 4.3	N.A.	N.A.
Au_{20} NP	19.3 ± 2.7	N.A.	20.1 ± 0.5	0.12 ±0.01	-60.4± 2.4	N.A.	N.A.
Au_2-PEG_{200} NP	2.3 ± 0.6	N.A.	2.7 ± 0.2	0.01 ±0.02	-7.8 ± 1.7	164 ± 6.5	9.9 ± 0.5
Au_3-PEG_{500} NP	3.5 ± 0.3	8.4 ± 0.9	9.4 ± 0.4	0.01 ± 0.02	-8.6 ± 0.7	81 ± 7.8	2.3 ± 0.2
Au_3-PEG_{500} -COOH NP	3.4 ± 0.2	7.5 ± 0.8	9.5 ± 0.3	0.02 ± 0.03	-23.7 ± 1.0	131 ± 15.6	3.6 ± 0.4
Au_3-PEG_{500}-FA_8 NP	3.4 ± 0.3	8.6 ± 1.1	7.0 ± 0.4	0.04 ± 0.01	-8.3 ± 1.1	30 ± 3 (PEG)<br>7.7 ± 0.7 (PEG-FA)	0.6 ± 0.2 (PEG)<br>0.2 ± 0.0 (PEG-FA)
Au_3-PEG_{500}-FA_{16} NP	3.5 ± 0.2	6.7 ± 0.8	7.3 ± 0.5	0.03 ± 0.03	-10.7 ± 2.2	13 ± 1 (PEG)<br>17 ± 5 (PEG-FA)	0.6 ± 0.2 (PEG)<br>0.5 ± 0.1 (PEG-FA)
Au_3-PEG_{500}-FA_{32} NP	3.5 ± 0.5	7.4 ± 0.2	7.0 ± 0.2	0.06 ± 0.02	-13.5 ± 0.8	32 ± 6.3	0.9 ± 0.2
Au_5-PEG_{1000} NP	5.2 ± 0.6	13.7 ± 1.9	12.9 ± 0.4	0.03 ± 0.05	-10.3 ± 0.8	225 ± 12	2.9 ± 0.1
Au_5-PEG_{5000} NP	5.3 ± 0.8	20.3 ± 5.8	25.1 ± 0.5	0.04 ±0.01	-8.6 ± 0.7	240 ± 47	3.1 ± 0.6
Au_{20}-PEG_{5000} NP	19.7 ± 2.0	42.2 ± 4.0	39.6 ± 1.5	0.21 ± 0.00	-9.0 ± 0.6	2794 ± 365	2.2 ± 0.3

[a] Physical diameters of the gold core and gold core + PEG shell were measured by TEM with negative staining by EM Stainer. [b] Hydrodynamic diameters, polydispersity indice (PDI), and ζ-potentials were measured by dynamic light scattering (DLS) at room temperature (RT). PEG loading was quantified by the direct thiol displacement method of FAM-conjugated PEG strands (Method I). All reported data represent mean ± SD from three independent measurements.

Table S3. PEG loading determined by alternative methods to direct strand displacement

(A) **Method II:** Subtraction method by indirect measurement of excess thiolated PEG strands unattached to the gold core during synthesis of Au_x-PEG_y NP

NP	No. of PEG strand/NP	No. of PEG strand/nm^2
**Au_2-PEG_{200} NP**	116 ± 6.3	7.0 ± 0.5
**Au_3-PEG_{500} NP**	92 ± 9.0	2.5 ± 0.2
**Au_3-PEG_{500} -COOH NP**	99 ± 3.7	2.7± 0.1
**Au_5-PEG_{1000} NP**	260 ± 16.5	3.3 ± 0.2
**Au_5-PEG_{5000} NP**	258 ± 37	3.2 ± 0.4
**Au_{20}-PEG_{5000} NP**	3387± 358	2.8 ± 0.3

(B) **Method III:** Direct measurement of the organic content (PEG) of Au_x-PEG_y NP and Au_x-PEG_y-FA_{32} NP using TGA

NP	No. of PEG strand/NP	No. of PEG strand/nm^2
**Au_2-PEG_{200} NP**	125	7.5
**Au_3-PEG_{500} NP**	125	3.5
**Au_3-PEG_{500} -COOH NP**	138	3.8
**Au_3-PEG_{500}-FA_{32} NP**	56	1.5
**Au_5-PEG_{1000} NP**	274	3.5
**Au_5-PEG_{5000} NP**	214	2.7
**Au_{20}-PEG_{5000} NP**	3074	2.4

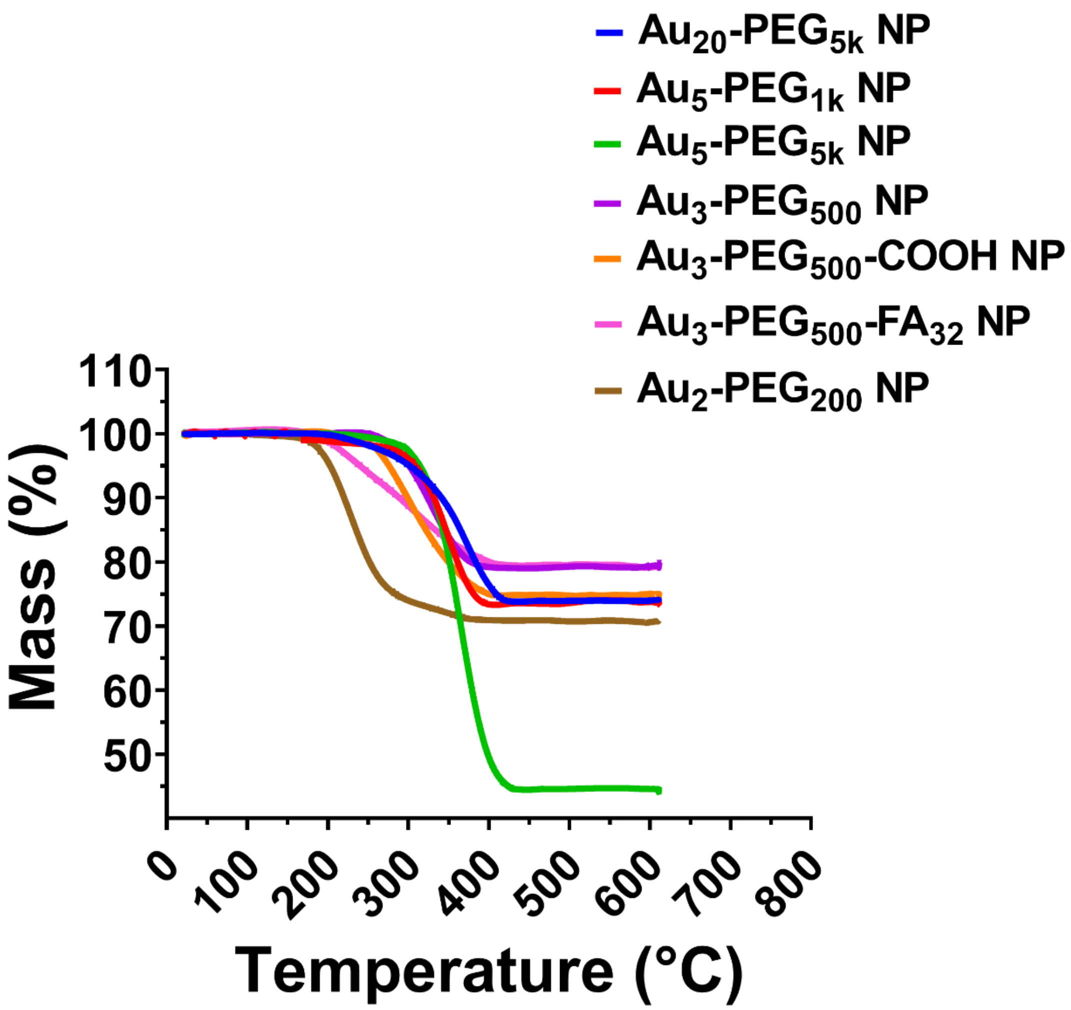

Fig. S5. TGA of the lost PEG contents during heating: Au_2-PEG_{200} (brown), Au_3-PEG_{500} (purple), Au_3-PEG_{500}-COOH (orange), Au_3-PEG_{500}-FA_{32} (pink), Au_5-PEG_{1000} (red), Au_5-PEG_{5000} (green), Au_{20}-PEG_{5000} NP (blue) NPs as a function of temperature under dry nitrogen gas.

Table S4. Hydrodynamic diameters of Au_x-PEG_y and Au_3-PEG_{500}-FA_z NPs upon incubation in water and 50% fetal bovine serum (FBS) in PBS at 37 °C for 24 h.

NP	Water		50% FBS in PBS	
	Hydrodynamic diameter (nm)	PDI	Hydrodynamic diameter (nm)	PDI
**Au_2-PEG_{200} NP**	2.8 ± 0.2	0.03 ± 0.02	5.4 ± 1.5	0.04 ± 0.03
**Au_3-PEG_{500} NP**	9.6 ± 0.2	0.01 ± 0.01	10.3 ± 1.3	0.62 ± 0.12
**Au_3-PEG-COOH NP**	9.5 ± 0.3	0.02 ± 0.02	10.2 ± 1.0	0.03 ± 0.03
**Au_3-PEG_{500}-FA_8 NP**	7.8 ± 0.3	0.03 ± 0.01	9.4 ± 1.3	0.23 ± 0.12
**Au_3-PEG_{500}-FA_{16} NP**	7.1 ± 0.3	0.04 ± 0.01	8.2 ± 0.2	0.04 ± 0.04
**Au_3-PEG_{500}-FA_{32} NP**	7.1 ± 1.4	0.13 ± 0.02	8.3 ± 1.6	0.51 ± 0.13
**Au_5-PEG_{1000} NP**	13.5 ± 1.8	0.02 ± 0.02	14.2 ± 3.8	0.62 ± 0.01
**Au_5-PEG_{5000} NP**	25.8 ± 0.9	0.04 ± 0.0	30.6 ± 2.3	0.53 ± 0.12
**Au_{20}-PEG_{5000} NP**	40.7 ± 0.8	0.22 ± 0.0	47.1 ± 5.8	0.62 ± 0.02

Hydrodynamic diameters were measured by dynamic light scattering. PDI = polydispersity index. All reported data represent mean ± SD from three independent measurements.

The hydrodynamic size of Au_3-PEG_{500}-FA_{32} NP is slightly smaller than Au_3-PEG_{500} NP even with the addition of FA on the PEG strands, possibly because the density of PEG strands on the NPs may affect the overall hydrodynamic diameters. As there are more PEG strands on Au_3-PEG_{500} NPs than the Au_3-PEG_{500}-FA_{32} NPs (81 vs. 32 strands), it is reasonable that Au_3-PEG_{500}-FA_{32} NPs appear slightly smaller than Au_3-PEG_{500} NPs.

By DLS measurements, the hydrodynamic sizes of Au_x-PEG_y and Au_3-PEG_{500}-FA_z NPs are slightly larger after exposure to serum-containing medium (50% FBS in PBS) at 37 °C for 24 h. For example, Au_{20}-PEG_{5000} NPs became slightly enlarged from 40.7 ± 0.8 nm to 47.1 ± 5.8 nm. DLS measurements do not reveal drastic increase in hydrodynamic size after serum incubation, proof of colloidal stability. We did not test the NPs in 100% FBS because high concentrations of serum proteins cause intense light scattering and interfere with the DLS measurements.

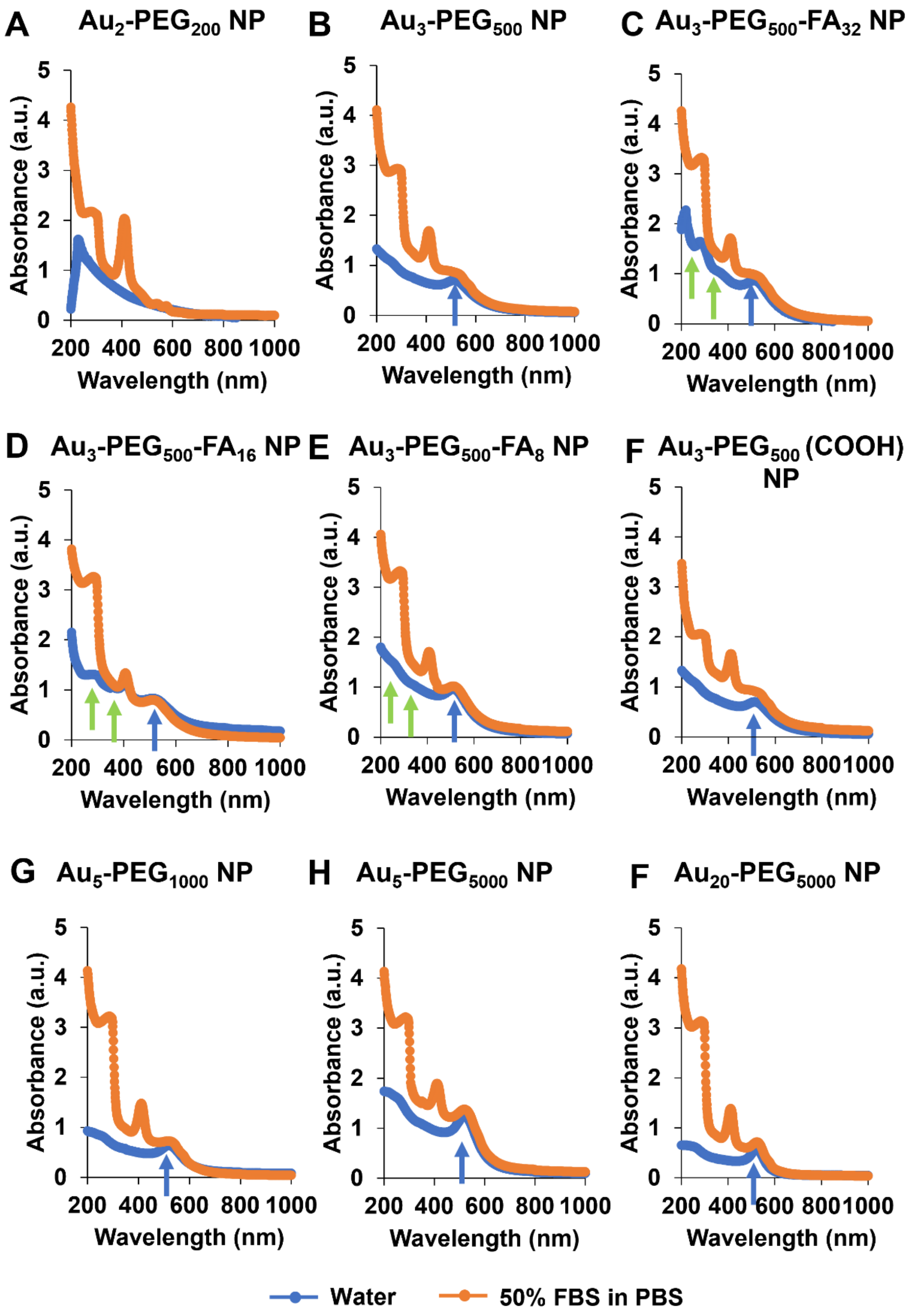

Fig. S6. UV-Vis spectra of Au_x-PEG_y and Au_3-PEG_{500}-FA_y NPs after incubation in water and 50% fetal bovine serum (FBS) in PBS at 37°C for 24 h. Blue arrows: Localized surface plasmon resonance (LSPR) peaks of NPs. Green arrows: peak and shoulder of FA. We confirmed the colloidal stability of Au_x-PEG_y and Au_3-PEG_{500}-FA_{32} NPs in 50% FBS. (A) The LSPR peak of Au_2-PEG_{200} NPs is not detectable by UV-vis spectrophotometry. (B)-(F) For x > 3 nm, the LSPR peak of Au_x-PEG_y and Au_3-PEG_{500}-FA_{32} NPs at 508–520 nm does not change drastically and that no peak of AuNP aggregation in the 600–1000 nm range is observed upon incubation in serum. (C) The absorption peak of FA at 280 nm and shoulders of FA at 300 and 370 nm can be detected for the Au_3-PEG_{500}-FA_{32} NP sample in water.

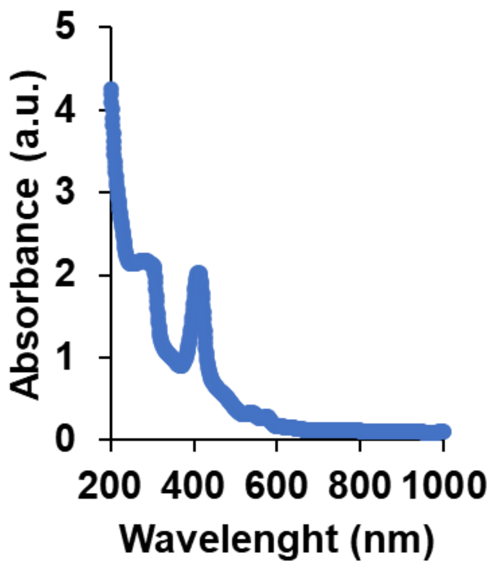

Fig. S7. UV-Vis spectra of 50% FBS in PBS without the addition of gold NPs. Strong protein peaks at ~280 and ~400 nm could mask the FA peak at 300 nm and shoulder at 370 nm when Au_3-PEG_{500}-FA_{32} NPs were incubated in 50% FBS (Fig. S6).

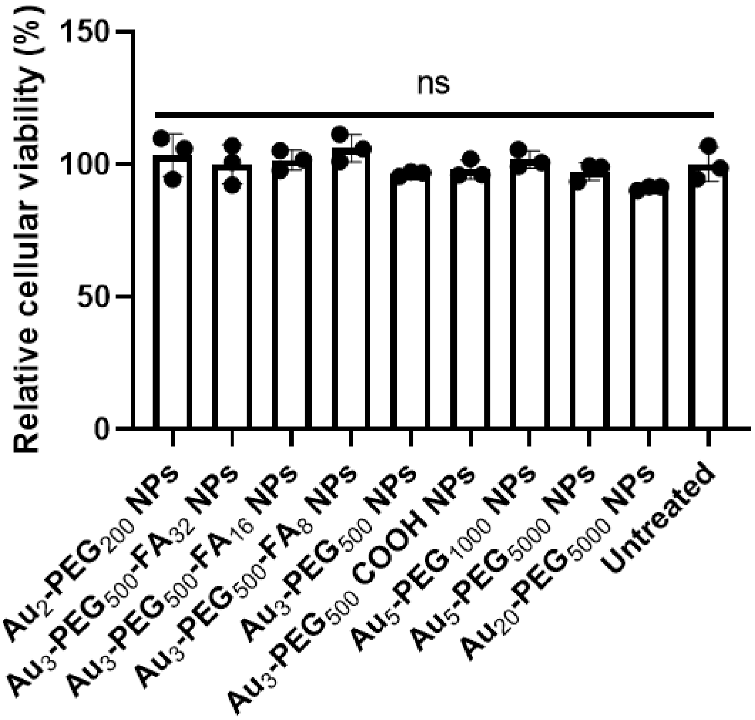

Fig. S8. Cytotoxicity of Au_x-PEG_y and Au_3-PEG_{500}-FA_y NPs. Primary kidney tubule cells harvested from healthy Balb/c mice were incubated with 100 mM NPs in complete culture medium for 24 h. By using alamarBlue assay, the treated cells remained largely viable after incubation with all types of NPs tested. Data are presented as mean ± SD. Statistical significance was calculated by one-way ANOVA with Turkey's Test for post-hoc analysis. ns: not significant ($p > 0.05$). n= 3 per group, across 1 experiment.

Organ-level distribution of Au_x-PEG_y and Au_3-PEG_{500}-FA_{32} NPs in UUO mice

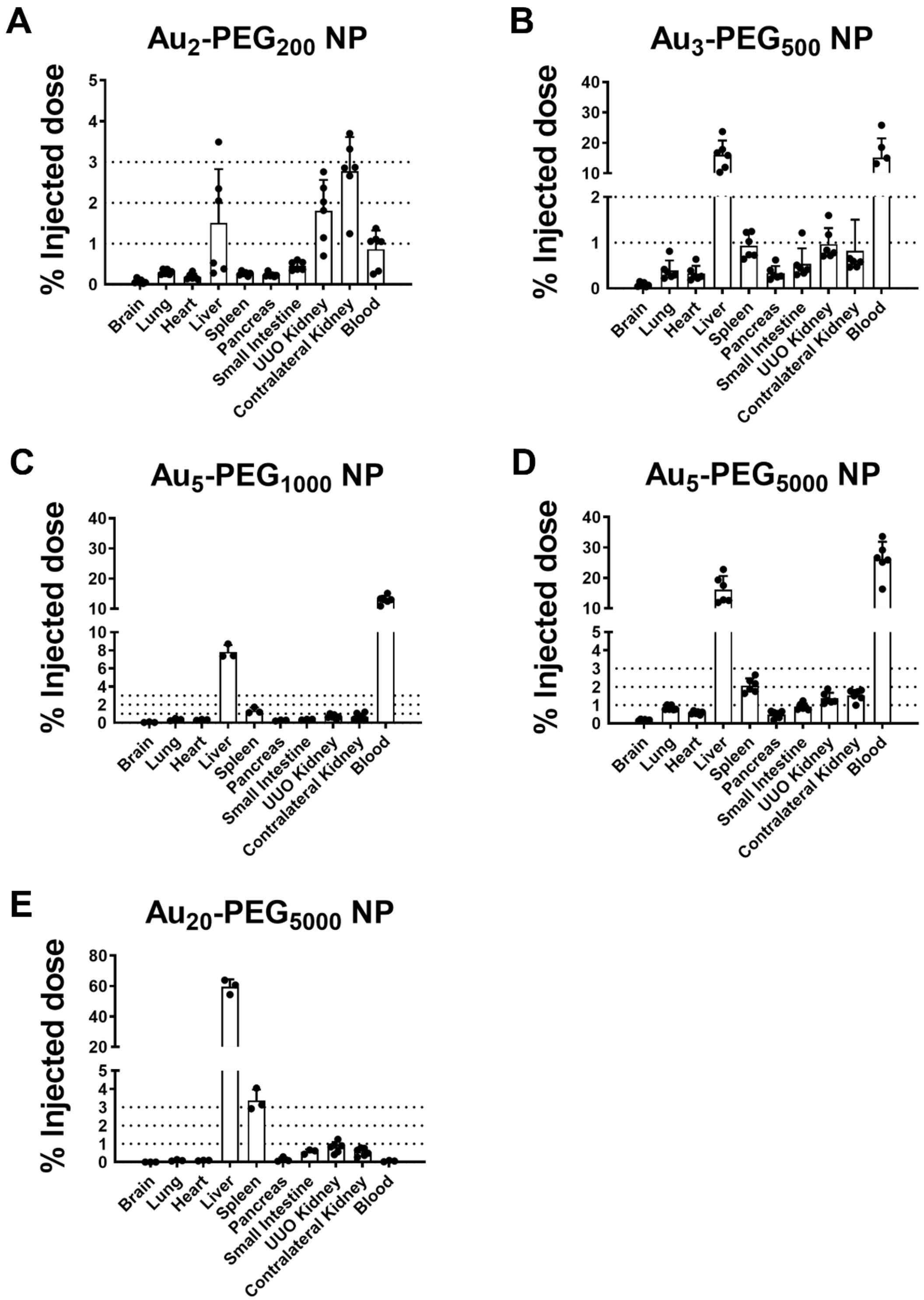

Fig. S9. Organ-level distribution for Au_x-PEG_y NPs. The NPs (100 µg of Au) were IV injected into UUO mice via the tail vein on Day 7 post-UUO surgery. 24 h post-injection, the mice were sacrificed for organ collection. The bulk gold content in each organ was detected using ICP-MS. Error bar denotes ± 1 SD. Data are from n =3-6, across 1 experiment.

Renal and hepatobiliary clearance of NPs

Excretion was measured by collecting urine and feces at various time points post-NP injection. As the UUO kidney was ligated, gold detected in the urine were cleared from the CL kidney. Gold detected in the feces were cleared through the liver, into the bile and excreted as feces.

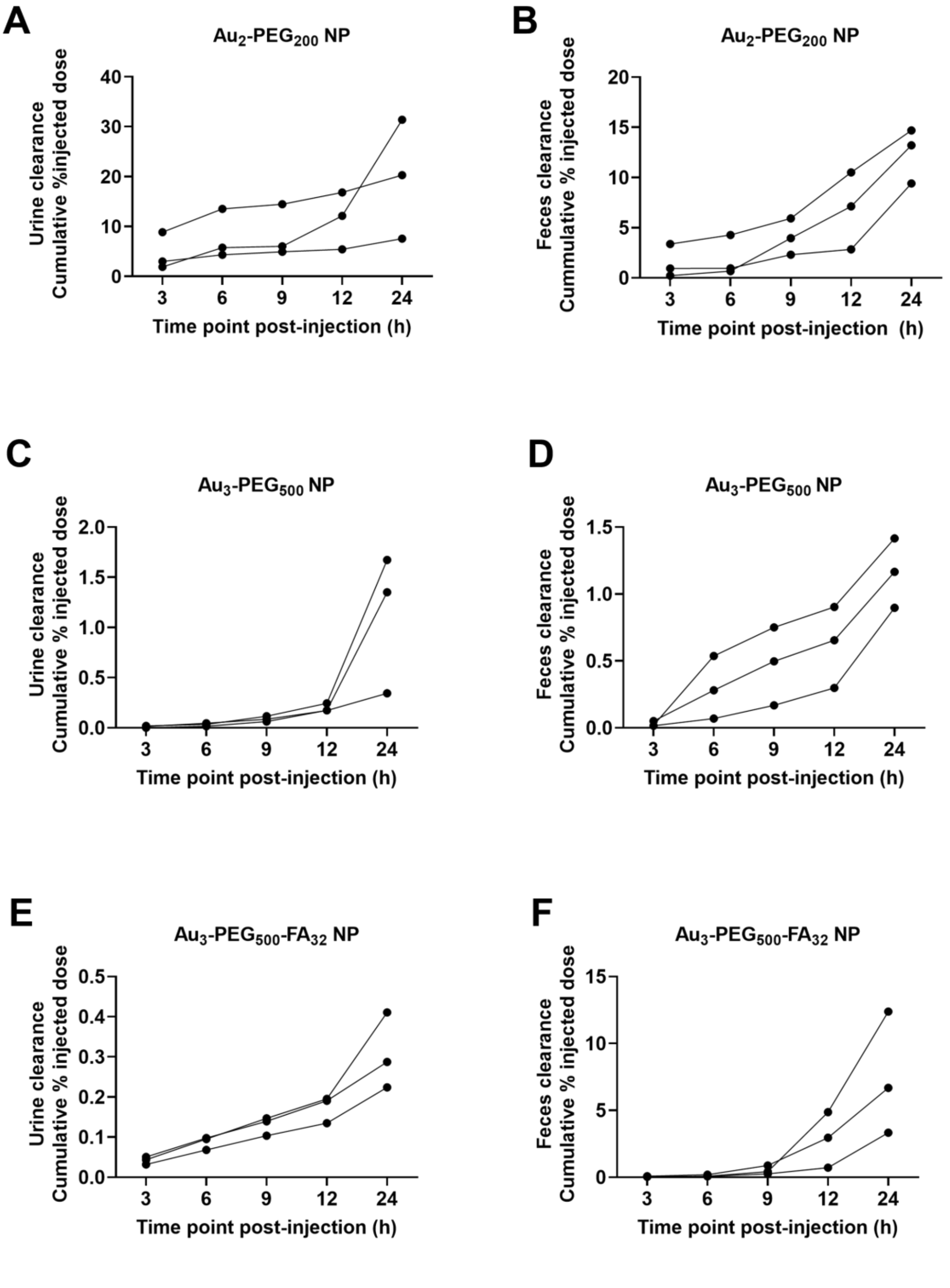

Fig. S10. Excretion of (A–B) Au_2-PEG_{200}, (C–D) Au_3-PEG_{500}, and (E–F) Au_3-PEG_{500}-FA_{32} NPs by renal clearance and hepatobiliary clearance in UUO mice. The Au content in the collected urine indicates clearance from the contralateral kidney only. Data are from n =3, across 1 experiment. Each line indicates the cumulative kinetics data from one animal.

For renal clearance, Au_2-PEG_{200} NPs were cleared most efficiently, with ~4.5 %ID being excreted in the first 3 h post-injection and 19.7 %ID 24 h post-injection. By contrast, the amounts of renal clearance of Au_3-PEG_{500} and Au_3-PEG_{500}-FA_{32} NPs 24 h post-injection were 1.12 %ID and 0.31 %ID, respectively. The amount of Au_3-PEG_{500}-FA NPs detected in the urine was lower than Au_3-PEG_{500} NPs, probably because some Au_3-PEG_{500}-FA_{32} NPs that could pass through the GFB were reabsorbed by tubular cells.

For hepatobiliary clearance, 12.4 %ID of Au_2-PEG_{200} NPs could be detected in the feces 24 h post-injection, indicating that a large portion of NPs traversed the liver despite only ~1.5 %ID of retention in the liver 24 h post-injection. The cumulative excreted amounts in feces for Au_3-PEG_{500} and Au_3-PEG_{500}-FA_{32} NPs 24 h post-injection were 1.2 %ID and 7.5 %ID, respectively, suggesting that Au_3-PEG_{500}-FA_{32} NPs can be cleared more easily than Au_3-PEG_{500} NPs by the hepatobiliary clearance pathway.

Lack of aqueous solubility of Au_2-PEG_{200}-FA NPs

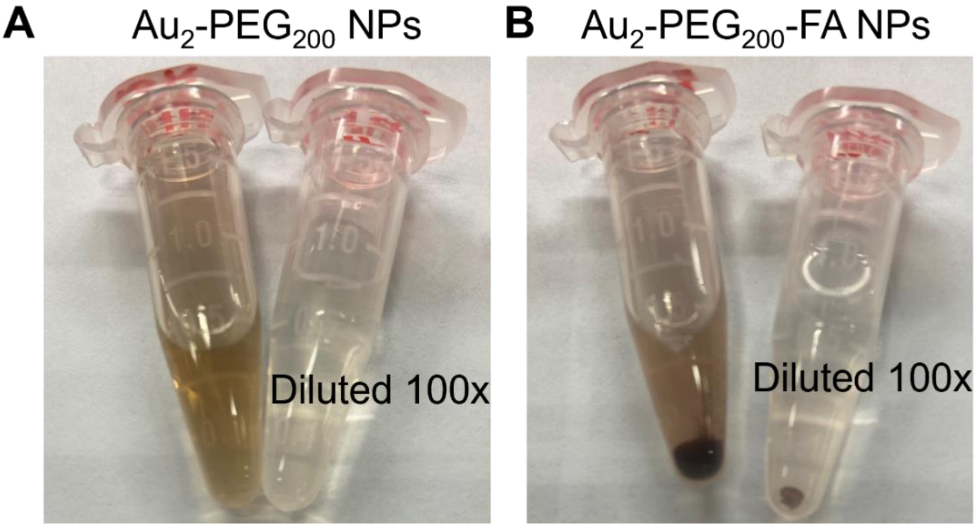

Fig. S11. Photographs of (A) Au_2-PEG_{200} and (B) Au_2-PEG_{200}-FA NPs after incubation in water for 24 h. The Au_2-PEG_{200}-FA NPs are not soluble in water, forming aggregates that sediment to the bottom of the microcentrifuge tube by gravity. No further studies on characterizing the Au_2-PEG_{200}-FA NPs were performed due to their tendency to aggregate.

Disease stages of UUO mice

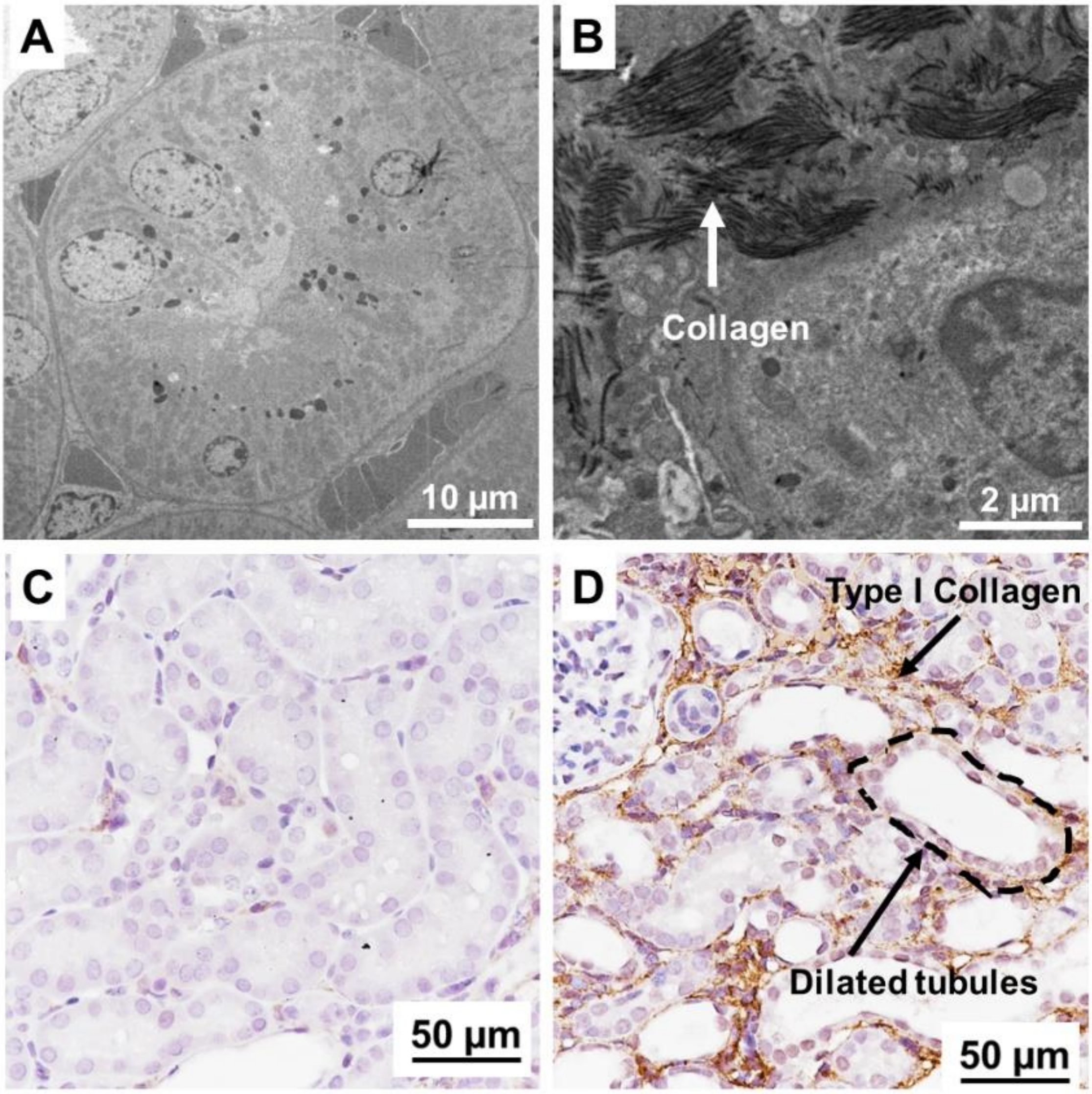

Fig. S12. Structural and histological features of a normal kidney and unilateral ureteral obstructed (UUO) kidney. (A) Transmission electron microscopy (TEM) image of a healthy proximal tubule. (B) TEM image of a fibrotic area with bundles of collagen fiber in the interstitium (arrow). (C&D) Representative images of immunohistochemical staining for type I collagen (brown) in (C) normal kidney and (D) UUO kidney. Expression of type I collagen in (D) the UUO kidney is pronounced (brown), while its expression in the healthy kidney is minimal.

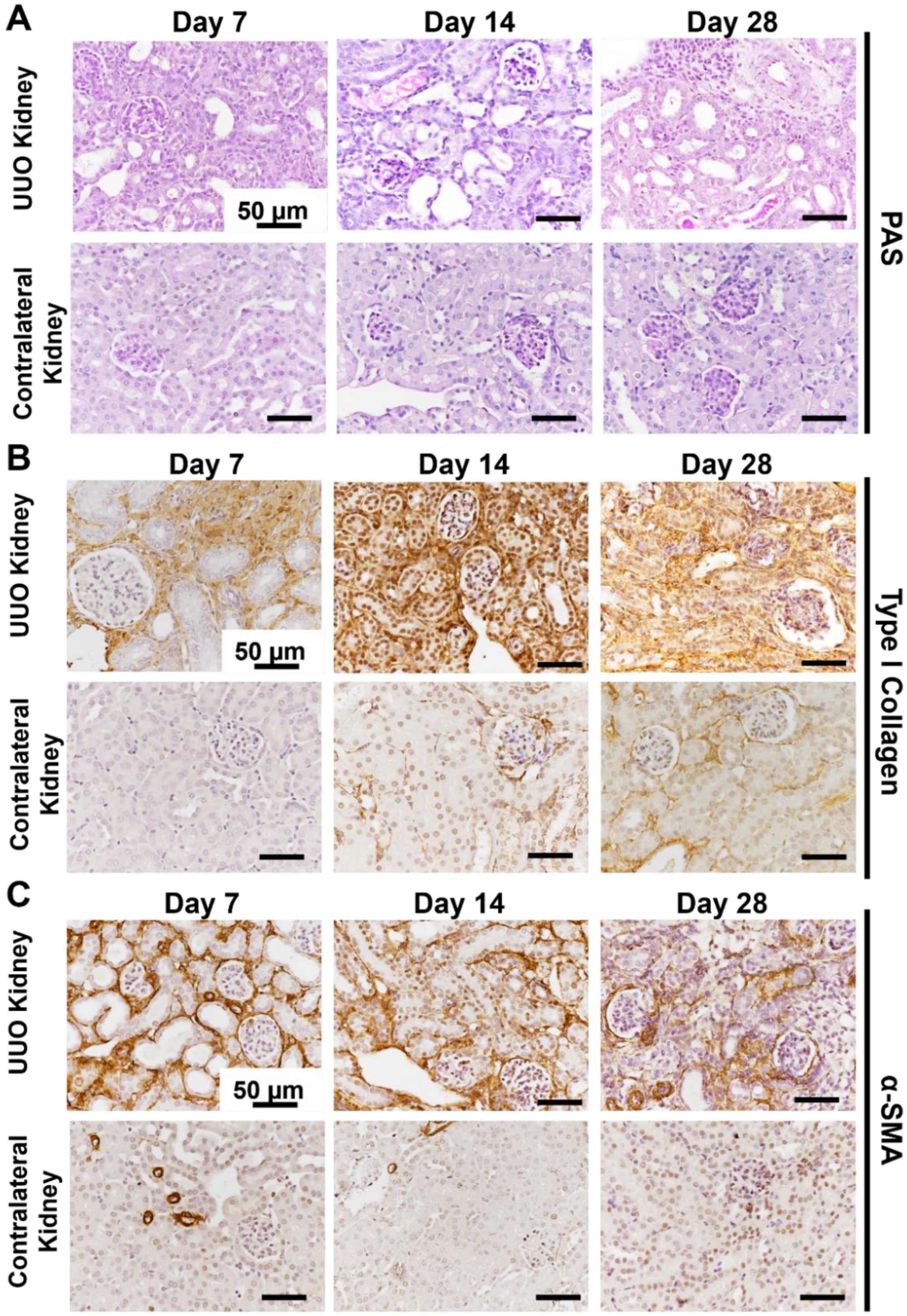

Fig. S13. Periodic acid–Schiff (PAS) and immunohistochemistry (IHC) staining of kidney sections from UUO mice of different disease stages. (A) PAS staining, (B) IHC staining of type I collagen, and (C) α-smooth muscle actin (α-SMA) in UUO and CL kidneys from mice on Day 7, Day 14, and Day 28 post-UUO surgery. PAS and IHC staining of the kidney sections showed progressive degeneration of renal tubules. In the CL kidney, the deposition of type I collagen was only evident on Day 28 post-UUO surgery, maybe due to the increased burden on the CL kidney in response to loss of function of the UUO kidney. In the CL kidney, α-SMA expression were restricted to the wall of arterioles. These results are consistent with the reported outcome of UUO surgery (37–39) and confirm the establishment of tubulointerstitial fibrosis in mice.

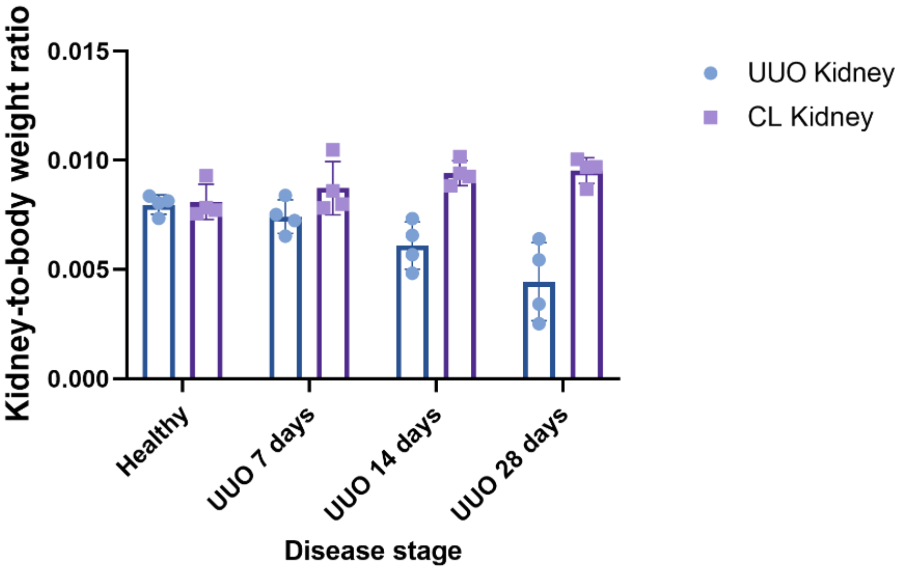

Fig. S14. Kidney-to-body weight ratio as a function of disease stage of tubulointerstitial fibrosis. Kidney weight was normalized to the body weight of each mouse at sacrifice. Error bar denotes ± 1 SD, with n = 4 (across 1 experiment). The severity of fibrosis was assessed on Day 7, Day 14, and Day 28 post UUO surgery. As fibrosis progresses, the weight of UUO kidney decreases but the weight of the contralateral (CL) kidney increases.

Combined effects of NP size and disease stage on NP distribution to the UUO kidney

For Au_3-PEG_{500}, Au_5-PEG_{1000}, and Au_5-PEG_{5000} NPs, accumulation in the UUO kidney was significantly higher when the NPs were injected on Day 7 post-UUO surgery than when injected on Day 28 post-UUO surgery. For example, accumulation of Au_3-PEG_{500} NPs in the UUO kidney was 2.8-fold lower when the NPs were injected on UUO Day 28 (2.0 %ID/g) as compared to UUO Day 7 (5.7 %ID/g) (Fig. S15a). As the data were normalized based on the kidney weight, this result may stem from the difficult entry of NPs to the UUO kidney due to increased tubule pressure. For Au_{20}-PEG_{5000} NPs, there was no significant variation in their accumulation in the UUO kidney across all disease stages tested (4.9 %ID/g for UUO Day 7, 4.5 %ID/g for UUO Day 14, and 4.4 %ID/g for UUO Day 28; Fig. S15d). These results indicate that NP size and UUO disease stage are both critical parameters for delivery to the UUO kidney.

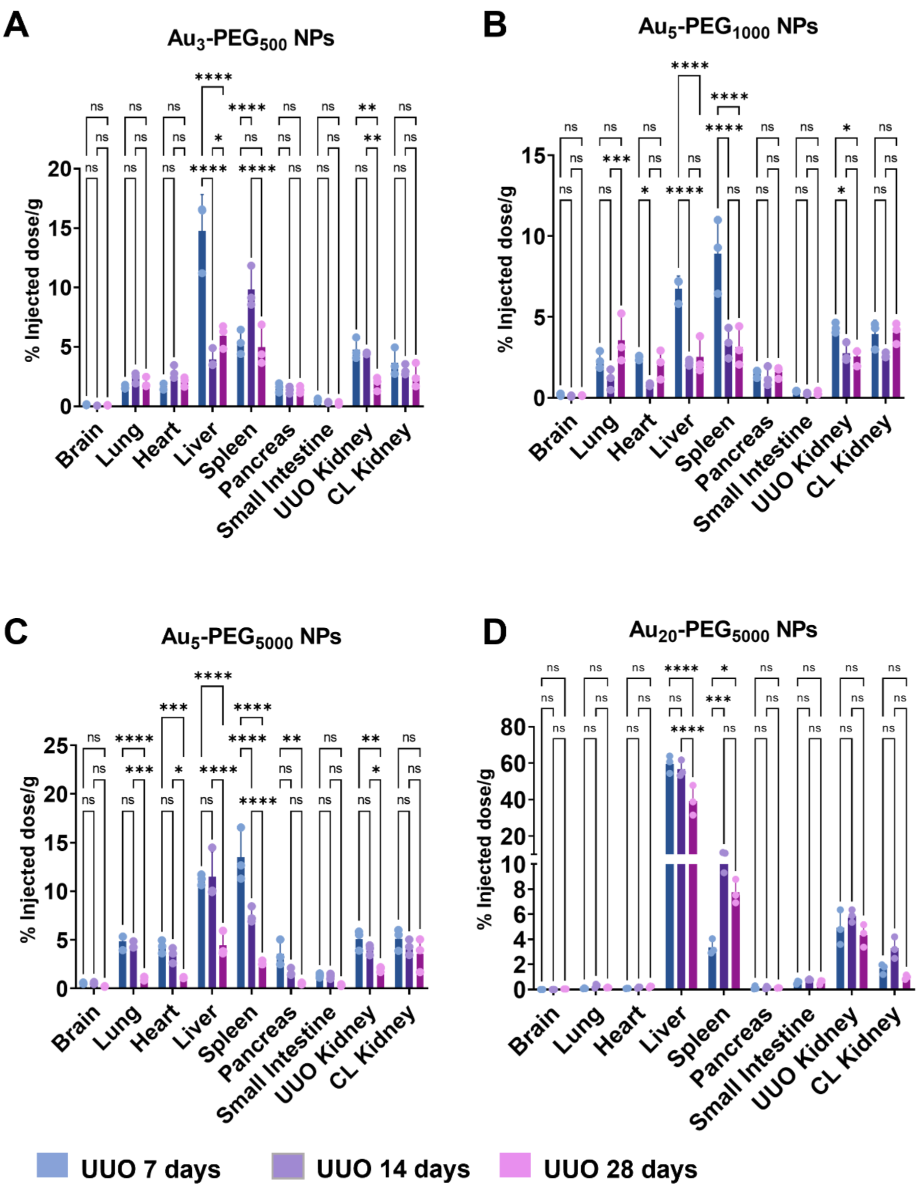

Fig. S15. Organ-level distribution of Au_x-PEG_y NPs in UUO mice as a function of disease stage. (A) Au_3-PEG_{500} NPs, (B) Au_5-PEG_{1000} NPs, (C) Au_5-PEG_{5000} NPs, and (D) Au_{20}-PEG_{5000} NPs were injected into mice via the tail-vein on Day 7, 14 or 28 post-UUO surgery. 24 h post-injection, the mice were sacrificed for organ collection. Statistical significance was evaluated using One-Way ANOVA and Tukey's multiple comparisons test. * = $p \leq 0.05$, ** = $p \leq 0.01$,*** = $p \leq 0.001$ **** = $p \leq 0.0001$, ns = not significant. Error bar denotes ± 1 SD, with n = 3 across 1 experiment.

Combined effects of NP size and disease stage on blood circulation

Of all NP sizes studied, the blood concentration of Au_{20}-PEG_{5000} NPs was the lowest 24 h post-injection (Fig. S16) owing to their most severe clearance from the liver and spleen (Fig. S15D) for all disease stages tested. The blood concentration of Au_5-PEG_{5000} NPs was the highest 24 h post-injection because they were neither severely cleared by the liver and spleen nor renally cleared (Fig. S15C), for all disease stages tested. The blood concentrations of Au_3-PEG_{500} and Au_5-PEG_{1000} NPs were at intermediate levels because they could cross the glomerular filtration barrier but were not severely cleared by the liver and spleen (Fig. S15A, B). These data agree with past reports that NPs with smaller sizes or longer PEG lengths have longer circulation (40),(41). No effect of disease stage on blood circulation was observed across all NP sizes.

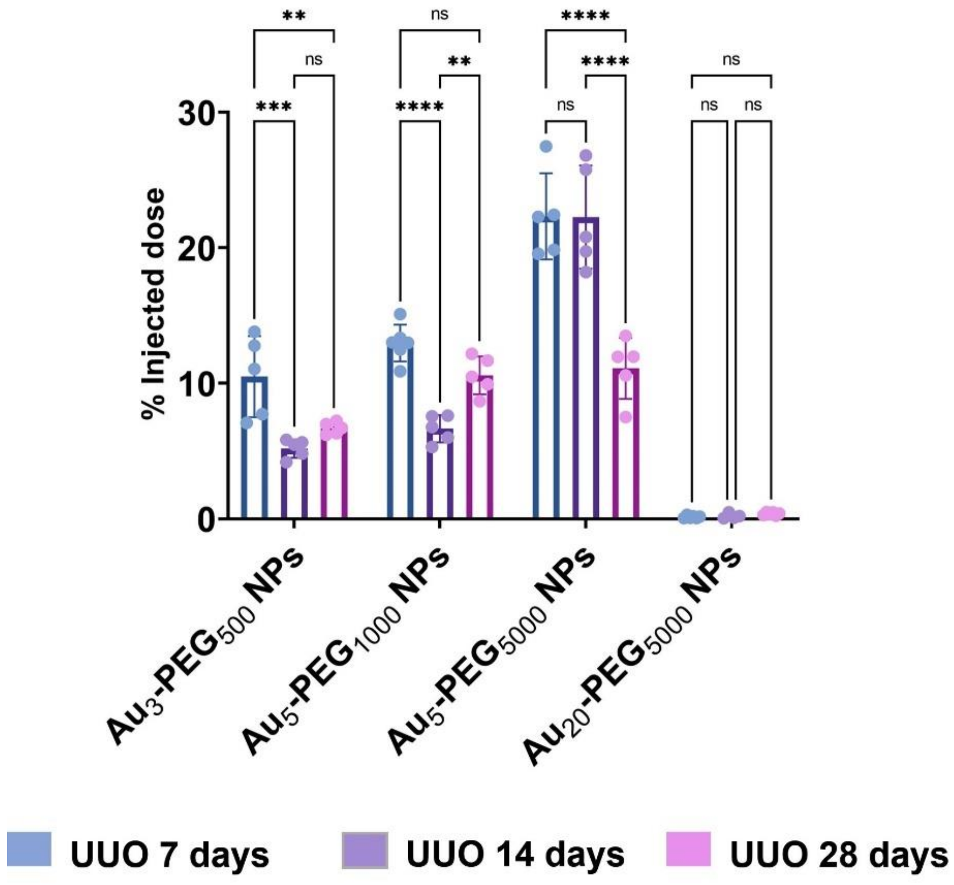

Fig. S16. Blood concentration of Au_x-PEG_y NPs in UUO mice 24 h post-injection. NPs were injected into mice via the tail-vein on Day 7, 14 or 28 post-UUO surgery. Statistical significance was evaluated using One-Way ANOVA and Tukey's multiple comparisons test. * = $p \leq 0.05$, ** = $p \leq 0.01$, *** = $p \leq 0.001$, **** = $p \leq 0.0001$, ns = not significant. Error bar denotes ±1 SD, with n = 3–6 across 2 experiments.

Profile of serum proteins (from blood of UUO mice) adsorbed to NPs

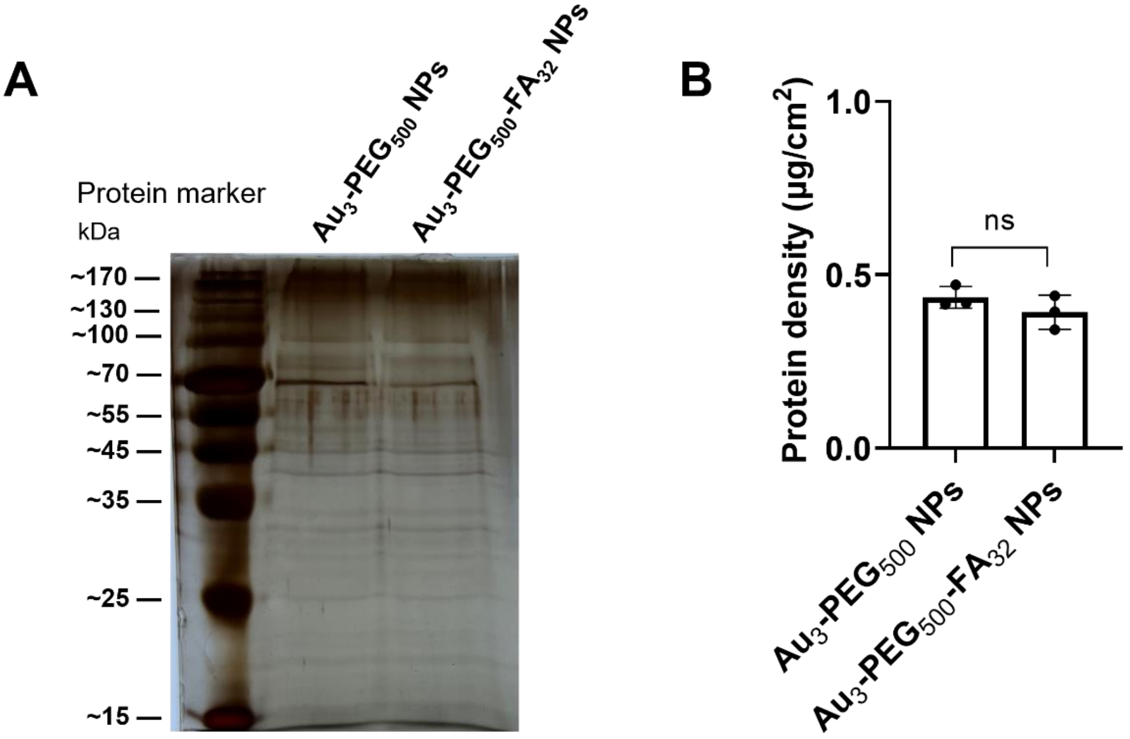

C

Au_3-PEG_{500} NPs		
Symbol	MW (kDa)	% (w/w)
Alb	68.692	53.67
Ighm	49.971	6.02
Prss1	26.134	5.54
Tf	76.723	5.21
Apoa1	30.684	4.32
Serpina3k	46.879	3.20
A2m	165.85	2.03
C3	186.48	1.67
Gm5409	26.531	1.31
Krt78	54.764	1.19
Hpx	51.317	1.12
Serpina1c	45.823	0.98
Apoa2	11.319	0.90
Apoe	35.847	0.53
Kng1	73.1	0.50
Ahsg	37.325	0.50
Cfh	141.27	0.49
Igkc	24.165	0.43
Cd5l	38.862	0.42
Hbat1	15.085	0.41

Au_3-PEG_{500}-FA_{32} NPs		
Symbol	MW (kDa)	% (w/w)
Alb	68.692	38.77
Apoa1	30.684	6.84
Tf	76.723	6.77
A2m	165.85	5.14
Ighm	49.971	4.38
Serpina3k	46.879	3.61
Hpx	51.317	2.45
Apoe	35.847	2.42
C3	186.48	1.92
Apob	509.43	1.87
Serpina1c	45.823	1.71
Prss1	26.134	1.24
Cfh	141.27	1.19
Ces1c	61.055	0.99
Mug1	165.3	0.97
Igkc	24.165	0.92
Dnah17	509.03	0.92
Apoc3	10.922	0.91
Apoa4	45.029	0.84
Kng1	73.1	0.74

Fig. S17. Protein composition of Au_3-PEG_{500} and Au_3-PEG_{500}-FA_{32} NP upon incubation in blood serum of UUO mice (Day 7 post-UUO surgery) at 37 °C for 1 h. (A) Polyacrylamide gel electrophoresis (PAGE) analysis of adsorbed proteins after serum incubation. (B) By the bicinchoninic acid (BCA) assay, the densities of adsorbed serum proteins on Au_3-PEG_{500} and Au_3-PEG_{500}-FA_{32} NPs were not statistically significant (Mann-Whitney test). Data are presented as ± SD, with n = 3 samples per group. (C) The 20 most abundant proteins adsorbed to Au_3-PEG_{500} NP and Au_3-PEG_{500}-FA_{32} NP as detected by label-free liquid chromatograph-tandem mass spectrometry (LC−MS/MS) in triplicates. Relative abundance (%)

represents the weight percentage of each constituent protein. Proteins are represented by MGI symbols. (A2m) Alpha-2-macroglobulin; (Ahsg) Alpha-2-HS-glycoprotein; (Alb) Albumin; (Apoa1) Apolipoprotein A-I; (Apoa2) Apolipoprotein A-II; (Apoa4) Apolipoprotein A-IV; (Apob) Apolipoprotein B-100; (Apoc3) Apolipoprotein C-III; (Apoe) Apolipoprotein E; (C3) Complement C3; (Cd5l) CD5 antigen-like; (Ces1c) Carboxylesterase 1C; (Cfh) Complement factor H; (Dnah17) Dynein heavy chain 17; (Gm5409) Try10-like trypsinogen; (Hbat1) Hemoglobin subunit alpha; (Hpx) Hemopexin; (Ighm) Ig mu chain C region; (Igkc) If kappa light chain; (Kng1) Kininogen-1; (Krt78) Krt78 protein; (Mug1) Murinoglobulin-1; (Prss1) Protease, serine 1; (Serpina1c) Alpha-1-antitrypsin 1-3; (Serpina3k) Serine protease inhibitor A3K; (Tf) Serotransferrin.

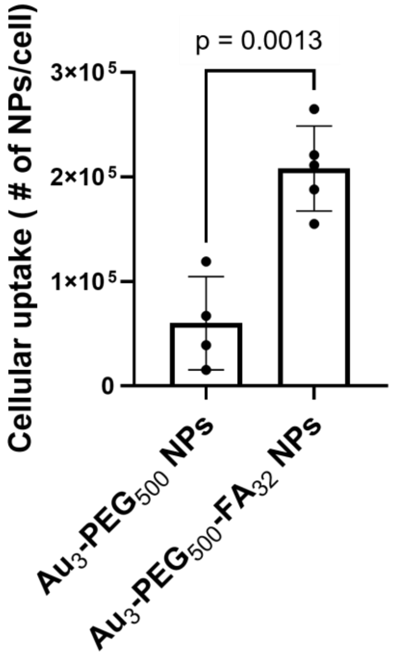

Fig. S18. In vitro association of Au_3-PEG_{500} and Au_3-PEG_{500}-FA_{32} NPs with primary tubule cells isolated from the kidneys of healthy Balb/c mice upon incubation in NP-containing medium (100 nM NPs formulated in complete RPMI) for 2 h. Statistical significance was evaluated using Mann-Whitney test. ** = p ≤0.01, *** = p ≤0.001, **** = p ≤0.0001, ns = not significant. Error bar denotes ±1 SD, with n = 5 across 1 experiment.

A

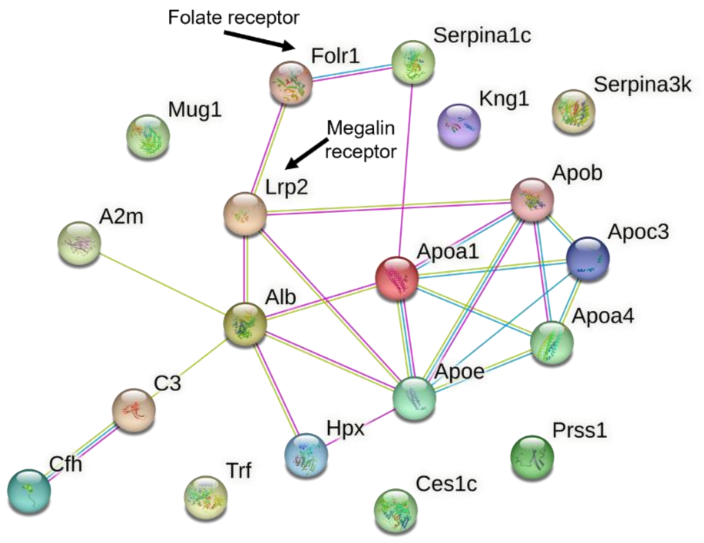

B

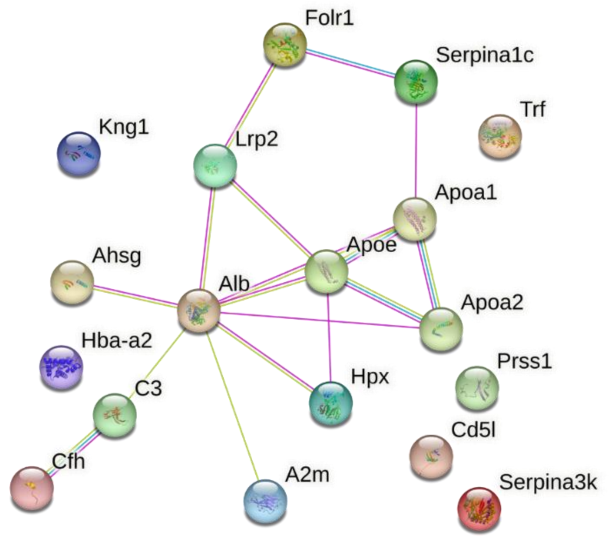

C

Serum protein	Receptor	STRING: interaction score
Alb	Lrp2	0.895
Apob	Lrp2	0.742
Apope	Lrp2	0.621
Serpina1c	Folr1	0.635
Folr1	Lrp2	0.588

Fig. S19. Search Tool for the Retrieval of Interacting Genes/Proteins (STRING) physical subnetwork shows the interactions between the two important renal tubule receptors relevant to this work (folate receptor FOLR1 and megalin LRP2) and the top 20 most abundant serum proteins bound to (A) Au$_3$-PEG$_{500}$-FA$_{32}$ and (B) Au$_3$-PEG$_{500}$ NPs upon incubation in serum collected from UUO mice for 1 h. Network nodes represent proteins produced by a single, protein-coding gene locus. The edges present protein-protein associations. Blue line indicates known interactions from curated databases, purple line indicates known interactions that were experimentally determined, and yellow line indicates interactions determined from text-mining (co-mentioned in PubMed abstract). The NP-adsorbed serum proteins interact with LRP2 more favorably than FOLR1. (C) Predicted interaction score of the proteins and receptors. Network images and interaction score were exported from STRING analysis on February 1, 2023. An interaction score of 0.895 means that there is an 89.5 % chance that the interaction is true.

Tissue-level distribution of Au_3-PEG_{500} and Au_3-PEG_{500}-FA_{32} NPs in the UUO kidney

Au_3-PEG_{500}-FA_{32} NPs most likely cross the glomerular filtration barrier (GFB) for entering renal tubules rather than leaving peritubular capillaries to go to interstitial space and subsequently enter renal tubules. Based on literature precedent, expression of FR is mostly found on the apical side of the renal proximal tubule cells, rather than the basal side (42).

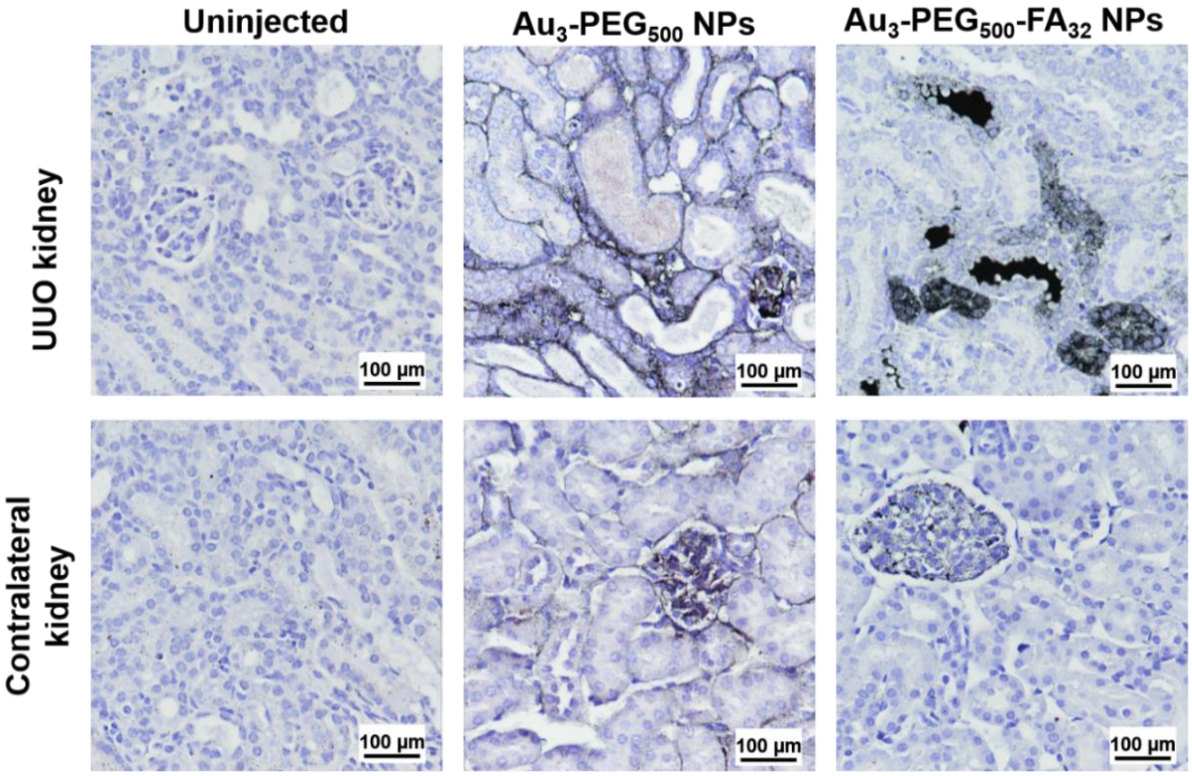

Fig. S20. Representative light micrographs of silver-enhanced UUO kidney sections show localization of Au_3-PEG_{500} NPs (middle column) and Au_3-PEG_{500}-FA_{32} NPs (right column) in the renal cortex. Representative images are from 3 kidney sections from each mouse, n = 3 mice/group, across 1 experiment. On Day 7 post-UUO surgery, the mice were i.v. injected with the NPs. 24 h post-injection, mice were sacrificed, and the UUO kidney was harvested, sectioned, silver-enhanced, and stained histologically. When using the commercial kit for silver enhancement, we followed the manufacturer's protocol to ensure that the brown, dark grey, or black stains originated from the silver-enhanced gold NPs that accumulated in the kidney after intravenous injection into mice. We included a negative control (kidney section from uninjected mice) that showed the lack of positive silver stains in the section (left column), ensuring the specificity of the silver stain protocol for gold NPs.

The extra silver-enhanced histological images of the UUO kidney below show that some Au_3-PEG_{500}-FA_{32} NPs are inside the lumen of the tubule cells and others are inside the tubule cells. These images provide a large-area snapshot of the transport of NPs through the urinary system at the time of sacrifice, suggesting that the NP are flowing through the tubules after their glomerular filtration. The images further strengthen our claim that Au_3-PEG_{500}-FA_{32} NPs mainly accumulated in some tubule cells or were found inside the tubule lumen.

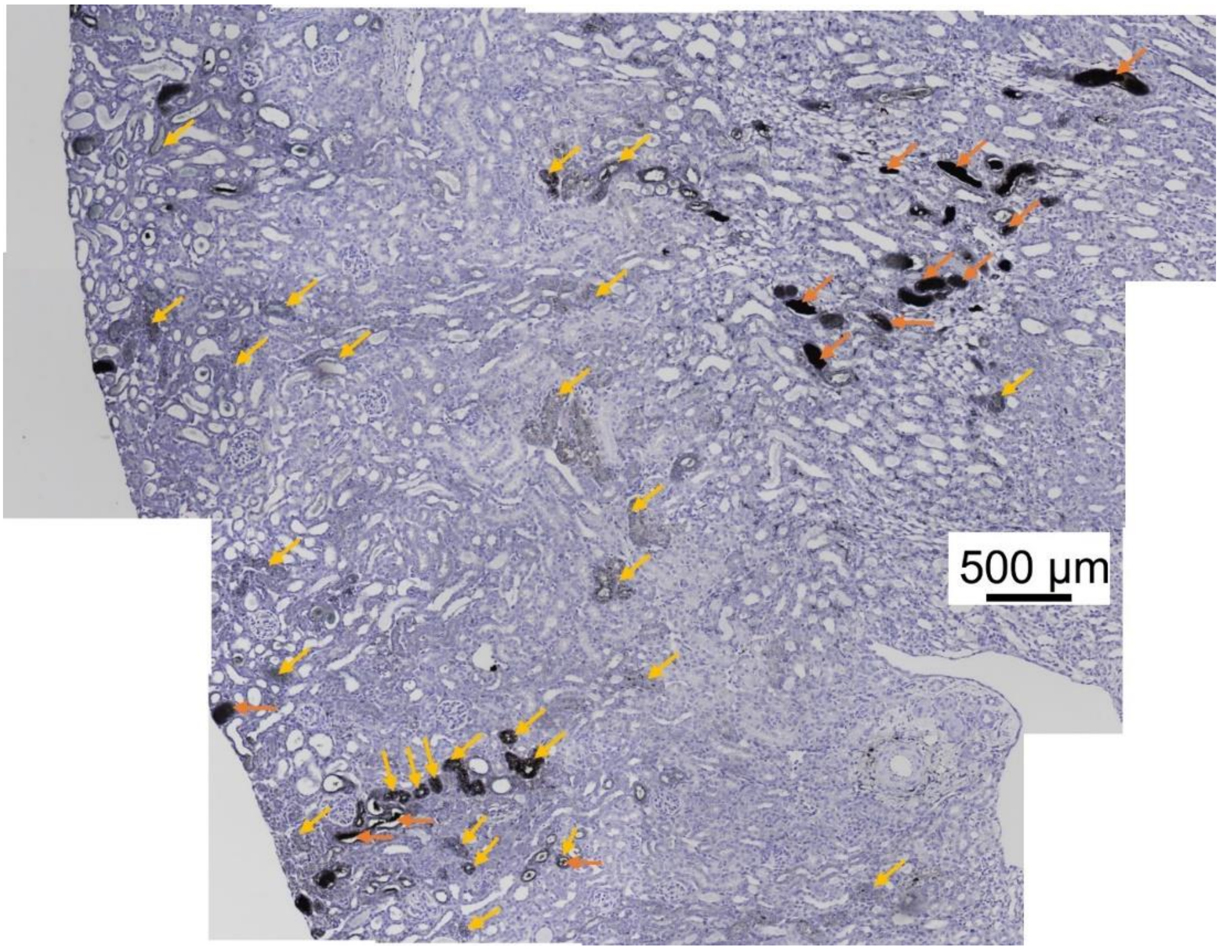

Fig. S21. Additional light micrographs of silver-enhanced UUO kidney sections, when optically stitched together, provide a large-area overview of the localization of Au_3-PEG_{500}-FA_{32} NPs in the lumen of tubule cells (orange arrows) and inside tubule cells (yellow arrows).

For Au_3-PEG_{500} NPs, it is challenging to pinpoint the exact route of delivery to the UUO kidney. The upper limit of the pore size of peritubular capillaries is ~6 nm by TEM imaging (43). Since TEM measurements involve dry samples, the hydrodynamic size cutoff would be slightly larger. The hydrodynamic size cutoff for glomerular filtration is ~10 nm for soft biomolecules (44). So, both barriers have similar cutoff sizes. Here, Au_3-PEG_{500} NPs are ~9 nm in hydrodynamic size, so they may adopt both routes for entering renal tubules, (1) crossing the glomerular filtration barrier to reach the tubule lumen and then entering the tubule cells or (2) extravasating from the peritubular capillaries, entering the interstitial space, and then finally entering the tubules.

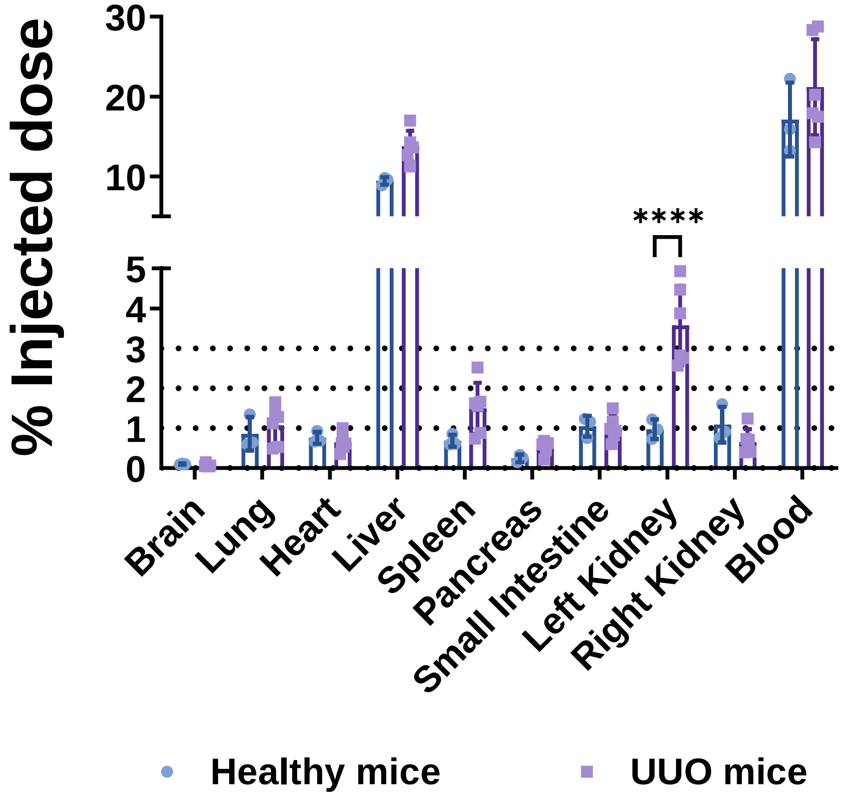

Fig. S22. Organ-level distribution of Au_3-PEG_{500}-FA_{32} NPs 24 h post-injection into healthy control Balb/c mice or UUO mice on 7 d after UUO surgery. For UUO mice, the “left kidney” represents the UUO kidney, and the “right kidney” label represents the contralateral (CL) kidney. Statistical significance was evaluated using student t- test for comparison of left kidney distribution in the healthy and UUO mice. **** = $p \leq 0.0001$. Error bar denotes ±1 SD, with n = 3–6 across 2 experiments.

Cellular-level distribution of Au_3-PEG_{500} and Au_3-PEG_{500}-FA_{32} NPs in the UUO kidney

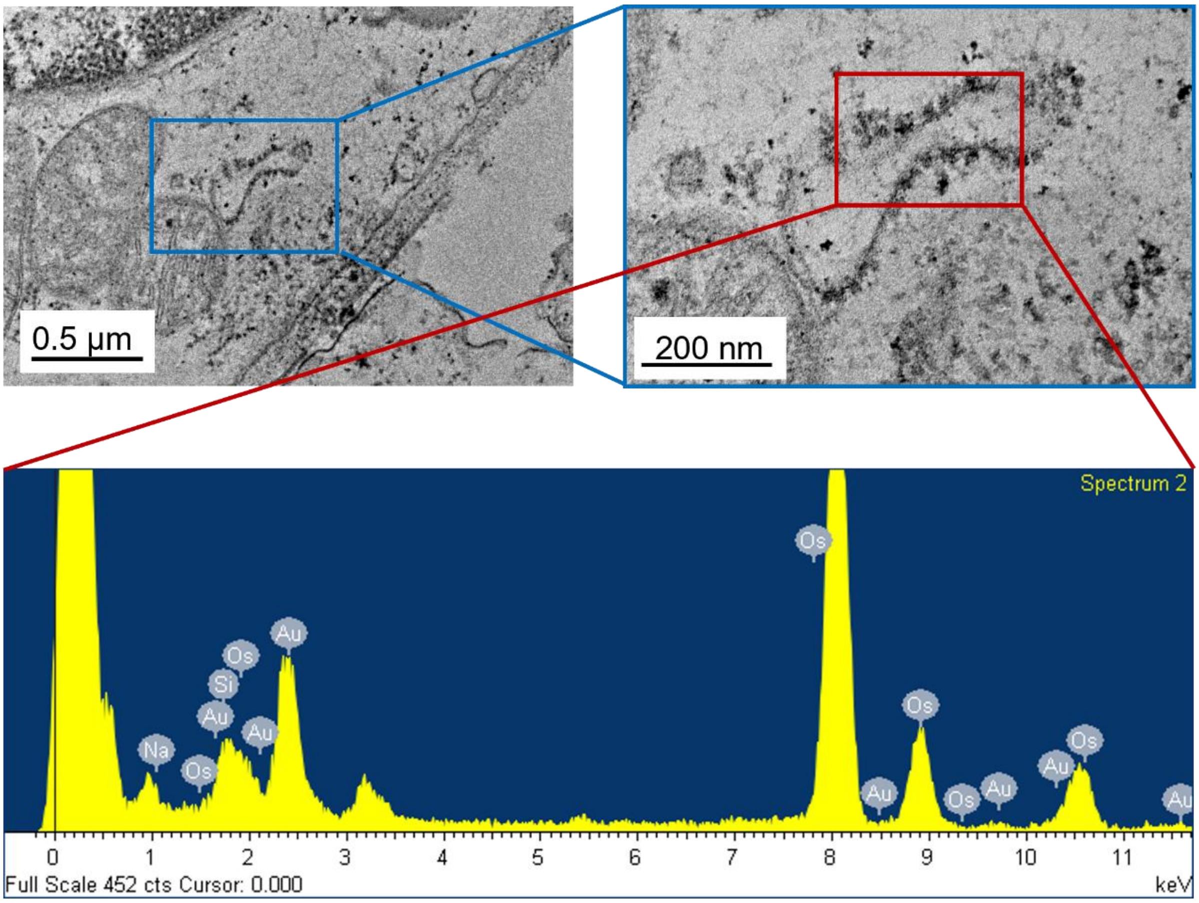

Fig. S23. Representative TEM images show the presence of Au_3-PEG_{500}-FA_{32} NPs inside a tubule cell of the UUO kidney. Energy-dispersive X-ray spectroscopy (EDX) analysis of Au_3-PEG_{500}-FA_{32} NPs in the kidney section verified the presence of gold.

Tissue-level distribution of Au_3-PEG_{500} and Au_3-PEG_{500}-FA_{32} NPs: Compartment isolation

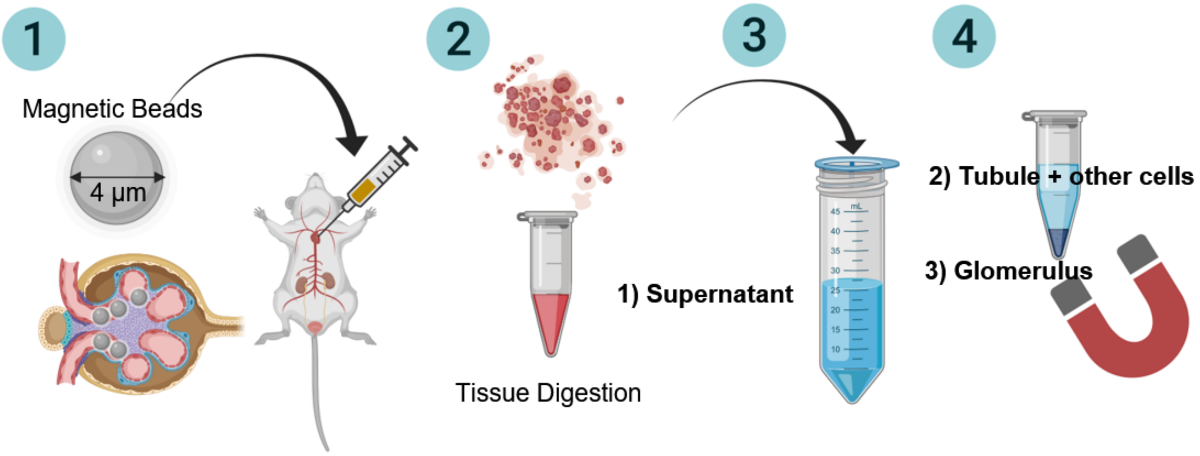

Fig. S24. Schematic illustration of the protocol for the compartment isolation of tubules and glomeruli. Illustration was created with Biorender. Initially, magnetic beads of ~4 µm in diameter were i.v. injected into mice and became clogged in the glomeruli. Since this method of compartment isolation uses collagenases for digesting the kidney tissues, the collagen in the interstitial space between tubules or glomeruli is expected to be dissolved during isolation. Any NP accumulated in the interstitial space would be released into the supernatant, and therefore the first supernatants were collected for ICP analysis. Finally, using an external magnetic field will allow for the separation of glomeruli from tubules.

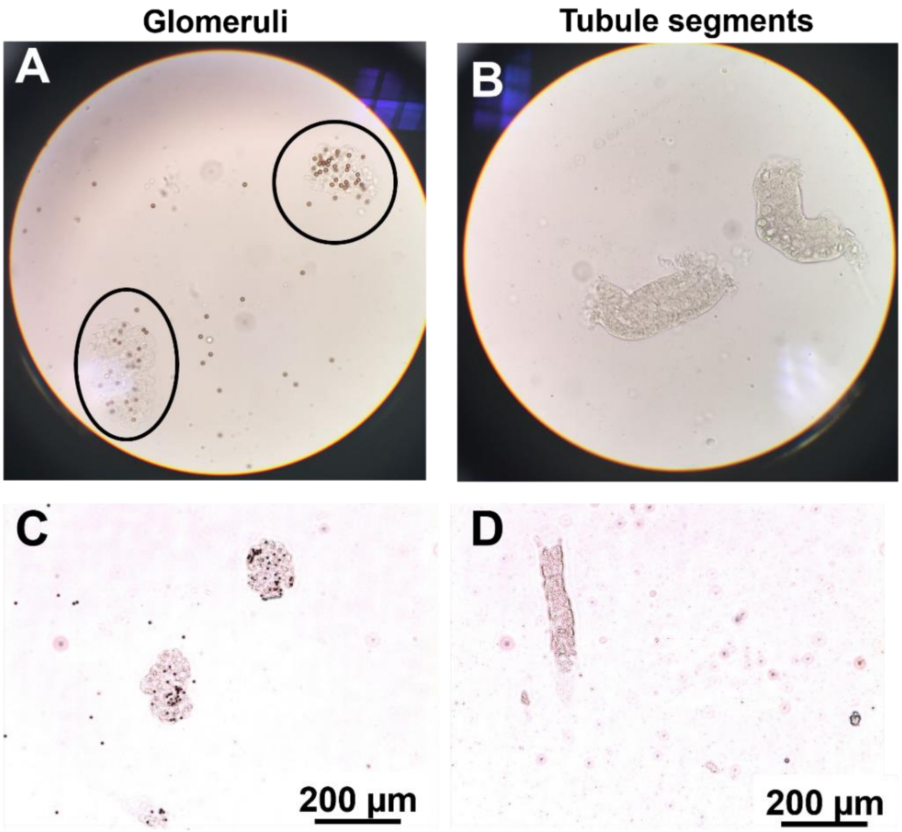

Fig. S25. Compartment isolation of the (A), (C) glomeruli and (B), (D) tubule segments plus tubulointerstitial cells by using Dynabeads. (A–B) Images of freshly isolated glomerulus and tubule segments. (C–D) Images of formalin-fixed glomerulus and tubules segments.

Effect of negative surface charge on the in vivo distribution of NPs in UUO mice

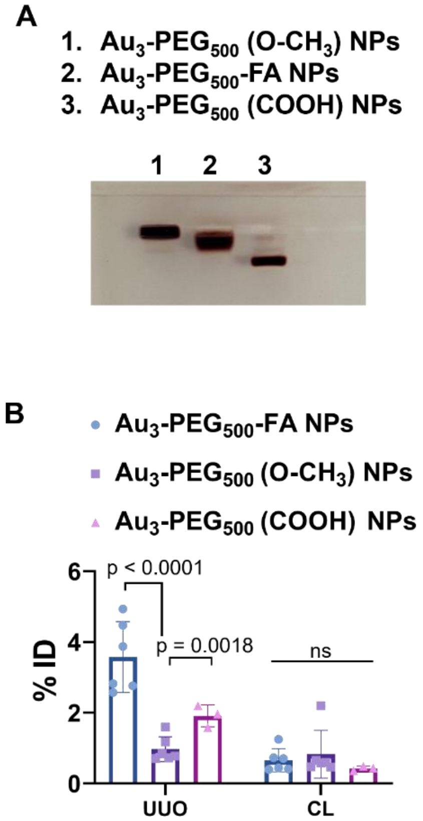

Fig. S26. (A) Agarose gel electrophoresis of (left) Au_3-PEG_{500} (O-CH_3), (middle) Au_3-PEG_{500}-FA_{32}, and (right) Au_3-PEG_{500}-COOH NPs. (B) Distribution to the UUO and CL kidneys 24 h post-injection into UUO mice. Statistical significance was evaluated using One-Way ANOVA and Tukey's multiple comparisons test. ns = not significant. Error bar denotes ±1 SD. Data are from n =3–6, across 1 experiment.

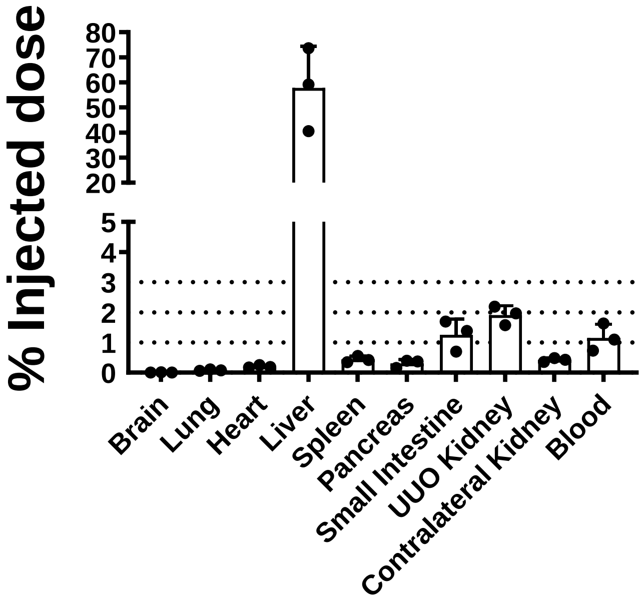

Fig. S27. Organ-level distribution of Au_3-PEG_{500}-COOH NPs 24 h post-injection into in UUO mice. Error bar denotes ±1 SD. Data are from n =3, across 1 experiment.

In vivo toxicity of Au_3-PEG_{500}-FA_{32} NPs in UUO mice

The immune cell counts and hematology indices of mice injected with Au_3-PEG_{500}-FA_{32} NPs and saline showed similar readings. These data suggest that Au_3-PEG_{500}-FA_{32} NPs are largely non-toxic, in line with the biocompatibility of non-cationic carriers (45).

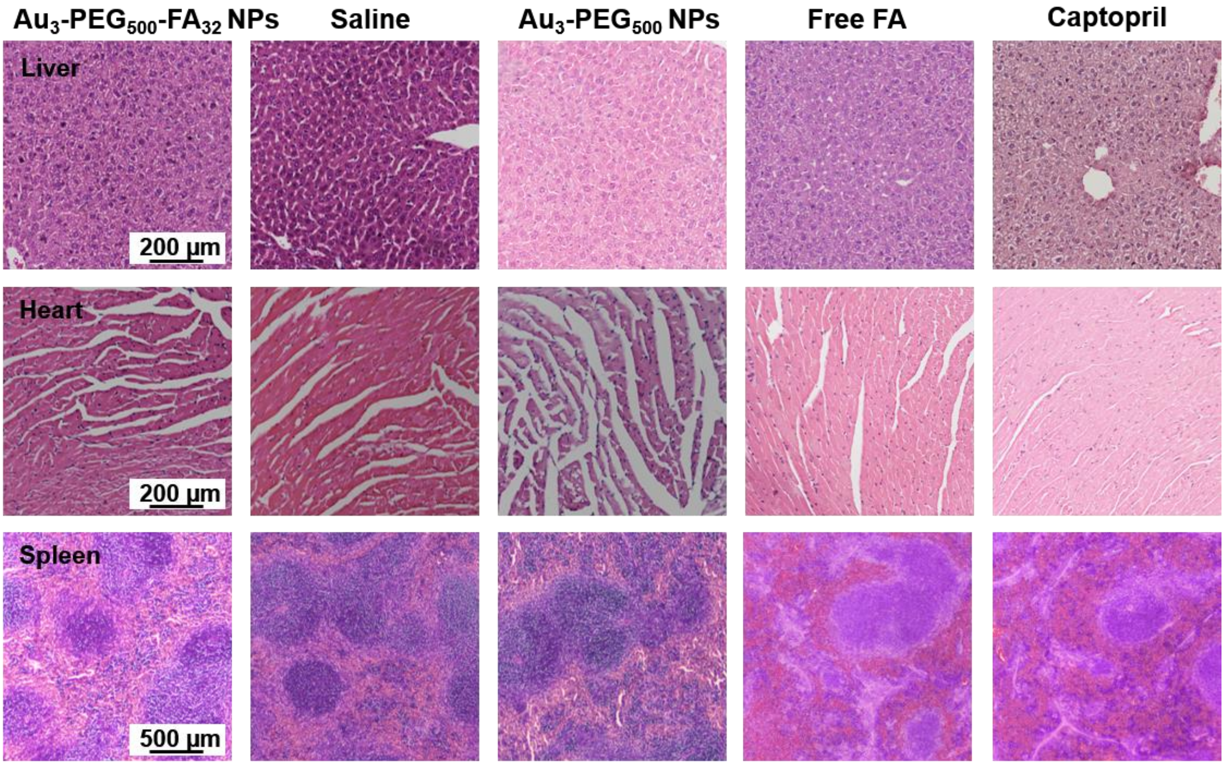

Fig. S28. Histological images of major organs in UUO mice following different treatments. All groups except Captopril were i.v. injected only once on Day 7 post-UUO surgery and sacrificed on Day 14. For the Captopril group, mice were i.v. injected daily starting from Day 7 till Day 14. Representative images from 2 tissue sections from n= 4 mice/group, across 1 experiment.

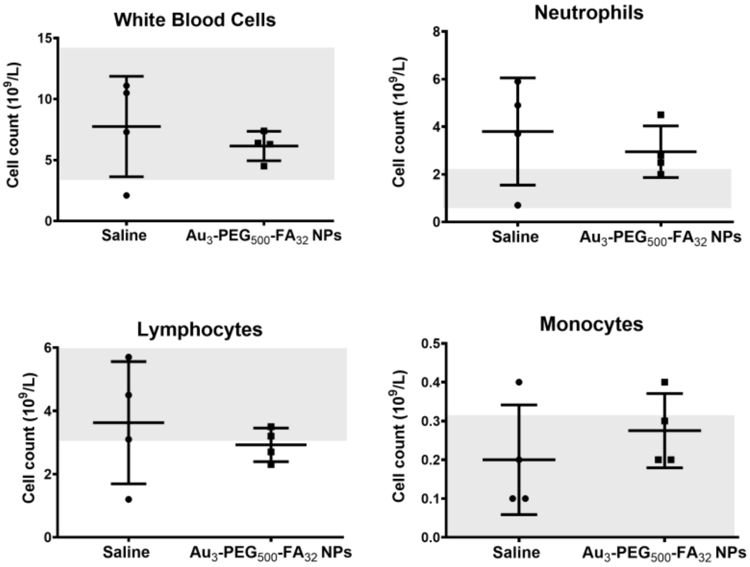

Fig. S29. Immune cell counts from blood drawn from UUO mice 7 days after i.v. injection of Au_3-PEG_{500}-FA_{32} NPs and saline (n=4 mice/group) (Day 14 after UUO surgery). Grey shaded area represents the normal range of cell counts for mice. Error bar denotes ± 1 SD. Data are from n =4, across 1 experiment.

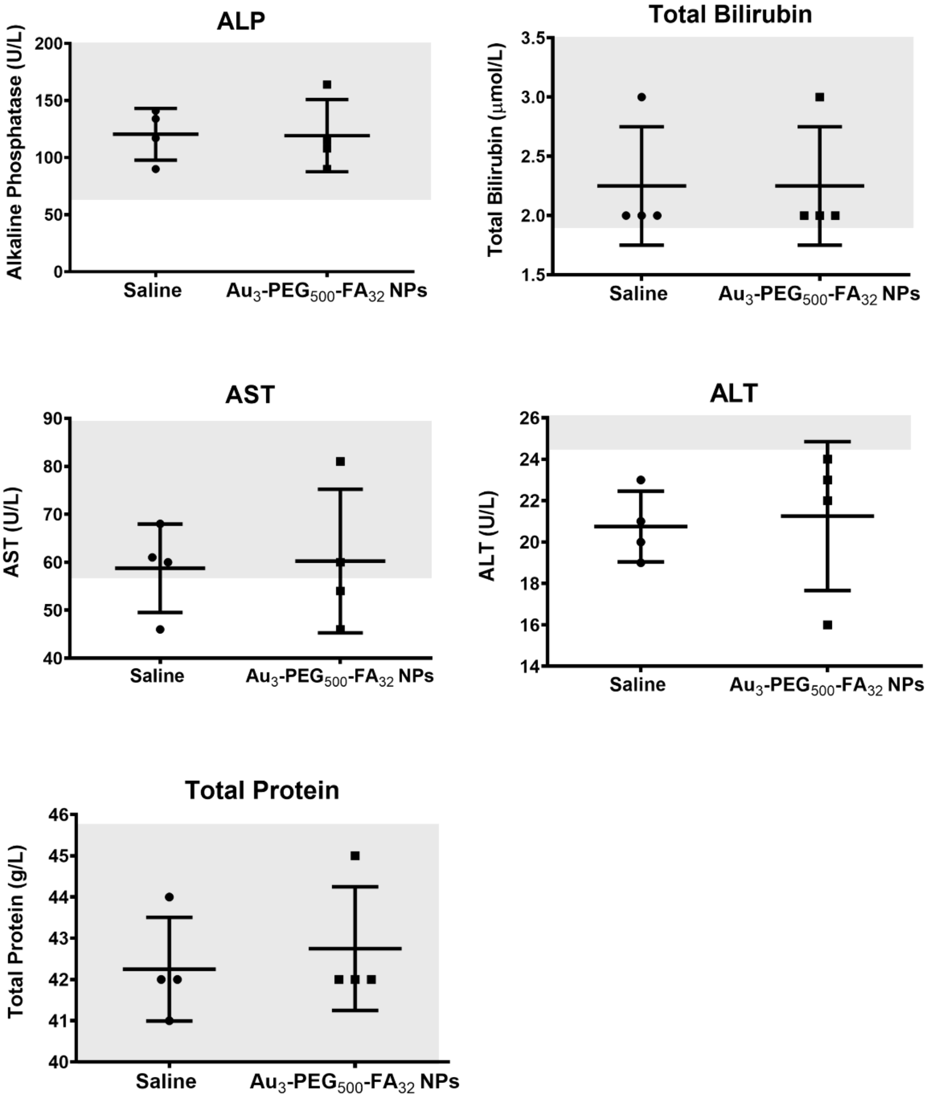

Fig. S30. Hepatotoxicity analysis of UUO mice 7 days after i.v. injection of Au_3-PEG_{500}-FA_{32} NPs and saline (or Day 14 after UUO surgery). Serum markers include alkaline phosphatase (ALP), total bilirubin, aspartate aminotransferase (AST), alanine transaminase (ALT) and total protein. Grey shaded area represents normal range for mice. Error bar denotes ± 1 SD. Data are from n =4, across 1 experiment.

.

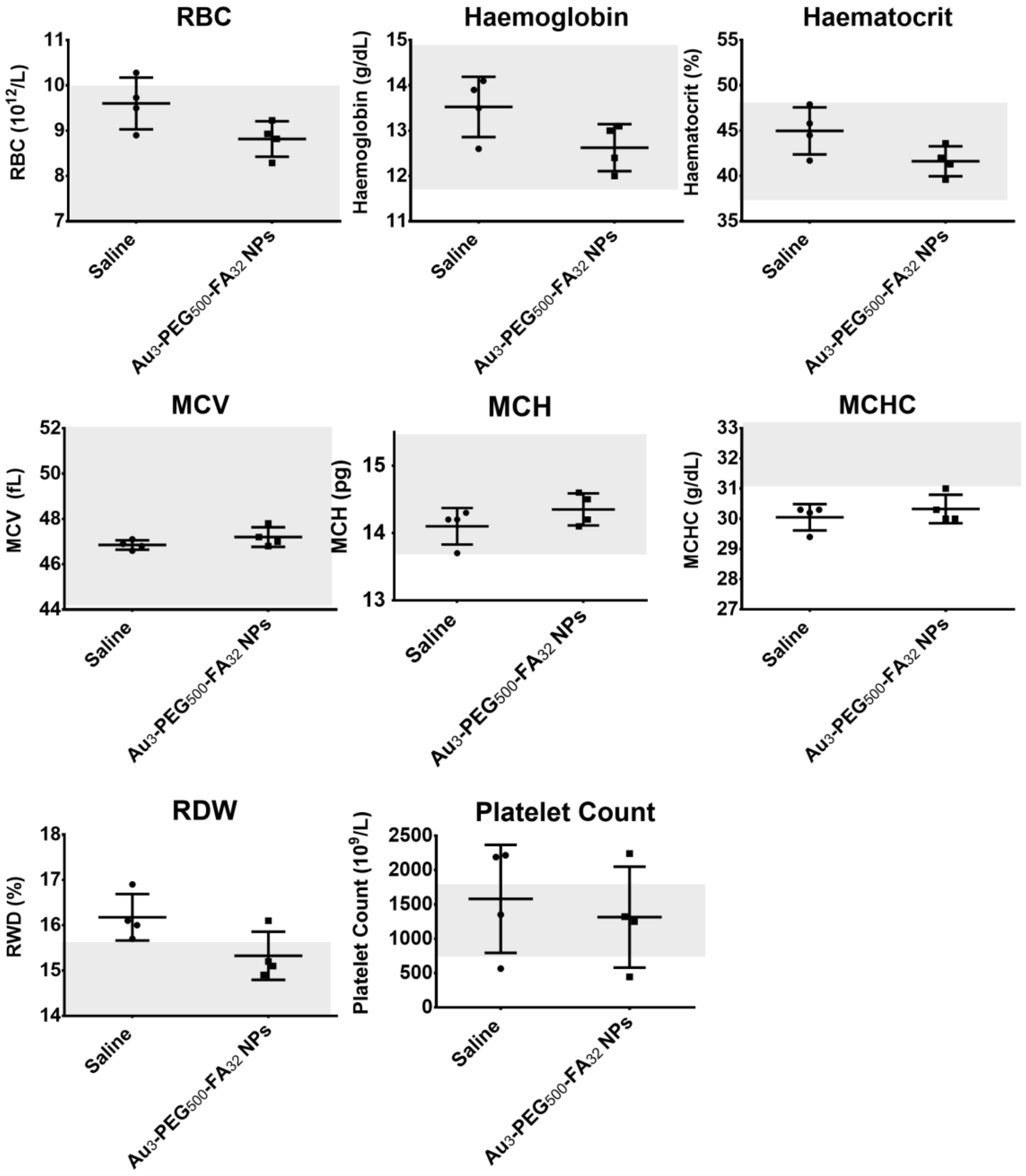

Fig. S31. Hematology analysis of UUO mice 7 days after i.v. injection of Au_3-PEG_{500}-FA_{32} NPs and saline (n=4 mice/group) (or Day 14 after UUO surgery). Parameters include red blood cell count (RBC), haemoglobin, haematocrit, mean corpuscular volume (MCV), mean corpuscular haemoglobin (MCH), mean corpuscular hemoglobin concentration (MCHC), red cell distribution width (RDW), and platelet count. Grey shaded area represents the normal range for mice. Error bar denotes ± 1 SD. Data are from n =4, across 1 experiment.

Kidney treatment requires high selectivity to the diseased renal cells without damaging the normal ones. IHC staining of the CL kidney (where fibrosis is not supposed to occur on Day 7 post-UUO surgery) with fibrosis markers show limited tissue damage on 7 d post-injection of Au_3-PEG_{500}-FA_{32} NPs. The serum levels of creatinine fall within the normal range for all treatments tested.

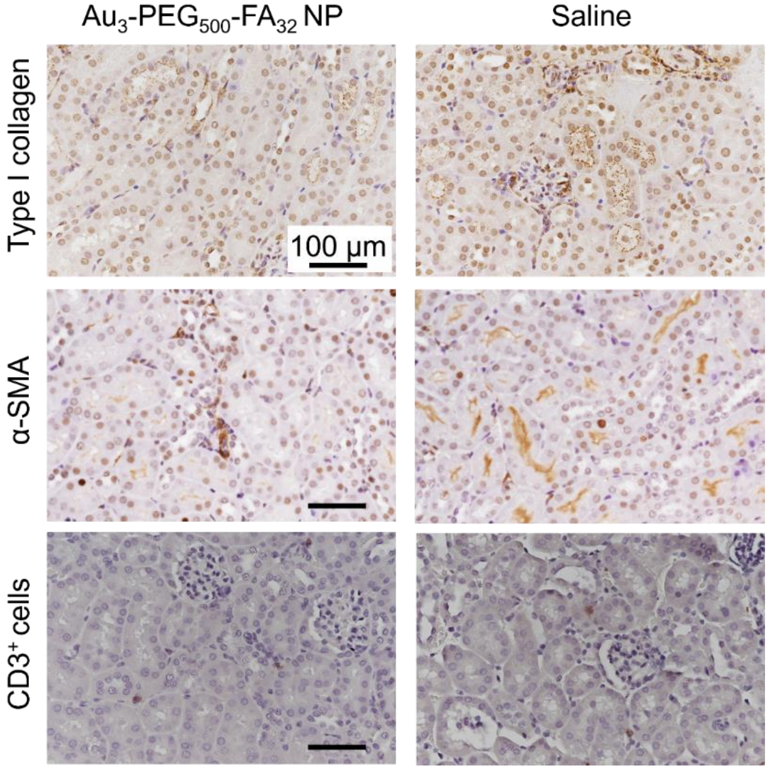

Fig. S32. IHC staining of the CL kidney treated from UUO mice that were i.v. injected with Au_3-PEG_{500}-FA_{32} NPs or saline 7 d post-treatment. α-SMA = α-smooth muscle actin. Representative images from 3 kidney sections from each mouse, n = 3 mice/group, across 1 experiment.

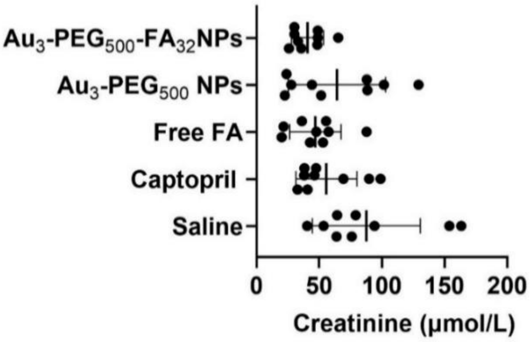

Fig. S33. Levels of blood creatinine in UUO mice upon different treatments. The normal range of serum creatinine concentration in mice is 7–296 µM (46). Error bar denotes ± 1 SD.

Endotoxin level of NPs

Table S5. Endotoxin level of Au_3-PEG_{500} NPs and of Au_3-PEG_{500}-FA_{32} NPs

Sample	Au_3-PEG_{500} NPs	Au_3-PEG_{500}-FA_{32} NPs
**Endotoxin level (EU/mL)**	0.01 ± 0.002	0.003 ± 0.001

All reported data represent mean ± 1 SD from four biological replicates.

Impact of the amount of gold NPs delivered to the UUO kidney on efficacy

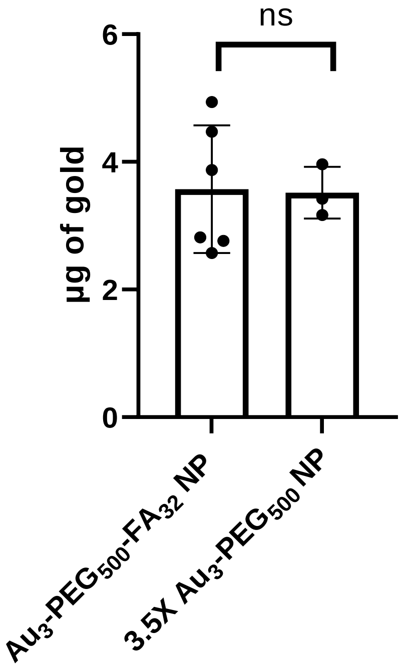

Fig. S34. I.v. delivery of Au_3-PEG_{500}-FA_{32} and Au_3-PEG_{500}-FA_{32} NPs to the UUO kidney 24 h post-injection. When the injection dose of Au_3-PEG_{500} NPs (360 µg) was ~3.5 times higher than that of Au_3-PEG_{500}-FA_{32} NPs (100 µg), the absolute amounts of Au in both cases delivered to the UUO kidney were similar (~3.6 µg). Statistical significance was evaluated by Mann-Whitney test, ns = not significant. Error bar denotes ±1 SD, with n = 3-6 across 3 experiments.

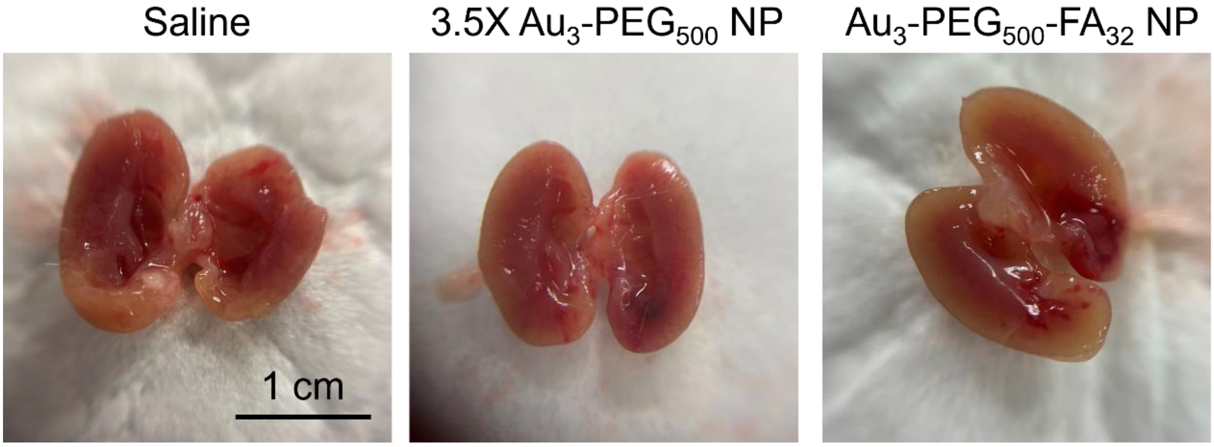

Fig. S35. Gross images of the UUO kidney from UUO mice treated with saline (left), Au_3-PEG_{500} NP at 3.5-times higher than the original dose (8.75 mg-Au/kg-mouse), and Au_3-PEG_{500}-FA_{32} NP at the original dose (2.5 mg-Au/kg-mouse). Representative images from n = 3 mice/group. When the absolute amounts of i.v. delivery of gold NPs to the UUO kidney were similar for Au_3-PEG_{500} NP and Au_3-PEG_{500} NP, both NP types alleviated tissue degeneration to similar extents.

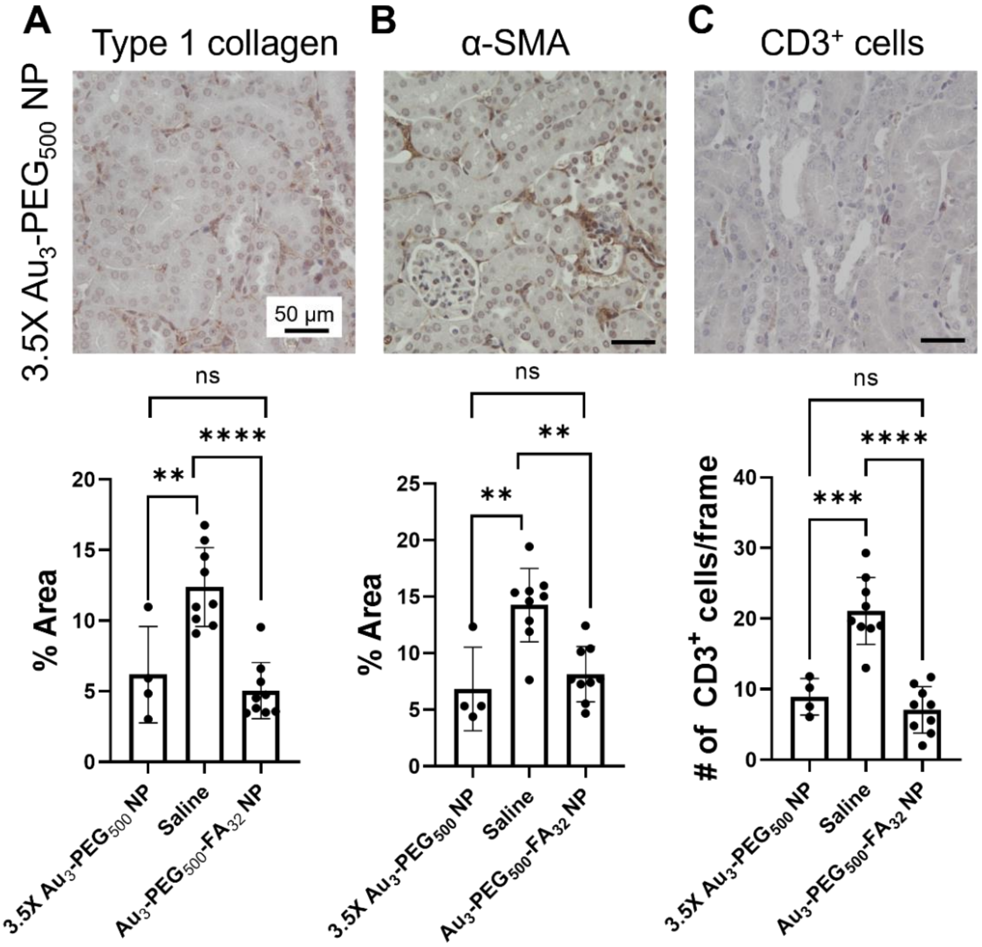

Fig. S36. Anti-fibrosis efficacy of Au_3-PEG_{500} NP at 3.5 times higher than the original dose (8.75 mg-Au/kg-mouse), and Au_3-PEG_{500}-FA_{32} NP at the original dose (2.5 mg-Au/kg-mouse). When the delivery of gold NPs to the UUO kidney in terms of absolute amount was similar for Au_3-PEG_{500} NP and Au_3-PEG_{500} NP, both Au_3-PEG_{500} and Au_3-PEG_{500} NPs reduced the (A) areas of collagen type I, (B) α-SMA, and (C) $CD3^+$ T cells in the UUO kidney to similar extents. Representative images 3 kidney sections from each mouse, n = 3 mice/group, across 1 experiment. Statistical significance was evaluated using One-Way ANOVA and Tukey's multiple comparisons test. * = p ≤0.05, ** = p ≤0.01,*** = p ≤0.001 **** = p ≤0.0001, ns = not significant. Error bar denotes ± 1 SD. Data are from n = 3-9, across 3 experiments.

Table S6. Expression level (FPKM) for type I collagen

Gene ID	Expression level (FPKM)								
	Saline			Au_3-PEG_{500}-FA_{32} NP			Free FA		
	1	2	3	1	2	3	1	2	3
12842	91.37	97.65	114.3	37.8	44.81	59.96	185.4	80.71	18.57

Validation of RNA-seq data using type I collagen as the marker

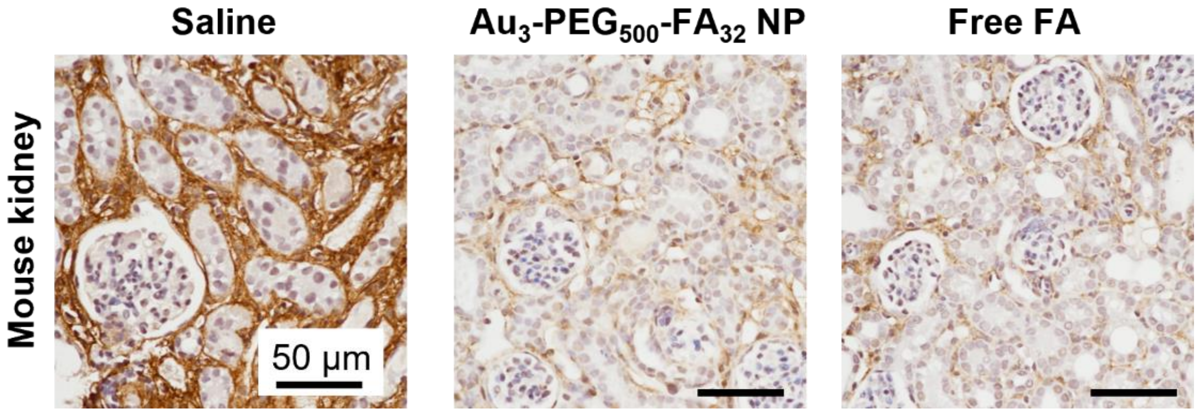

Fig. S37. IHC staining of type I collagen in the UUO kidney used for RNA-seq. UUO mice were i.v. injected with Au_3-PEG_{500}-FA_{32} NP, free FA, or saline on Day 7 post-UUO surgery and sacrificed on Day 9 (2 d post-injection). UUO kidneys were harvested for RNA extraction and RNA-seq. Representative images from 3 kidney sections from each mouse, n = 3 mice/group, across 1 experiment.

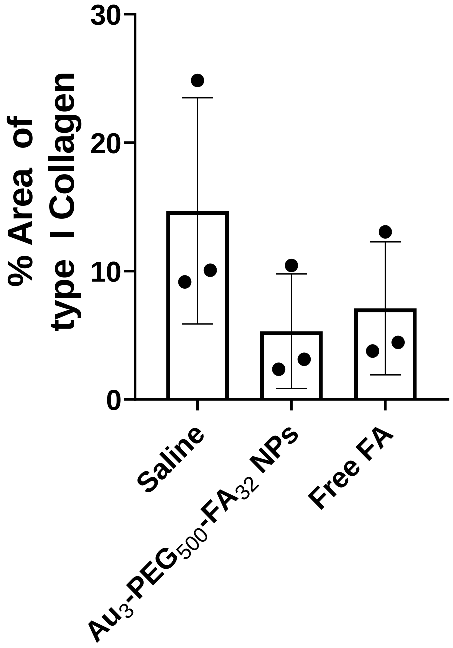

Fig. S38. IHC analysis of type I collagen expression in the same UUO kidneys harvested for RNA-seq 48 h post-injection. Both Au_3-PEG_{500}-FA_{32} NP and free FA groups show lower type I collagen protein expression than saline group, but the reduction in type I collagen was not statistically significant. Statistical significance was evaluated using One-Way ANOVA with Tukey's post hoc test for multiple comparison. All bars and error bars represent mean ± SD. Data are from n = 3, across 1 experiment.

Changes in transcriptomic-level gene expression upon injection of Au_3-PEG_{500}-FA_{32} NPs

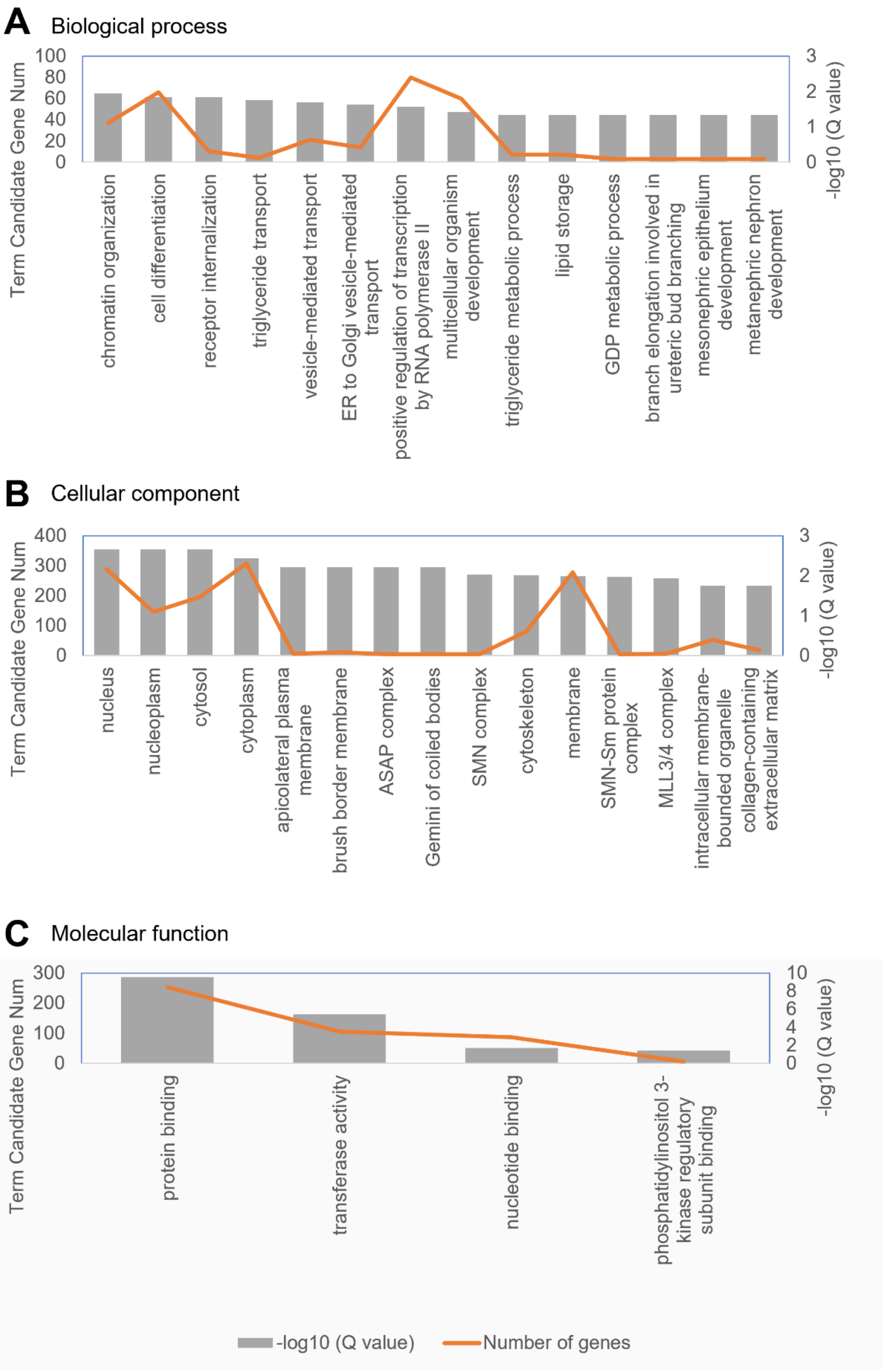

Fig. S39. Gene ontology (GO) term analysis. Statistically significant GO terms that were found in the Au_3-PEG_{500}-FA_{32} NP group with reference to the free FA group as baseline (Au_3-PEG_{500}-FA_{32} NP vs. free FA). These GO terms fall under three categories: (A) biological processes, (B) cellular component, and (C) molecular function. ($Q < 0.05$).

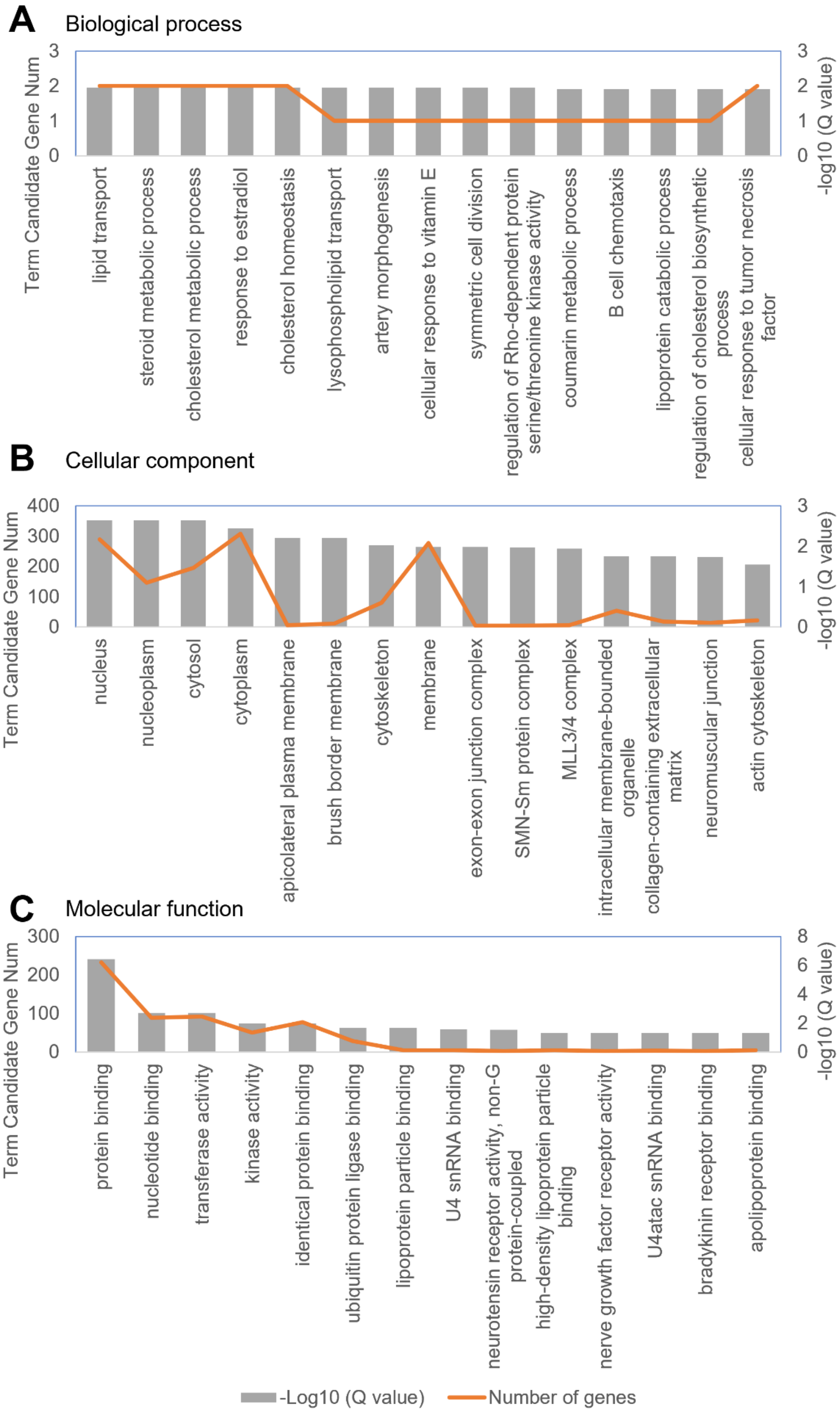

Fig. S40. Gene ontology (GO) term analysis. Statistically significant GO terms that were found in the Au$_3$-PEG$_{500}$-FA$_{32}$ NP group with reference to the saline group as baseline (Au$_3$-PEG$_{500}$-FA$_{32}$ NP vs. saline). These GO terms fall under three categories: (A) biological processes, (B) cellular component, and (C) molecular function. ($Q < 0.05$).

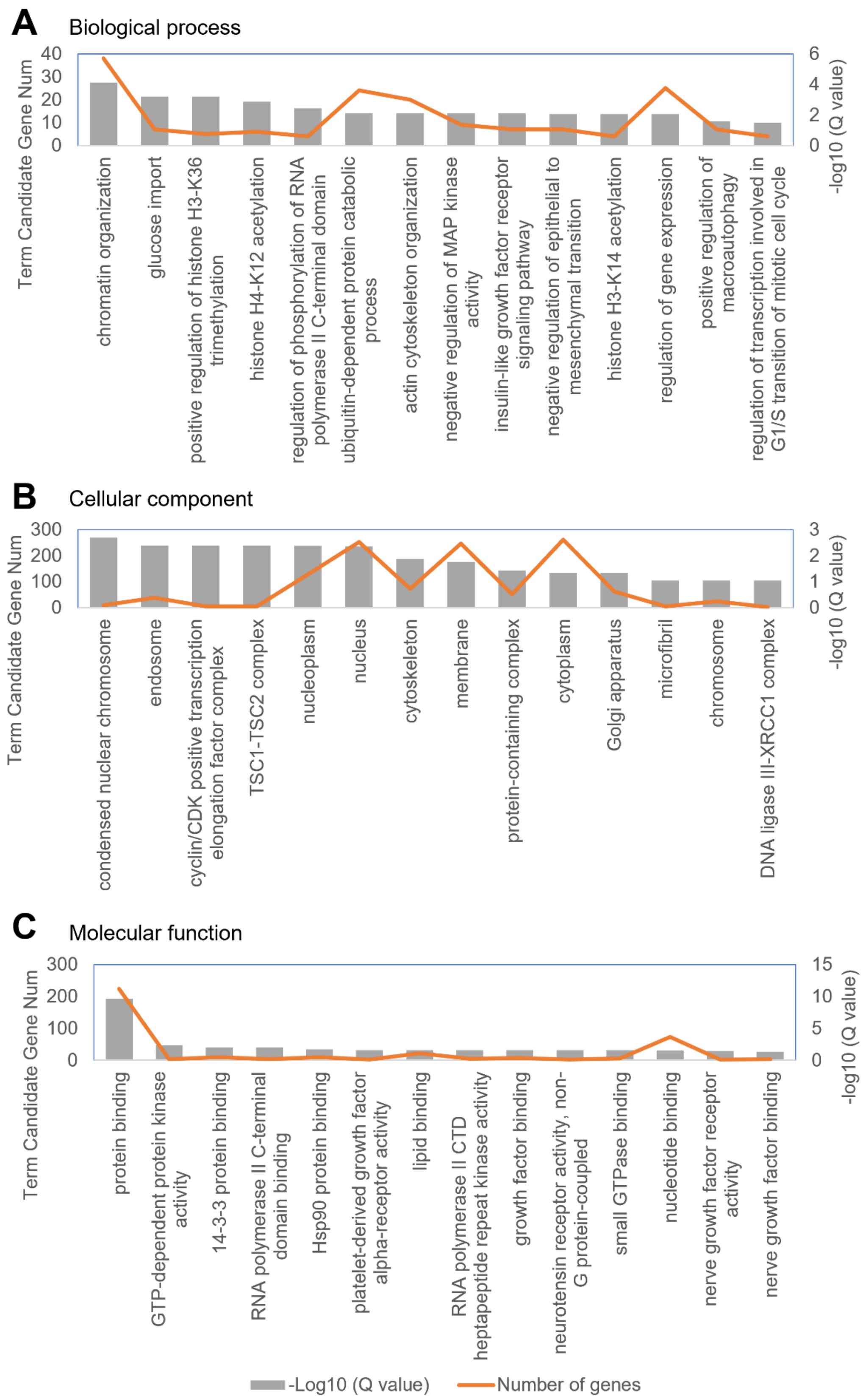

Fig. S41. Gene ontology (GO) term analysis. Statistically significant GO terms that were found in the free FA group with reference to the saline group as baseline (free FA vs. saline). These GO terms fall under three categories: (A) biological processes, (B) cellular component, and (C) molecular function. ($Q < 0.05$).

Table S7. List of kinase with >80% inhibition in their activities after treatment with 200 nM Au_3-PEG_{500}-FA_{32} NP using the Z'-LYTE™ Kinase Assay Kit (Thermo Fisher)

[ATP] tested (μM)	Kinase	Mean Percent Inhibition
Cascade:100	MAPK14 (p38 alpha)	100
Km app: 2	CSNK1E (CK1 epsilon)	100
Km app: 47.8	PLK3	100
Km app: 12	GRK4	100
Km app: 5	CSNK1G2 (CK1 gamma 2)	100
Km app: 29.6	PLK2	100
Km app: 2	CSNK1G1 (CK1 gamma 1)	100
Km app: 18	RPS6KA3 (RSK2)	100
Km app: 10	FGR	96
Km app: 4.4	CSNK1G3 (CK1 gamma 3)	95
Km app: 7	GSK3B (GSK3 beta)	94
Km app: 30	MELK	91
Cascade: 100	MAP2K6 (MKK6)	91
Km app: 2.5	MAPKAPK2	89
Km app: 10	AURKA (Aurora A)	89
Km app: 5	IKBKB (IKK beta)	87
Km app: 118.7	NEK1	87
Km app: 30	RPS6KA6 (RSK4)	87
Km app: 27	PDK1 Direct	86
Km app: 2	CSNK1A1 (CK1 alpha 1)	86
Km app: 56.2	NEK4	85
Km app: 4.7	DYRK3	85
Km app: 9	GSK3A (GSK3 alpha)	85
Km app: 27.7	TYRO3 (RSE)	85
Km app: 36	BTK	84
Km app: 18.5	FER	81
Km app: 123	EPHA8	80

A

NP	Kinase tested	IC_{50} (nM)
Au_3-PEG_{500} NP	GRK4	0.905
Au_3-PEG_{500} NP	RSK2	27
Au3-PEG_{500}-FA_{32} NP	GRK4	0.959
Au3-PEG_{500}-FA_{32} NP	RSK2	23.9

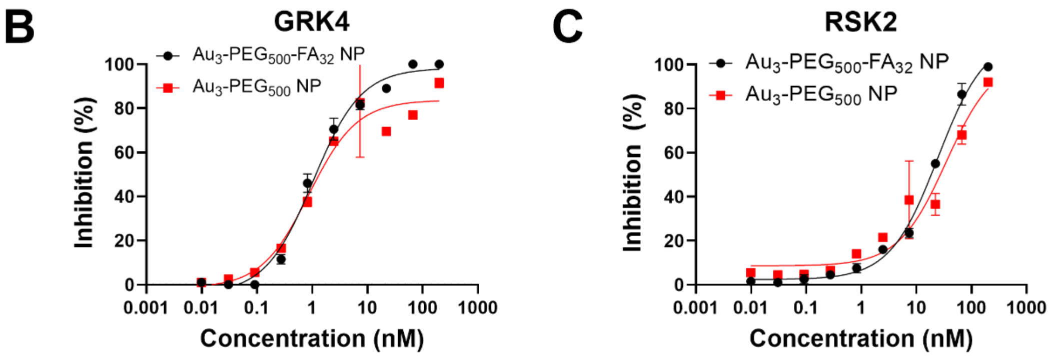

Fig. S42. (A) IC_{50} values of Au_3-PEG_{500} and Au_3-PEG_{500}-FA_{32} NP. (B)–(C) Percent inhibition of GRK4 and RSK2 by Au_3-PEG_{500}-FA_{32} (black) and Au_3-PEG_{500} NP (red) using the Z'-LYTE™ assay. All bars and error bars represent mean ± SD. Data are from n = 2, across 1 experiment.

SI References:

1. S. Huo, *et al.*, Ultrasmall gold nanoparticles behavior in vivo modulated by surface polyethylene glycol (PEG) grafting. *Bioconjug. Chem.* **28**, 239–243 (2017).
2. J. Piella, N. G. Bastús, V. Puntes, Size-controlled synthesis of sub-10-nanometer citrate-stabilized gold nanoparticles and related optical properties. *Chem. Mater.* **28**, 1066–1075 (2016).
3. N. G. Bastús, J. Comenge, V. Puntes, Kinetically controlled seeded growth synthesis of citrate-stabilized gold nanoparticles of up to 200 nm: Size focusing versus ostwald ripening. *Langmuir* **27**, 11098–11105 (2011).
4. W. Haiss, N. T. K. Thanh, J. Aveyard, D. G. Fernig, Determination of size and concentration of gold nanoparticles from UV-Vis spectra. *Anal. Chem.* **79**, 4215–4221 (2007).
5. T. Sawada, H. Fukuta, T. Serizawa, Preparation of biocomposite soft nanoparticles composed of poly(propylene oxide) and the polymer-binding peptides. *Processes* **8**, 859 (2020).
6. S. J. Hurst, A. K. R. Lytton-Jean, C. A. Mirkin, Maximizing DNA loading on a range of gold nanoparticle sizes. *Anal. Chem.* **78**, 8313–8318 (2006).
7. D. N. Benoit, *et al.*, Measuring the grafting density of nanoparticles in solution by analytical ultracentrifugation and total organic carbon analysis. *Anal. Chem.* **84**, 9238–9245 (2012).
8. D. Glancy, *et al.*, Characterizing the protein corona of sub-10 nm nanoparticles. *J. Control. Release* **304**, 102–110 (2019).
9. B. Yin, *et al.*, Intrapulmonary cellular-level distribution of inhaled nanoparticles with defined functional groups and its correlations with protein corona and inflammatory response. *ACS Nano* **13**, 14048–14069 (2019).
10. C. von Mering, *et al.*, STRING: a database of predicted functional associations between proteins. *Nucleic Acids Res.* **31**, 258–261 (2003).
11. H. M. Kang, *et al.*, Defective fatty acid oxidation in renal tubular epithelial cells has a key role in kidney fibrosis development. *Nat. Med.* **21**, 37–46 (2015).
12. S. Terryn, *et al.*, A primary culture of mouse proximal tubular cells, established on collagen-coated membranes. *Am. J. Physiol. Renal Physiol.* **293**, F476–F485 (2007).
13. S. Yamashita, Heat-induced antigen retrieval: Mechanisms and application to histochemistry. *Prog. Histochem. Cytochem.* **41**, 141–200 (2007).
14. M. Takemoto, *et al.*, A new method for large scale isolation of kidney glomeruli from mice. *Am. J. Pathol.* **161**, 799–805 (2002).
15. J. C. Y. Kah, M. C. Olivo, C. G. L. Lee, C. J. R. Sheppard, Molecular contrast of EGFR expression using gold nanoparticles as a reflectance-based imaging probe. *Mol. Cell. Probes* **22**, 14–23 (2008).
16. E. Mocan, O. Tagadiuc, V. Nacu, Aspects of collagen isolation procedure. *Clinical research studies* **2**, 3–5 (2011).
17. K. Koike, *et al.*, Protective role of JAK/STAT signaling against renal fibrosis in mice with unilateral ureteral obstruction. *Clin. Immunol.* **150**, 78–87 (2014).
18. C. Yang, *et al.*, Chitosan/siRNA nanoparticles targeting cyclooxygenase type 2 attenuate unilateral ureteral obstruction-induced kidney injury in mice. *Theranostics* **5**, 110–123 (2015).
19. Y. Morishita, *et al.*, Delivery of microRNA-146a with polyethylenimine nanoparticles inhibits renal fibrosis in vivo. *Int. J. Nanomedicine* **10**, 3475–3488 (2015).
20. R. Li, *et al.*, Targeted delivery of celastrol to renal interstitial myofibroblasts using fibronectin-binding liposomes attenuates renal fibrosis and reduces systemic toxicity. *J. Control. Release* **320**, 32–44 (2020).
21. X. D. Lai, X. R. Geng, L. A. Tan, J. Q. Hu, S. B. Wang, A pH-responsive system based on fluorescence enhanced gold nanoparticles for renal targeting drug delivery and fibrosis therapy. *Int. J. Nanomedicine* **15**, 5613–5627 (2020).
22. S. P. Wei, *et al.*, Ultrasound assisted a peroxisome proliferator-activated receptor (PPAR)gamma agonist-loaded nanoparticle-microbubble complex to attenuate renal interstitial fibrosis. *Int. J. Nanomedicine* **15**, 7315–7327 (2020).
23. M. A. Saifi, C. S. Peddakkulappagari, A. Ahmad, C. Godugu, Leveraging the pathophysiological alterations of obstructive nephropathy to treat renal fibrosis by cerium oxide nanoparticles. *ACS Biomater. Sci. Eng.* **6**, 3563–3573 (2020).

24. H. Huang, *et al.*, Farnesylthiosalicylic acid-loaded albumin nanoparticle alleviates renal fibrosis by inhibiting Ras/Raf1/p38 signaling pathway. *Int. J. Nanomedicine* **16**, 6441–6453 (2021).
25. Q. Liu, *et al.*, Gypenoside XLIX loaded nanoparticles targeting therapy for renal fibrosis and its mechanism. *Eur. J. Pharmacol.* **910**, 174501 (2021).
26. H. T. Cheng, *et al.*, Delivery of sorafenib by myofibroblast-targeted nanoparticles for the treatment of renal fibrosis. *J. Control. Release* **346**, 169–179 (2022).
27. M. Wang, *et al.*, Ceria nanoparticles ameliorate renal fibrosis by modulating the balance between oxidative phosphorylation and aerobic glycolysis. *J. Nanobiotechnology* **20**, 3 (2022).
28. H. Sun, *et al.*, Kidney-targeted drug delivery system based on metformin-grafted chitosan for renal fibrosis therapy. *Mol. Pharm.* **19**, 3075–3084 (2022).
29. F. Tong, *et al.*, Quercetin nanoparticle complex attenuated diabetic nephropathy via regulating the expression level of ICAM-1 on endothelium. *Int. J. Nanomedicine* **12**, 7799–7813 (2017).
30. K. Manna, *et al.*, Amelioration of diabetic nephropathy using pomegranate peel extract-stabilized gold nanoparticles: assessment of NF-kappa B and Nrf2 signaling system. *Int. J. Nanomedicine* **14**, 1753–1777 (2019).
31. T. T. Tang, *et al.*, Employing macrophage-derived microvesicle for kidney-targeted delivery of dexamethasone: An efficient therapeutic strategy against renal inflammation and fibrosis. *Theranostics* **9**, 4740–4755 (2019).
32. G. Alomari, *et al.*, Gold nanoparticles attenuate albuminuria by inhibiting podocyte injury in a rat model of diabetic nephropathy. *Drug Deliv. Transl. Res.* **10**, 216–226 (2020).
33. Z. Lu, *et al.*, Nanoparticle-mediated delivery of emodin via colonic irrigation attenuates renal injury in 5/6 nephrectomized rats. *Front. Pharmacol.* **11**, 606227 (2021).
34. L. Liu, *et al.*, Fe_3O_4 magnetic nanoparticles ameliorate albumin-induced tubulointerstitial fibrosis by autophagy related to Rab7. *Colloids Surf. B Biointerfaces* **198**, 111470 (2021).
35. P. C. Fang, *et al.*, Dual-regulated functionalized liposome-nanoparticle hybrids loaded with dexamethasone/TGF beta 1-siRNA for targeted therapy of glomerulonephritis. *ACS Appl. Mater. Interfaces* **14**, 307–323 (2022).
36. L. T. Zhou, *et al.*, Co-delivery of dexamethasone and captopril by alpha 8 integrin antibodies modified liposome-PLGA nanoparticle hybrids for targeted anti-inflammatory/anti-fibrosis therapy of glomerulonephritis. *Int. J. Nanomedicine* **17**, 1531–1547 (2022).
37. R. L. Chevalier, M. S. Forbes, B. A. Thornhill, Ureteral obstruction as a model of renal interstitial fibrosis and obstructive nephropathy. *Kidney Int.* **75**, 1145–1152 (2009).
38. A. C. Ucero, *et al.*, Unilateral ureteral obstruction: beyond obstruction. *Int. Urol. Nephrol.* **46**, 765–776 (2014).
39. M. S. Forbes, *et al.*, Fight-or-flight: murine unilateral ureteral obstruction causes extensive proximal tubular degeneration, collecting duct dilatation, and minimal fibrosis. *Am. J. Physiol. Renal Physiol.* **303**, F120–F129 (2012).
40. J. S. Suk, Q. Xu, N. Kim, J. Hanes, L. M. Ensign, PEGylation as a strategy for improving nanoparticle-based drug and gene delivery. *Adv. Drug Deliv. Rev.* **99**, 28–51 (2016).
41. C. K. W. Chan, *et al.*, Recent advances in managing atherosclerosis via nanomedicine. *Small* **14**, 1702793 (2018).
42. M. E. M. Dolman, S. Harmsen, G. Storm, W. E. Hennink, R. J. Kok, Drug targeting to the kidney: Advances in the active targeting of therapeutics to proximal tubular cells. *Adv. Drug Deliv. Rev.* **62**, 1344–1357 (2010).
43. H. Sarin, Physiologic upper limits of pore size of different blood capillary types and another perspective on the dual pore theory of microvascular permeability. *J. Angiogenes. Res.* **2**, 14 (2010).
44. B. Du, M. Yu, J. Zheng, Transport and interactions of nanoparticles in the kidneys. *Nat. Rev. Mater.* **3**, 358–374 (2018).
45. L. W. C. Ho, Y. Liu, R. Han, Q. Bai, C. H. J. Choi, Nano-cell interactions of non-cationic bionanomaterials. *Acc. Chem. Res.* **52**, 1519–1530 (2019).
46. A. Keppler, *et al.*, Plasma creatinine determination in mice and rats: An enzymatic method compares favorably with a high-performance liquid chromatography assay. *Kidney Int.* **71**, 74–78 (2007).
